# Excited-State Lifetime Modulation by Twisted and Tilted Molecular Design in Carbene-Metal-Amide Photoemitters

**DOI:** 10.1021/acs.chemmater.2c01938

**Published:** 2022-08-04

**Authors:** Qinying Gu, Florian Chotard, Julien Eng, Antti-Pekka M. Reponen, Inigo J. Vitorica-Yrezabal, Adam W. Woodward, Thomas J. Penfold, Dan Credgington, Manfred Bochmann, Alexander S. Romanov

**Affiliations:** † Department of Physics, Cavendish Laboratory, Cambridge University, Cambridge CB3 0HF, U.K.; ‡ School of Chemistry, University of East Anglia, Norwich Research Park, Norwich, NR4 7TJ, U.K.; § School of Chemistry, Newcastle University, Bedson Building, Newcastle upon Tyne, NE1 7RU, U.K.; ∥ Department of Chemistry, University of Manchester, Manchester, M13 9PL, U.K.

## Abstract

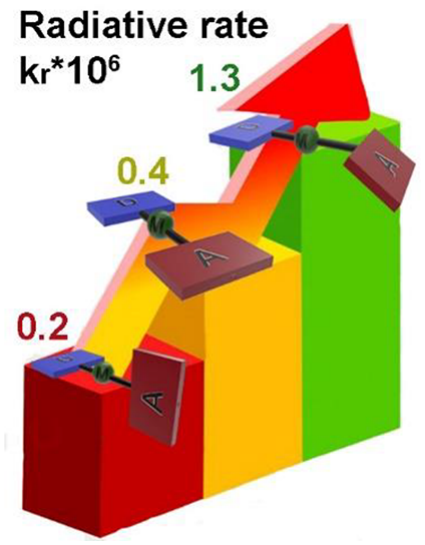

Carbene–metal–amides (CMAs) are an emerging class of photoemitters based on a linear donor–linker–acceptor arrangement. They exhibit high flexibility about the carbene–metal and metal–amide bonds, leading to a conformational freedom which has a strong influence on their photophysical properties. Herein we report CMA complexes with (1) nearly coplanar, (2) twisted, (3) tilted, and (4) tilt-twisted orientations between donor and acceptor ligands and illustrate the influence of preferred ground-state conformations on both the luminescence quantum yields and excited-state lifetimes. The performance is found to be optimum for structures with partially twisted and/or tilted conformations, resulting in radiative rates exceeding 1 × 10^6^ s^–1^. Although the metal atoms make only small contributions to HOMOs and LUMOs, they provide sufficient spin–orbit coupling between the low-lying excited states to reduce the excited-state lifetimes down to 500 ns. At the same time, high photoluminescence quantum yields are maintained for a strongly tilted emitter in a host matrix. Proof-of-concept organic light-emitting diodes (OLEDs) based on these new emitter designs were fabricated, with a maximum external quantum efficiency (EQE) of 19.1% with low device roll-off efficiency. Transient electroluminescence studies indicate that molecular design concepts for new CMA emitters can be successfully translated into the OLED device.

## Introduction

Thermally activated delayed fluorescence (TADF)^1−31^ has emerged as a competitive approach for realizing highly efficient organic light emitting diodes (OLEDs) for display and lighting applications. However, one of the principle challenges in designing and optimizing TADF materials is the apparent orthogonality of the functional properties required. High efficiency requires the realization of a small energy gap (Δ*E*_ST_) between the lowest excited singlet and triplet states, which is usually achieved by diminishing overlap of the HOMO and LUMO wave functions.^32−35^ However, this approach reduces the radiative rate, thus increasing the excited-state lifetime of these emitters. Long excited-state lifetimes can be detrimental to the OLED operational half-life and the efficiency at high brightness. Consequently, establishing a detailed structure–property relationship to enable fine-tuning of the emission properties is essential.

One approach exploits steric interactions between the ligands to control their conformational dynamics and optimize the balance between a small Δ*E*_ST_ and a high radiative rate. A similar approach has been employed for organic molecules exhibiting TADF;^35,36^ however, although twisted donor–acceptor (D–A) structures have been shown to exhibit higher radiative rates, they also give rise to low-lying local triplet states, which open nonradiative decay pathways. Indeed, for metal-free organic materials, a twisted molecular design can often result in room temperature phosphorescence and excited-state lifetimes which are too long for OLED applications.^37^ In contrast, for coordination complexes, it has been shown that the dual presence of TADF and phosphorescence can be used to reduce the excited-state lifetimes^38^ because of the high spin–orbit coupling (SOC) arising from the heavy atom effect.^32−34^

Linear coinage metal complexes of the general structure L-M-X (M = Cu, Ag, Au; X = anionic ligand)^39−41^ are an emerging class of new, highly efficient photoemitters and have recently been shown to achieve near-unity photoluminescence quantum yields as well as triplet lifetimes in the submicrosecond range.^40^ In particular, complexes where L = cyclic (alkyl)(amino)carbene (CAAC)^42,43^ and X = carbazolate have enabled the realization of OLED devices with near-100% internal and >25% external quantum efficiency (EQE) and submicrosecond lifetimes, via a delayed luminescence mechanism.^44−49^ Experimental and computational studies^44,48,50−56^ relate the device efficiency to the angular dependence of the exchange energy Δ*E*_ST_, which approaches zero at high torsion angles between amide donor and CAAC acceptor as a result of the diminishing overlap between HOMO and LUMO wave functions.

**Figure 1:**
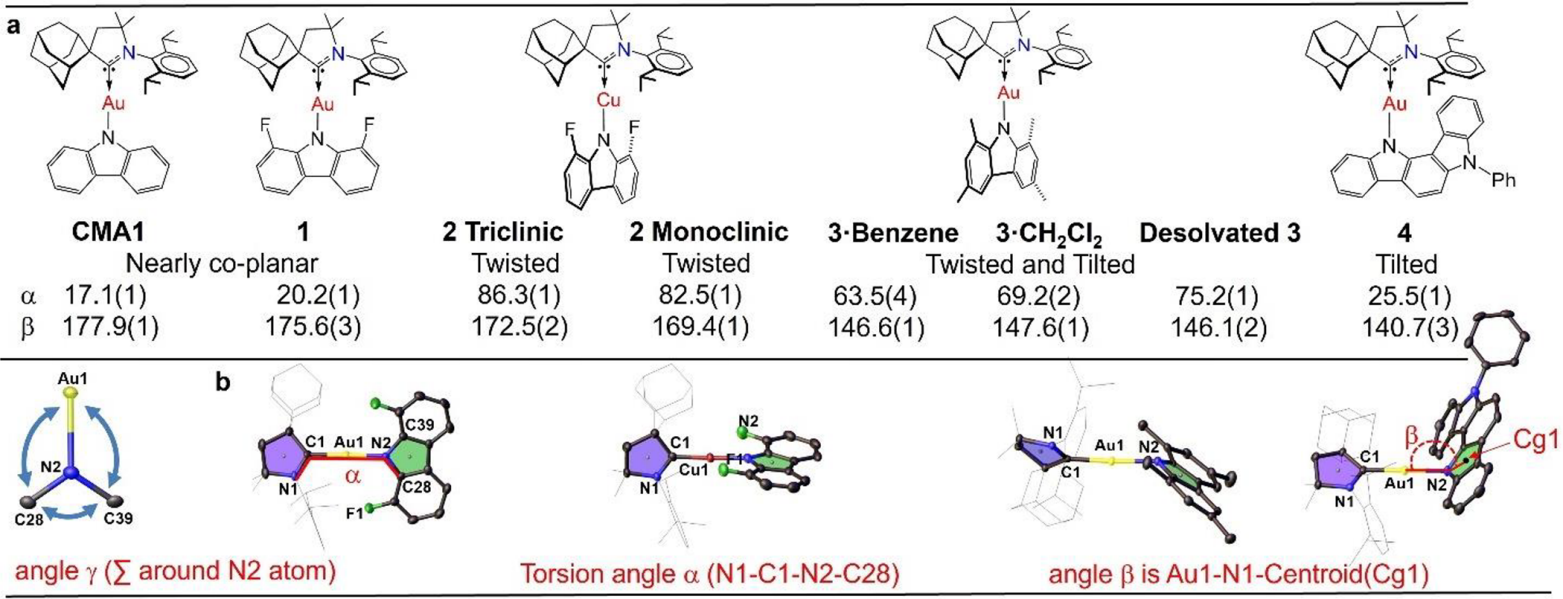
(a) Chemical structures of **1**–**4**; (b) Crystal structures of **1**–**4** with ellipsoids at 50% probability, showing the atomic numbering scheme with the relative orientations of CAAC (blue) and carbazolate (green) ligand planes, illustrating the dihedral twist angles α = N1–C1–N2–C28; tilt angles β, defined as the angle M-N-Cz(centroid) and angle γ defined as a sum of M–N2–C28 + M–N2–C39 + C28–N2–C39. The substituents on the carbene are shaded gray for clarity.

The ground-state geometry of most carbene–metal–amide (CMA) emitters reported to-date is linear, with a near-coplanar orientation of the carbene (acceptor) and carbazolate (Cz, donor) ligands.^44−51^ Here we demonstrate a molecular design with noncoplanar ground-state geometries (Figure 1), by introducing a twist angle α (N1–C1–N2–C28), a tilt angle β (Au1–N2-Centroid Cg1), and/or the pyramidalization of the Cz-N atom (as measured by the angle sum γ around N; γ = M–N2–C28 + M–N2–C39 + C28–N2–C39, Figure 1) and show how these geometric parameters can be used to control the photophysical properties. We report the synthesis of a stable copper complex with fully twisted conformation, demonstrate the effect of conformational preferences on the HOMO–LUMO overlap and thus Δ*E*_ST_, and explore the impact on the excited-state lifetimes with a proof of concept demonstration of highly efficient OLED devices based on new CMA materials with noncoplanar molecular designs.

## Results and Discussion

### Synthesis and Characterization

The new 1,8-difluorocarbazole ligand was prepared in two steps via palladium-catalyzed intermolecular coupling of 2-fluoroaniline with 2-bromo-1-chloro-3-fluorobenzene and C–H activation reactions, with an overal yield of 67%, see experimental part. Complexes **1**–**4** (Figure 1) were prepared by reacting (^Ad^CAAC)MCl (M = Cu, Au) with 1,8-difluorocarbazole (**1**, **2**), 1,3,6,8-tetramethylcarbazole (**3**) or 5,12-dihydro-5-phenyl-indolo[3,2-*a*]carbazole (**4**) as the donor ligands, following our previously published methods.^40,44^ All complexes have been characterized by ^1^H, ^13^C{^1^H}, and ^19^F{^1^H} NMR spectroscopy, high-resolution mass spectrometry (HRMS), and elemental analysis. In DCM-d^2^ solution, compounds **1** and **2** show fast rotation about the M–N bond on the NMR time scale, which is not frozen out on cooling to −80 °C, resulting in ^19^F{1H} resonances as a singlet and ^19^F as a doublet of doublets due to splitting on the carbazole hydrogens (Figure S1 and S2). Complexes **1**–**3** have high solubility in THF, DCM, 1,2-difluorobenzene, and toluene and are sparingly soluble in hexanes. Unlike **1**–**3**, complex **4** has good solubility in DCM and moderate solubility in warm aromatic solvents, but it is insoluble in hexanes. The thermal stability of twisted complexes **2** and **3** is 20–30 °C less compared with nearly coplanar **1** and tilted **4** (Figure S3).

### Electrochemistry

Cyclic voltammetry of **1**–**4** in THF solution shows quasi-reversible reduction and irreversible oxidation processes for all complexes (Table 1, Figure S4 and S5). The gold complexes **1**, **3**, and **4** show small variations in reduction *E*_1/2_ values, – 2.74 ± 0.06 V, resulting in similar LUMO energy levels of −2.75 ± 0.05 eV. The copper complex **2** exhibits a reduction *E*_1/2_ value at −2.99 V, hence the LUMO is destabilized by 0.15 eV compared with gold complexes, similar to other copper CMA complexes.^44,49^ The reduction process is largely localized on the carbene ligand with minor contribution of the coinage metal as can be seen by the minor difference in *E*_1/2_ values (200 mV) between gold **1** and copper **2** complexes. The oxidation process is centered on the carbazole ligand, with *E*_p_ values decreasing from +0.55 V for **1** to +0.09 V for **3** in line with increasing electron-donor effect of the substituents on the carbazole unit.

**Table 1:** Formal Electrode Potentials (Peak Position *E*_p_ for Irreversible and *E*_1/2_ for Quasi-Reversible Processes (*), *V*, vs FeCp_2_), Onset Potentials (*E, V*, vs FeCp_2_), Peak-to-Peak Separation in Parentheses for Quasi-Reversible Processes (Δ*E*_p_ in mV), *E*_HOMO_/*E*_LUMO_ (eV) and Band Gap Values (Δ*E*, eV) for the Redox Changes Exhibited by Copper and Gold Complexes^a^

	reduction			oxidation				
complex	*E*_first_	*E*_onset red_	*E*_LUMO_ (eV)	*E*_first_	*E*_onset ox_	*E*_second_	*E*_HOMO_ (eV)	Δ*E* (eV)
**CMA1**	–2.80* (166)	–2.71	–2.68	+0.25	+0.14	–	–5.53	2.85
**1** (Au)	–2.79* (171)	–2.68	–2.71	+0.55	+0.42	–	–5.81	3.10
**2** (Cu)	–2.99* (165)	–2.89	–2.50	+0.40	+0.23	–	–5.62	3.12
**3**	–2.74* (173)	–2.65	–2.74	+0.09	–0.04	–	–5.35	2.61
**4**	–2.66* (162)	–2.58	–2.81	+0.22	+0.09	+0.63	–5.48	2.67

a In THF solution, recorded using a glassy carbon electrode, concentration 1.4 mM, supporting electrolyte [^*n*^Bu_4_N][PF_6_] (0.13 M), measured at 0.1 V s^–1^. *E*_HOMO_*=* −(*E*_onset ox Fc/Fc+_*+* 5.39) eV; *E*_LUMO_*=* −(*E*_onset red Fc/Fc+_*+* 5.39) eV.

Complex **4** shows a second irreversible oxidation wave at +0.63 V (overlaps with the solvent discharge process, Figure S5), which is likely centered on the benzocarbazole moiety based on similarity of the redox profile and oxidation potential measured for the CMA1 complex.^16^ The quasi-reversibility of the reduction peak is witnessed by the peak-to-peak separation Δ*E*_p_ ca. 170 mV (at 100 mV/s), which deviates from the ideal value of 59 mV for a one-electron reversible couple (Table 1).

The resulting HOMO energy values increase from 5.81 eV for **1**, −5.48 eV for **4** and to −5.35 eV for **3** (Table 1). The electron-withdrawing fluoride moiety stabilizes the HOMO energy level, resulting in a blue shift of the absorption and emission properties as seen for the complexes **1** and **2**, whereas an electron-donating group such as methyl results in complex **3** showing an opposite effect (destabilization the HOMO energy level and red shift). This is in-line with our DFT calculations suggesting that the HOMO–LUMO energy gap is decreasing from complex **1** (3.1 eV) > CMA1 (2.8 eV) > complex **3** and **4** (2.6 eV).

### X-ray Diffraction Experiments

The crystal structures for complexes **1**–**4** were determined by single-crystal X-ray diffraction to reveal the role of the steric congestion imposed by various carbazole substituents and size of the metal atom on the relative orientation of the carbazole and carbene ligand planes in the CMA molecule. Additional crystallizations of the new compounds were carried out using various solvents, in an effort to evaluate the influence of the solvent on the structural variation in the crystal, and to link this with the CMA photophysical parameters in the solid state. The crystal structures revealed that the gold complex **1** has the nearly coplanar geometry typical for CMA complexes with unsubstituted carbazolate ligand (for instance, CMA1 Figure 1, Table 2) and crystallizes in the orthorhombic space group *P2*_1_2_1_2_1_ from various solvents (THF, DCM, or toluene) (Figure 1 and SI, Figure S6). Geometric parameters are collected in Table 2. The copper analogue **2** can be obtained in two modifications from a DCM/2-methylpentane mixture: as a stable monoclinic *P*2_1_/*c* form which crystallizes at room temperature with two independent molecules in the unit cell and as a metastable triclinic form in space group P-*1*, which crystallizes at −20 °C as a 2-methyl-pentane solvate with three independent molecules in the unit cell. However, the only difference in the structures of these two crystal forms is a small 2–3° tilt of the adamantyl moiety (see SI, Figure S7a). The metastable **2·tricilinic** form (thin plates) converts into stable **2·monoclinic** crystals (large prisms) if left under the mother liquor at room temperature overnight. Compared with the coplanar copper complex (^Ad^CAAC)CuCz, both forms show similar Cu–C_carbene_ but slightly longer (0.01–0.02 Å) Cu–N2 bond lengths.^44^ The twist angle α in **2·triclinic** is ca. 4° larger than in **2·monoclinic** (82.5°, see Figure 1). However, the C1–Cu–N2 angle in the former is 6° smaller than in the latter (168.1° vs 174.8°), with the result that the more distorted **2·tricilinic** form is practically nonemissive, whereas the near-linear **2·monoclinic** is a bright blue emitter in the crystal. We and others have recently exemplified the correlation between such bending (Renner–Teller distortion) and the photoluminescence properties; that is, even a small deviation of ca. 10° from linearity that is found in the excited states of CMA complexes results in severe nonradiative processes.^51,57,58^ In the crystal, CMA molecules have of course only limited freedom to alter their conformation between ground and excited states; thus, the geometry of the excited state will be closely similar to the ground-state geometry obtained from single crystal X-ray diffraction.

**Table 2:** Selected Bond Lengths (Å) and Angles (°) of Copper and Gold Amide Complexes **1**–**4**^a^

	M–C1 (Å)	M–N2 (Å)	C1···N2 (Å)	angle (°) C1–M–N2	torsion angle (°) N1–C1–N2–C28	N2 deviation from M1···C28···C39 (Å)	sum of the angles around N2, (γ) (°)
**CMA1**	1.994(3)	2.027(2)	4.021(3)	178.6(1)	17.1(1)	0.006(3)	359.8(2)
**1**	2.001(5)	2.051(4)	4.052(6)	178.7(2)	20.2(6)	0.030(5)	359.9(2)
2·triclinic	1.891(5)	1.885(3)	3.755(6)	168.1(1)	86.3(5)	0.061(3)	359.4(4)
2·monoclinic	1.882(2)	1.877(2)	3.756(3)	174.8(1)	82.5(2)	0.099(2)	358.1(3)
3·benzene	1.988(3)	2.052(3)	4.040(5)	178.9(1)	63.5(4)	0.338(3)	346.9(3)
3·CH_**2**_**Cl**_**2**_	1.995(3)	2.055(3)	4.047(4)	176.0(1)	69.2(3)	0.320(3)	347.9(3)
**3 desolvated**	1.981(10)	2.037(8)	4.010(13)	172.8(4)	75.2(1)	0.320(10)	348.0(7)
**4**	1.992(4)	2.051(3)	4.036(5)	173.2(1)	25.5(4)	0.389(4)	341.8(4)

a The values for the complexes **2** (triclinic, *P*-1), **2** (monoclinic, *P*2_1_/*c*), **3·benzene**, and solvent free complex **3** (monoclinic, *P*2_1_/*c*) are the average for the independent molecules in the unit cell.

**Figure 2:**
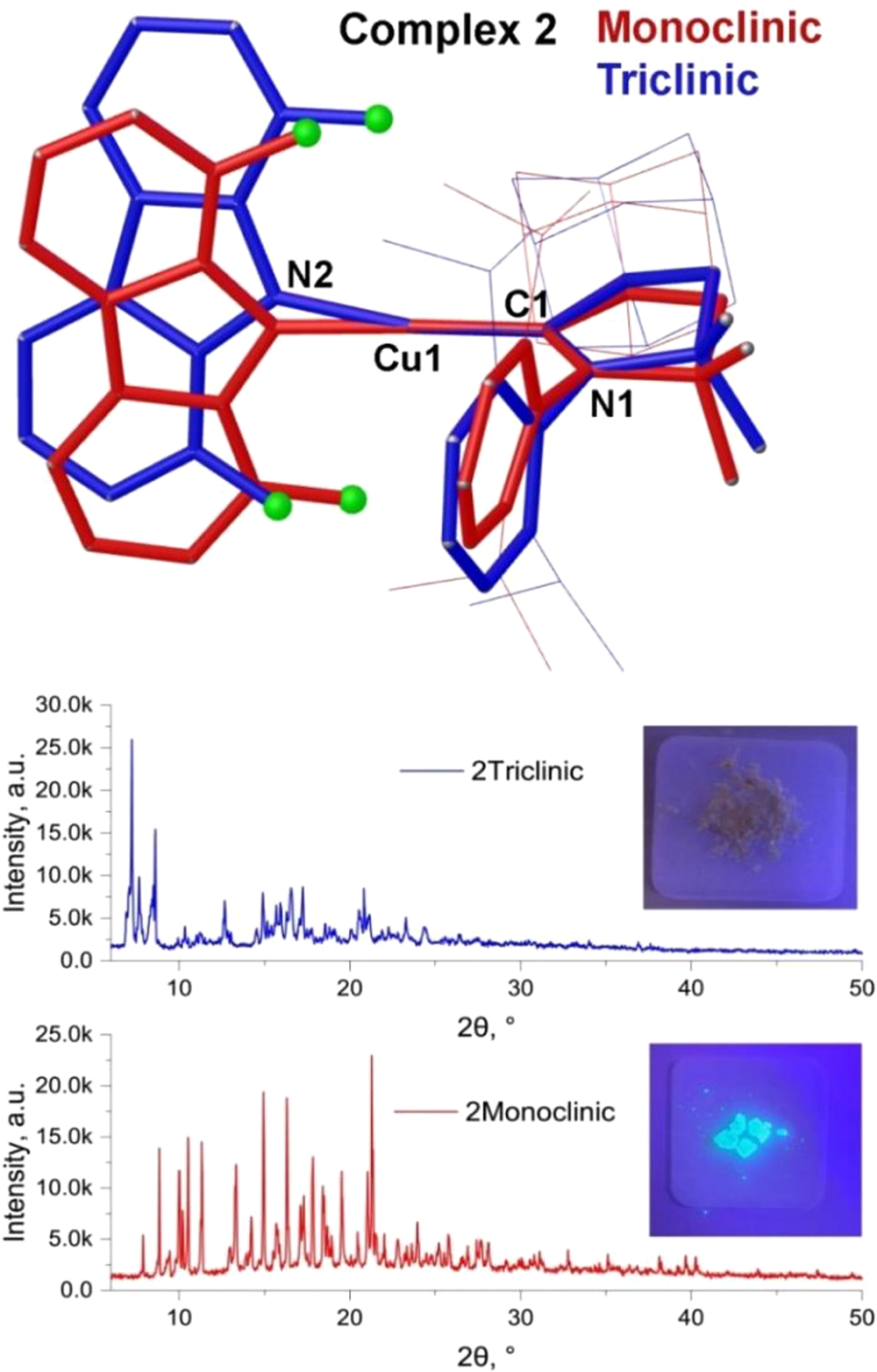
(Top) Superposition of the geometries of different solid-state conformers of **2** determined by single-crystal X-ray diffraction. (Bottom) Corresponding powder diffraction traces and photographs of the materials under irradiation at 360 nm.

Irrespective of these minor structural differences, both forms of **2** exhibit a ground state in which the carbene acceptor and carbazolate donor are mutually orthogonal. This is a reflection of the increased steric congestion in 1,8-disubstituted carbazole complexes of Cu in **2** compared with Au in **1** (covalent radii 1.13, 1.33, and 1.25 Å for two-coordinate Cu, Ag and Au).^59,61^ To confirm the structural uniformity for **2**, we acquired the X-ray powder diffraction data (Figure 2) on a polycrystalline samples for monoclinic and triclinic batches. Indexing of the powder diffraction traces for each form leads to the unit cell parameters identical with those obtained by single crystal X-ray diffraction (Figure 2). We calculated the theoretical conformation energy barrier between nearly coplanar and twisted forms to be as low as 4 kJ·mol^–1^ for complex **1**, whereas it is significantly larger for complex **2** (17 kJ·mol^–1^). This confirms that interconversion between coplanar and orthogonal ligand orientations is energetically highly unfavorable for complex **2**.

**Figure 3:**
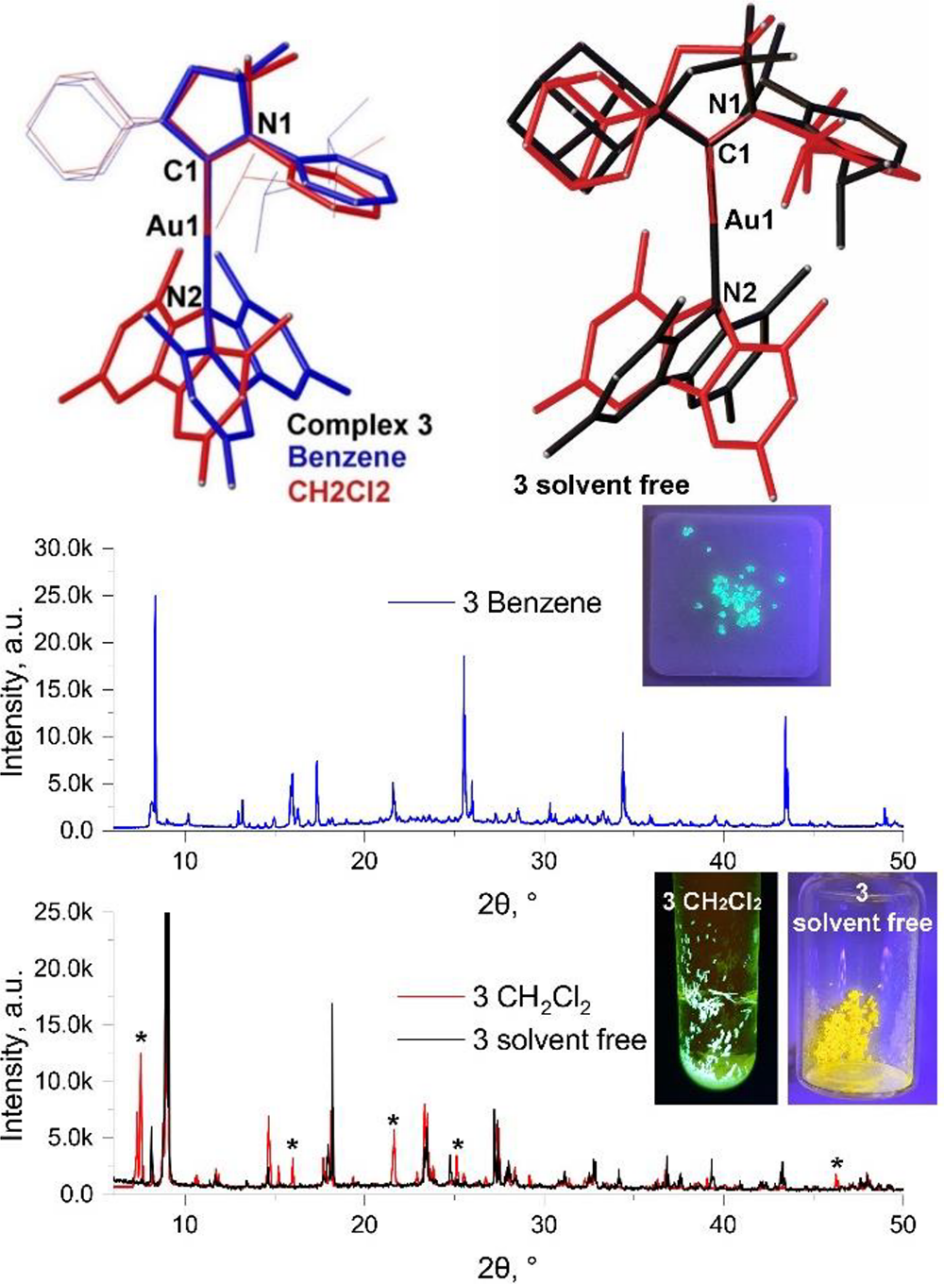
(Top) Superposition (N1, C1, and Au1 atoms) of the geometries of different solid-state conformers of solvated complex **3** and solvent-free complex **3** (independent molecule **A** in black and **B** in red) determined by single-crystal X-ray diffraction. (Bottom) Corresponding powder diffraction traces and photographs of the materials under irradiation at 360 nm for complex **3**, where * indicates a reflection disappearing upon desolvation.

The 1,3,6,8-tetramethylcarbazolate complex **3** crystallizes from benzene and from dichloromethane, respectively, as two solvates, **3·benzene** (two independent molecules due to a tilt of the adamantyl group, see Figure S7) and **3·CH**_**2**_**Cl**_**2**_ (one independent molecule per unit cell). As seen in **2**, the carbene and carbazole ligands in **3** adopt a near-orthogonal orientation. Although these solvates show slightly different torsion angles (the one in **3·CH**_**2**_**Cl**_**2**_ is 5.7° larger than in **3·benzene**), there is an almost opposite orientation of the Cz-ligand, which is 159.5(1)° rotated around the C1–Au1–N2 axis, with the N2 atom as a pivot point, see Figure 3.

The X-ray powder diffraction data (Figure 3) confirm that only one form is present in polycrystalline batches of both **3·benzene** (sky-blue emitter) and **3·CH**_**2**_**Cl**_**2**_ (green emitter). Analysis of the intermolecular contacts shows that the molecules of **3·benzene** are arranged in zigzag “head-to-tail” chains along the crystallographic *a*-axis via weak C–H···π and C–H^δ+^(carbene)···^δ+^H–C(carbazole) interactions between neighboring molecules of **3**. The benzene molecules are aligned along zigzag chains of **3** with similar weak interactions (Figure S7b). Unlike **3·benzene**, the molecules of **3·CH**_**2**_**Cl**_**2**_ form a three-dimensional network via weak C–H···π, C–H^δ+^(carbene)···^δ+^H–C(carbazole),^60^ C1S–H1S ···N2(carbazole) (where S = solvent CH_2_Cl_2_) and C1–H1A(carbene)···Cl interactions between neighboring molecules (Figure S7c). The markedly different packing diagrams for **3·benzene** and **3·CH**_**2**_**Cl**_**2**_ crystals, where the latter shows a weak interaction between CH_2_Cl_2_ and the carbazole N-atom, is likely a reason for the different photophysical properties of these compounds affecting the carbazole-localized HOMO energy level (vide infra). Notably, such contacts between carbazole N-atom and solvents are absent in CMAs with nearly coplanar or twisted molecular designs.

All gold complexes **1**, **3**, and **4** show similar Au–C_carbene_ but 0.03 Å larger Au–N2 bond lengths (Table 2) compared with the coplanar gold complex (^Ad^CAAC)AuCz (CMA1).^44^ This is likely due to the sterics of the substituted carbazole ligand. We analyzed the geometry around the carbazole nitrogen atoms in **1**–**4** to estimate the tilt angles β. The Cz-N atoms in **1** and **2** are trigonal-planar, with a slight tilt for **2** (β = 169.4°). The Cz ligand in **3** is twisted as well as slightly tilted for both solvates **3·benzene** and **3·CH**_**2**_**Cl**_**2**_ (*γ* = 346.9 and 347.9°, respectively). By contrast, in **4** the carbene and Cz ligands are comparable with nearly coplanar CMA (α = 25.5°); however, the sterically encumbered Cz ligand is also strongly tilted (β = 140.7°), and as a consequence, the Cz-N atom shows pronounced pyramidalization (γ = 341.8°).

**3·CH**_**2**_**Cl**_**2**_ loses the CH_2_Cl_2_ solvent molecules upon annealing or drying in air, leading to solvent-free **3** which shows bright-yellow luminescence, see Figure 3. The X-ray powder pattern for **3·CH**_**2**_**Cl**_**2**_ crystals upon desolvation shows only minor changes (see Figure 3, bottom): a few reflections are absent, while major intense reflections have not changed. Therefore, we suggest that the conformation of the desolvated CMA is not changing and in fact is closely similar to that of single crystal structure of the **3·CH**_**2**_**Cl**_**2**_. Nearly identical photophysical parameters between desolvated **3** and neat films of **3** (Table 2) allows us to suggest that the conformation of **3** in a solvent-free sample can be proposed as a major one for the amorphous neat films of **3**. To provide more insights on the molecular structure of the desolvated complex **3**, we performed numerous attempts to grow the single crystal by very slow evaporation of the DCM molecules (two independent molecules in the unit cell, Figure 3 top right and Table 2). The Au–C_carbene_ and Au–N2 bond lengths are shorter by 0.015 Å compared with the solvated complexes of **3**. The Cz ligand in desolvated **3** is nearly twisted with respect to the carbene plane (ca. 10° more than for the solvated **3**), while the tilt angle (146.1°) and pyramidalization parameters of the carbazole N-atom are close similar to the solvated **3**. Such a difference in the twist angle correlates well with the key photophysical parameters discussed below. Analysis of the intermolecular contacts shows that the molecules of desolvated **3** are arranged in a three-dimensional network via weak π(carbazole)···π(carbazole) stacking and C–H (carbene)···π(carbazole) interactions.

Overall, our molecular design results in four different donor-linker-acceptor arrangements: **1** exhibits a structure comparable to the original coplanar complex CMA1,^44^**2** displays an orthogonal arrangement of donor and acceptor ligands, **3** is also twisted but the large steric hindrance of the 1,3,6,8-tetramethylcarbazole introduces a tilt, and finally **4**, which exhibits a strongly tilted but not twisted ground-state geometry (Figure 1). In the following section, the effects of these molecular design variations are studied in detail with regards to the photophysical properties and OLED device performance.

### Photophysical Characterization in the Crystalline State

**Figure 4:**
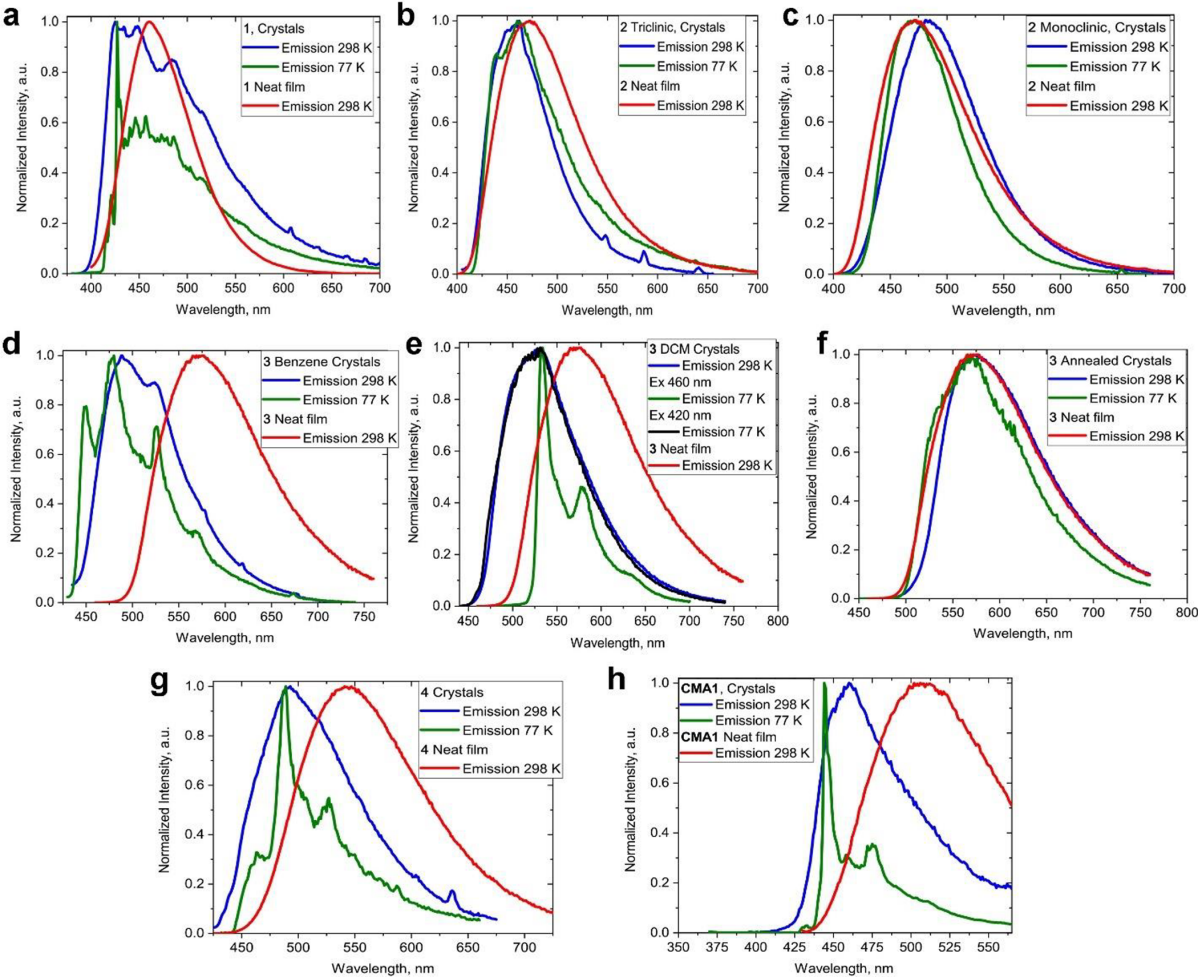
PL spectra at room temperature and at 77 K for crystalline samples and neat films of **1** (a), **2·triclinic** (b); **2·monoclinic** (c); **3·benzene** (d); **3·CH**_**2**_**Cl**_**2**_ (e); solvent free **3** (f), **4** (g) and CMA1 crystals (h).

We first examine the photoluminescence behavior of **1**–**4** in the crystalline state, where thermal motion is suppressed by lattice forces. We then compare these results with solution-processed amorphous thin films with various distribution of the conformers, and finally with the photoluminescence (PL) in solution, where molecular flexibility is unrestricted. Crystals of nearly coplanar **1** at room temperature show a blue, vibronically structured emission at 426 nm, which we ascribe to the luminescence from the locally excited triplet state (^3^LE) of the carbazole ligand. This major peak is 30–60 nm blue-shifted compared with the broad charge transfer (CT) luminescence measured in fluid media or amorphous films (Figure 4a, Table 3).

**Table 3:** Photophysical Properties of Complexes **1**–**4** and **CMA1** in the Crystalline State, as Neat Films, as 20 Weight-% Dopants’ Host–Guest Matrixes, and in Toluene Solution

	λ_em_ (nm)	τ (μs)^c^	Φ (%)^d^	*k*_r_ (10^5^ s^–1^)^e^	*k*_nr_ (10^5^ s^–1^)^e^	CT*/*^3^LE (eV)^f^	Δ*E*(CT–^3^LE)	λ_em_ (nm) at 77K	τ (μs) at 77K
Crystals									
**CMA1**	460	4.3 (31%)	30	0.36	0.84	2.90/2.82	+0.08	444, 458, 475	65.7 (14%)
		10.2 (69%)							277.7 (86%)
**1**	426, 447, 484	12.7 (97.4%)	3	0.02	0.76	3.09/3.00	+0.09	426, 456	130.1 (96.4%); 973.8 (3.6%)
		223.7 (2.5%)							
2·triclinic	461	–	<0.1	–	–	–/2.96	–	439, 461	89.9 (31%); 287.7 (69%)
2·monoclinic	483	22.5	41	0.18	0.26	2.93 (^3^MLCT)	–	469	23
3·benzene	489, 522	4.0	46	1.1	1.3	2.80/2.84	–0.04	448, 479, 525	19.5 (8%); 182.7 (92%)
3·CH_**2**_**Cl**_**2**_	528	0.83	52	6.2	5.7	2.70 (CT)	–	520 (Ex. 400); 533, 578, 630 (Ex 460)	15.1 (75%, ^3^CT); 554.3 (25%, ^3^LE)
						2.37 (^3^LE)			
**3 desolvated**	573	0.48	26	5.4	15.4	2.41/2.48	–0.07	569	8.0 (100%, ^3^CT)
**4**	492	1.95	6	0.3	4.8	2.87/2.79	+0.08	462, 489, 527	18.8 (51%); 161.9 (17%); 1219.9 (32%)
Neat Film^a^									
**CMA1**	506	0.97	85	8.7	1.5	–	–	490	26.3 (46%)
									70.0 (53%)
**1**	476	1.1	24	2	6.9	–	–	430, 453	49.2 (26%); 508.1 (34%); 2744 (40%)
**2**	462	4.5	9	0.2	2	–	–	435, 460	109.8 (27%); 664.7 (45%); 3272 (28%)
**3**	575	0.47	38	8.1	13.2	–	–	543	9.5 (53%); 22.4 (47%)
**4**	535	0.55	33	6.0	12.2	–	–	528	18.8 (66%); 53.2 (32%)
									510.0 (2%)
20% Host–Guest Matrix^b^									
**CMA1**	512	0.92	86	9.3	1.5	–	–	–	–
**1**	456	1.46	60	3.8	3.0	–	–	–	–
**2**	477	3.0	56	2.0	1.3	–	–	–	–
**3**	551	0.50	67	13.4	6.6	–	–	–	–
**4**	520	0.93	86	9.0	1.5	–	–	–	–
Toluene									
**CMA1**	528	1.25	98	7.8	1.6	2.76/2.96	–0.20	426, 438, 453	135.8 (36%), 398.1 (64%)
**1**	487	0.85	100	10.1	0.01	2.94/3.01	–0.07	421, 446, 470	2095
**2**	500	3.8	12	0.3	2.3	2.87/3.00	–0.13	438, 458	36 (56%), 431 (24%)
									5566 (19%)
**3**	591	0.077	5	6.7	123	2.45/2.82	–0.37	491	19.5 (93%), 202 (7%)
**4**	544	0.53	57	10.7	8.2	2.61/2.79	–0.18	452, 485, 512	371 (57%), 867 (43%)

a Films (neat and in host) were prepared by spin-coating from toluene solutions (10 mg/mL) onto a quartz substrate at 90 °C and annealed for 10 min.

b Host–guest films were evaporated from 20 weight-% for **1** in TSPO1 and **2** in o-CBP. Films of **3** and **4** were solution-processed for 40 s at 2000 rpm on a quartz substrate in oCBP from 10 mg/mL chlorobenzene solutions; PVK films for CMA1 were spun from 20 mg/mL chlorobenzene solution for 40 s at 2000 rpm on a quartz substrate.

c In the case of two-component lifetimes, τ average was used: τ_*av*_*=(B*_1_*/(B*_1_*+B*_2_)) τ_1_*+ (B*_2_*/(B*_1_*+B*_2_)) τ_2_, where *B*_1_ and *B*_2_ are the relative amplitudes for τ_1_ and τ_2_.

d Quantum yields determined using an integrating sphere.

e Radiative rate constant *k*_r_ = *Φ/ τ* and nonradiative constant *k*_nr_ = (1 – Φ)*/*τ.

f ^1^CT, ^3^LE, and ^1^LE energy levels based on the onset values of the emission spectra blue edge in MeTHF glasses at 77 K and in toluene solutions at 298 K.

We have observed a similar 46 nm blue-shift for the crystals of the parent CMA1 complex with nearly coplanar geometry (α = 17.1(1)°, Figure 4h) and rationalized this as a combination of two factors, conformational control and a higher polarization energy in the ordered crystalline state.^62,63^ Similar to polycrystalline CMA1, in the crystals of complex **1**, the CT state lies above the carbazole-based locally excited triplet state ^3^LE(Cz) by 0.09 eV (Table 3). This results in a substantial contribution of the ^3^LE phosphorescence to the overall emission process at room temperature, such that the excited-state lifetime increases to a weighted average of 17.2 μs and the PLQY is reduced to 3%, a behavior similar to that of crystalline CMA1 (8.1 μs and 30% PLQY).

Upon cooling, crystals of **1** and of CMA1 show more pronounced ^3^LE phosphorescence, with a 10-fold increase in the excited-state lifetime (Figures 4a,h and S11). This is likely connected with reduced thermal motion (i.e., restricted conformational freedom and arrested spectral relaxation). Evidently, enforcing the coplanar geometry of **1** by a rigid crystalline environment reduces the radiative rates to 2 × 10^3^ s^–1^. This is in startling contrast to the behavior of **1** in toluene solution, where the radiative rate is increased by a factor of 500 (*k*_r_ = 1 × 10^6^ s^–1^, Table 3). It is evident therefore that much more favorable conformations are possible, leading to greatly enhanced radiative rates if relaxation from a coplanar orientation is permitted.^64^

The nearly coplanar complex **1** provides the baseline for comparison with the fully twisted complex **2**. This compound can give two crystal modifications, both with orthogonal donor–acceptor ligand orientation (vide supra). The metastable **2·triclinic** crystals show significant bending distortion in the ground state (Figure 2), resulting in almost nonemissive behavior (Figure 4b, Table 1) at room temperature, although bright blue ^3^LE(Cz) phosphorescence is measured at 77 K (Figures 4b, S11). The stable **2·monoclinic** crystals show a featureless blue luminescence profile, which is 15 nm blue-shifted upon cooling to 77 K (Figure 4c and Table 3).

**Figure 5:**
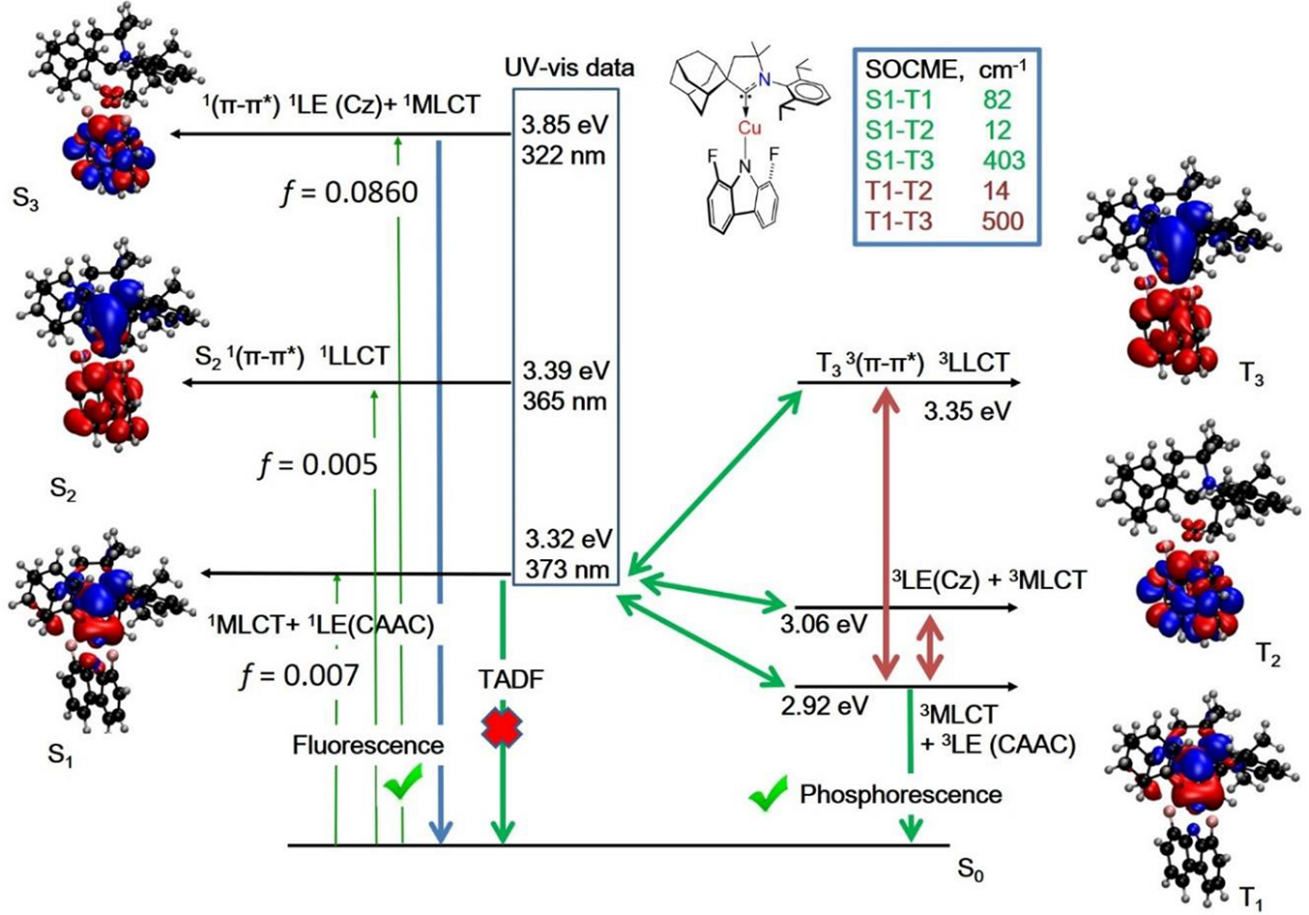
Map of excited states for the singlet (left) and triplet excited states (right) in the ground-state geometry for complex **2** with a difference of the electronic density associated with the electronic states S_1_, S_2_, S_3_, T_1_, T_2_, and T_3_. Theoretically calculated energy levels, oscillator strength coefficients (*f*), and spin–orbit coupling matrix elements (SOCME, cm^–1^) for complex **2**.

The excited-state lifetime of ca. 22 μs is temperature-independent, indicating phosphorescence for **2**. Our theoretical calculations confirm that phosphorescence originates from the lowest-energy ^3^MLCT state with a fully twisted geometry (Figure 5). The fact that both **1** and **2**, as representatives of two extremes in CMA conformations, show phosphorescence, albeit for different reasons, indicates that neither the coplanar nor the fully twisted geometry is ideal for constructing CMA emitters with high radiative rates. It is recently reported that a fully twisted copper analogue of **2** based on the 1,8-dimethyl carbazole also shows^49^ a ^3^MLCT phosphorescence as a major emission mechanism, thus further supporting our results with complex **2**.

In search for CMA compounds with improved conformations, we now consider **3** in the crystalline state. As outlined before, there are substantial donor–acceptor twist angles α of 63 and 69° for **3·benzene** and **3·CH**_**2**_**Cl**_**2**_, respectively, accompanied by a tilt of the carbazole ligand (β ∼ 146°). As-prepared crystals of both **3·benzene** and **3·CH**_**2**_**Cl**_**2**_ give very similar PLQY values of 46 and 52%. The compounds show featureless luminescence profiles ranging from sky-blue **3·benzene** (490 nm, Figure 4d), to green **3·CH**_**2**_**Cl**_**2**_ (528 nm, Figure 4e), and finally to yellow for solvent-free **3** (573 nm, Figure 4f). We assume that such a variety of the luminescence colors is due to solid-state solvatochromism, different packing motifs (zigzag chains vs 3D-network), and intermolecular interactions in the crystal, which is in line with our most recent observation on other CMA emitters.^63^ In the excited state, CMA molecules are destabilized upon photoexcitation because of a change in direction of the dipole moment (which for **3** points toward the Cz group with a magnitude of 8.5 D in the S_1_ state, Table S7), the presence of the nonpolar benzene molecules and confinement in a rigid crystalline matrix restricting intramolecular relaxation. Intramolecular relaxation increases with solvent polarity and in particular for **3·CH**_**2**_**Cl**_**2**_, where a cocrystallized polar DCM molecule interacts directly with the N2-atom of a carbazole, resulting in stabilization of the excited state and a 40 nm red-shift for the CT luminescence. The charge transfer luminescence (2.70 eV) profile of **3·CH**_**2**_**Cl**_**2**_ is independent of the excitation wavelength at room temperature (Figures 4e, S9k), whereas at 77 K we observe a direct excitation of the ^3^LE at 2.37 eV with low energy light starting from 440 nm (Figures 4e, S9l), whereas charge-transfer luminescence is measured when excited with high-energy light in the range from 300 to 420 nm. The absence of the ^3^LE luminescence at room temperature is likely associated with a large energy difference of 0.43 eV between CT and 3LE states, which results in high PLQY values for **3·CH**_**2**_**Cl**_**2**_.

Crystals of **3** show a monoexponential decay of the excited-state lifetime at 298 K, with lifetimes decreasing from 4 μs (**3·benzene**) to 0.86 μs (**3·CH**_**2**_**Cl**_**2**_), down to 0.48 μs for microcrystalline powders of desolvated **3**. Such a decrease in the excited-state lifetime for **3** correlates well with the increase in the twist angle for **3·benzene** (63.5°), **3·CH**_**2**_**Cl**_**2**_ (69.2°) and desolvated **3** (75.2°), as discussed in the X-ray section. Moreover, the PL properties of desolvated microcrystalline and neat film of **3** (as shown in Figures 3 and 4f) are nearly identical, which indicates that the twisted and tilted CMA conformation has remained in the amorphous neat film. Upon cooling to 77 K, the excited-state lifetime increases markedly and shows a biexponential decay for the solvated crystals with benzene and CH_2_Cl_2_ (^3^CT and ^3^LE emission, Figure S11c–e) but monoexponential decay for solvent-free **3**, which we attribute to 100% ^3^CT luminescence. Compared with nearly coplanar **1** or fully twisted **2**, the radiative rates are increased by several orders of magnitude (Table 3).

There remains the question of the relative importance of twisted or tilted CMA conformations. As pointed out above, the donor–acceptor arrangement in **4** (Figure 1) is essentially coplanar (α ca. 25°, similar to **1**) but there is a substantial tilt angle β of ca. 140°. Crystals of **4** show green luminescence with a low PLQY of 6%. This is not least due to the low-lying ^3^LE state, which is 0.08 eV below the CT manifold (Figure 4g, Table 3), similar to **1**. However, the excited-state lifetime of **4** is 6 times shorter than that of **1**, down to 2 μs, which we ascribe to the influence of the carbazole tilt. The data suggest that while in a rigid crystalline environment a carbazolate ligand tilt is useful, a certain degree of twist between donor and acceptor ligands (up to about 70°) may be a more effective means for realizing high radiative rates of up to 6 × 10^5^ s^–1^. On the other hand, a more pronounced ligand tilt correlates with a reduction in excited-state lifetimes.

### Photophysical Characterization of Solid Films

**Figure 6:**
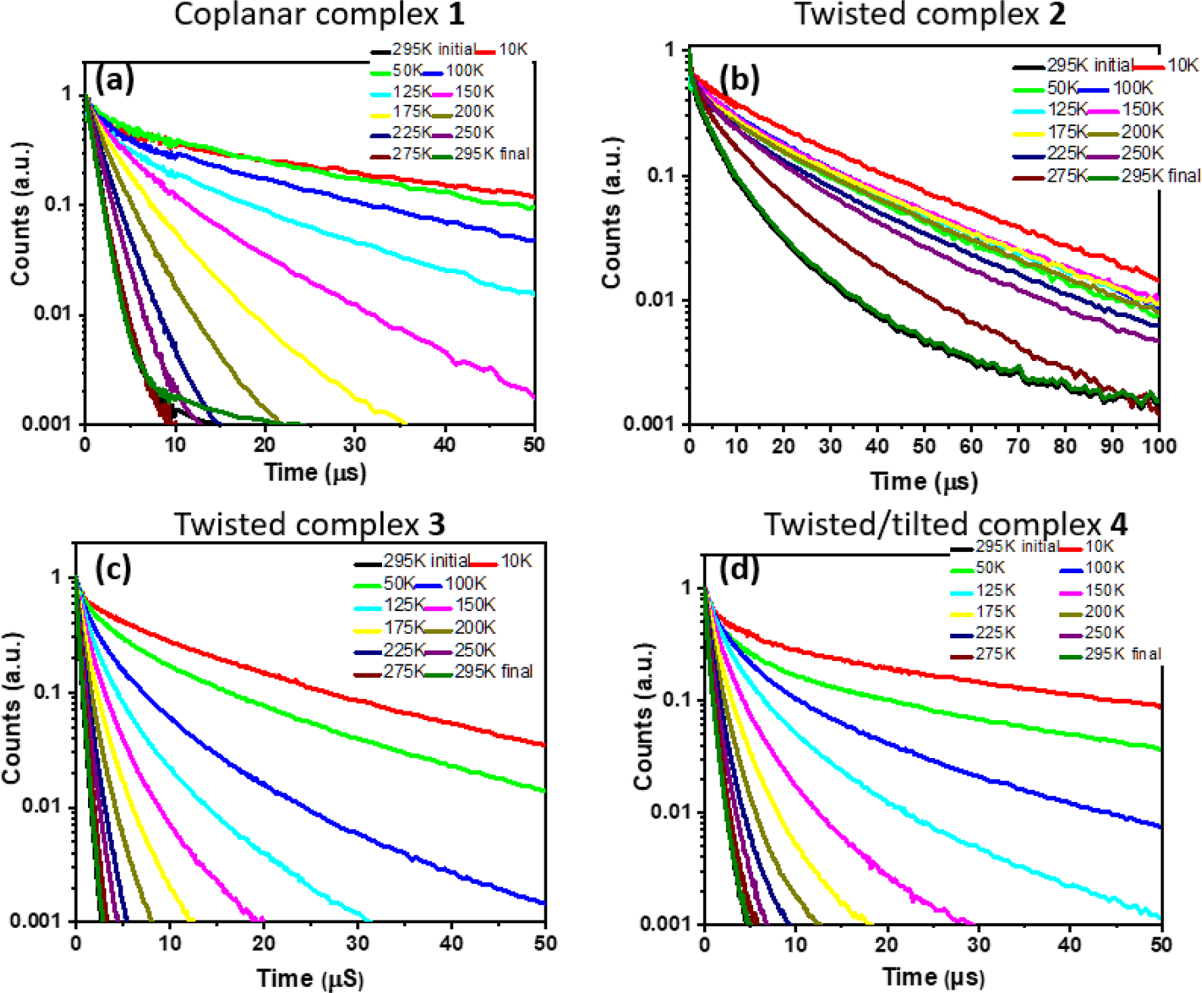
Temperature-dependent time-resolved PL decay for neat solid-state films of complexes **1** (a), **2** (b), **3** (c), and **4** (d). “Initial” data were taken at room temperature before cooling the film to 10 K, and “final” corresponds to data after warming back to room temperature. Films were excited at 400 nm with a laser power of 40 μW. Signals were captured by an ICCD camera.

A comparison of the excited-state lifetimes of twisted/tilted complexes with coplanar/fully twisted CMAs in the amorphous state (as neat films or embedded in a solid host matrix) provide additional insights into the effects of the twist/tilt molecular design in CMA materials. Host–guest films at a dopant level of 20 weight-% were prepared in TSPO1 for **1** and in o-CBP for **2**–**4** (TSPO1 = diphenyl[4-(triphenylsilyl)phenyl]phosphine oxide; o-CBP = 2,2′-bis(N-carbazolyl)-1,1′-biphenyl). Radiative decay rates are extracted from temperature-dependent time-resolved PL decay experiments (Figure 6). All films exhibit featureless luminescence profiles at room temperature. The peak PL of neat films is red-shifted by 15–20 nm compared with host–guest films (Table 3 and Figures 3 and 5). The radiative decay lifetimes for neat films of nearly coplanar CMA1 (0.97 μs), **1** (1 μs) or twisted **2** (4.5 μs) are much longer than those of twisted/tilted **3** (0.47 μs) and tilted **4** (0.55 μs). Host–guest samples of **3** and **4** show a similar trend in lifetime reduction, with τ < 1 μs for both **3** and **4**. This trend is fully consistent with the one observed for crystalline samples; for example, the twisted and tilted **3** in o-CBP as host has the highest radiative rate in the series, *k*_r_ = 1.3 × 10^6^ s^–1^, while tilted **4** is only slightly behind with *k*_r_ = 9.0 × 10^5^ s^–1^, which exceeds the nonradiative decay by almost 1 order of magnitude.

Next we discuss the data on the cryogenic emission integral in a neat films for all complexes (Figure 5). At 300 K, all gold complexes CMA1,^34^**1**, **3**, and **4** show strongly temperature-dependent submicrosecond excited-state lifetimes from CT states (Figure 6a,c,d). These lifetimes increase markedly on cooling to 10 K, to 45.2 μs for **1**, 17.2 μs for **3**, and 44.8 μs for **4**, and are ascribed to ^3^CT emissions (Table 1). The excited-state lifetime for the twisted complex **2** shows only a 5-fold increase upon cooling, from 4.5 μs at 300 K to 26.2 μs at 10K (Figure 6b). Similar to monoclinic crystals **2**, this finding suggests that in this case, TADF is unlikely and phosphorescence is a major emission process even in solution-processed films (Figure s5 and S27). The lowest singlet S_1_ and triplet excited states (T_1_ and T_2_) show a predominantly local character on either the carbazole or carbene ligands. The L(M)LCT transition is ascribed to higher-lying S_2_ and T_3_ states. The S_1_ state is strongly mixed with a manifold of triplet states (T_1_–T_3_), which is promoted by strong spin–orbit coupling of copper.

At 77 K, neat films of **1**, **2**, and **4** show low-energy ^3^CT transitions and higher-energy carbazole-based ^3^LE luminescence, whereas a ^3^LE emission is completely absent for **3**. The local ^3^LE states are long-lived, with lifetimes of 2.7 ms for coplanar **1**, 3.2 ms for twisted **2**, and 0.51 ms for tilt/twisted **4**. The Arrhenius plots of *k*_RISC_ vs 1/*T* allowed us to estimate the reverse intersystem crossing (rISC) activation energies (Δ*E*_a_): 56 meV for **1**, 136 meV for **2**, 42.2 meV for **3**, and 41.8 meV for **4** (Table S1, Figures S12, S15–S17). The rISC activation energies complexes with tilted and/or twisted geometry **3** and **4** are approaching half of those reported for nearly coplanar CMA1 (69 meV),^62^ which is in line with higher HOMO–LUMO overlap integral value for CMA1 (0.36 versus 0.23 for **3**). This clearly indicates the superiority of half-twisted/tilted molecular designs to realize CMA materials with small activation energies Δ*E*_a_ and high radiative rates beyond 10^6^ s^–1^.

### Photophysical Characterization in Solution

**Figure 7:**
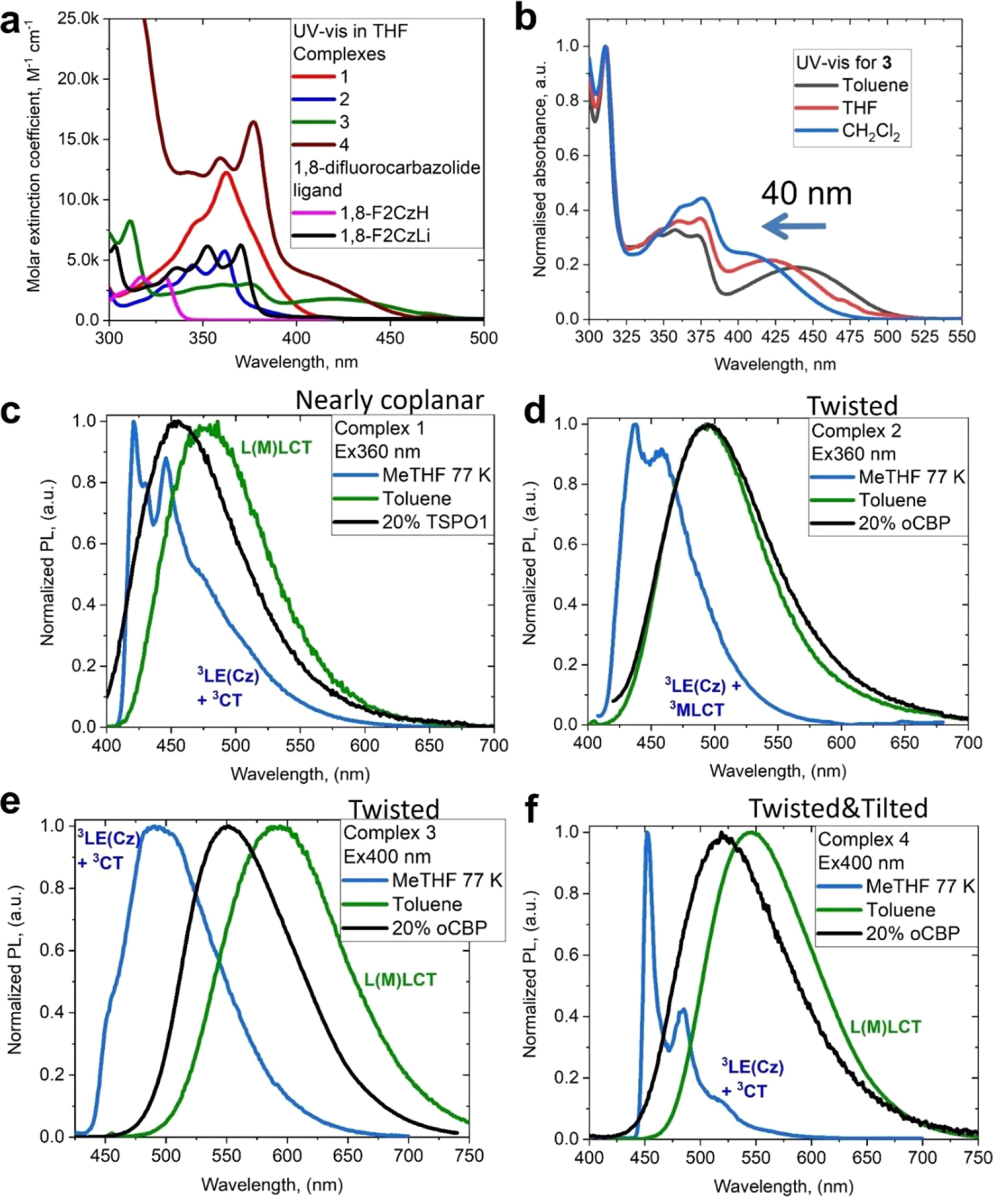
(a) UV–vis spectra of **1**–**4** in THF; (b) UV–vis spectra of **3** in toluene, THF, and CH_2_Cl_2_ solutions showing negative solvatochromism; PL spectra of **1** (c), **2** (d); **3** (e), and **4** (f) in MeTHF at 77 K and in toluene at 298 K and in an o-CBP matrix.

UV–vis and photoluminescence spectra for **1**–**4** are shown in Figure 7; a summary of the photophysical properties recorded in various media is collected in Table 3. The UV–vis absorption spectra of **1** and **2** in THF solution show a strong π–π* intraligand transition (IL) at 362 nm (ε = 12 200 and 5700 M^–1^cm^–1^, respectively). This band has a similar vibronic structure to free 1,8-difluorocarbazole (magenta, Figure 7a) and overlaps with a charge transfer (CT) absorption at ca. 360–370 nm (ε = > 10 000 for **1** and 1200 M^–1^cm^–1^ for **2**). The vibronically resolved π–π* intraligand transition (IL) for free 1,8-difluorocarbazole is 40 nm blue-shifted compared with its lithium salt (black, Figure 7a and S9), which possesses a similar position and profile with the complexes **1** and **2**, thus confirming the π–π* assignment. The lithium salt of the 1,8-difluorocarbazole shows a deep-blue fluorescence at 400 nm (3.32 eV based on the onset on the blue-edge of the luminescence profile, Figure S9j) with poorly resolved vibronic progression at room temperature. The luminescence profile is highly resolved upon cooling to 77 K with a very long excited-state lifetime phosphorescence (over 5 s exceeding the limit of our equipment). The UV–vis absorption spectra for **3** and **4** in THF show broad ^1^L(M)LCT bands at 436 and 402 nm, respectively (ε = 1600 and 4000 M^–1^cm^–1^) (Figure 7 and SI, Figures S28 and S29). Relative to **1** and **2**, the CT bands of **3** and **4** are red-shifted, in line with the decrease of the HOMO–LUMO gap by ca. 0.5 eV identified by cyclic voltammetry (Table 1). The oscillator strengths calculated for the lowest absorption bands are in line with much lower experimental extinction coefficients (ε) and are lowest for twisted complexes (*f* = 0.141 for **1** compared with 0.007 for **2**; *f* = 0.050 for **3** vs 0.131 for **4**) as a consequence of the reduced HOMO/LUMO overlap (Table S7 in SI). All complexes show negative absorption solvatochromism of the charge transfer absorptions and weak positive solvatochromism for CT emissions (Figure S9), consistent with a flipping in the direction of the transition dipole moments (Table S7).^51,53^

Toluene solutions of complexes **1**–**4** show a broad CT emission (Figure 7c-f) with PL maxima for **1** (487 nm), **2** (500 nm), **3** (591 nm) and **4** (544 nm) being red-shifted by 20–60 nm (Table 3) compared with the solid-state PL. We measured the luminescence in 2-methyltetrahydrofuran (MeTHF) at 77K for all complexes to identify the ^3^LE energy level and estimate the energy gap Δ*E*_CT–3LE_. Structured phosphorescence from a local ^3^LE (carbazole) state was observed for complexes **1**, **2** and **4**, whereas **3** shows a ^3^LE emission overlapping with an intense ^3^CT emission (Figure 7e). The Δ*E*_CT–3LE_ values were estimated as −0.07 eV for nearly coplanar **1**, −0.13 eV for twisted **2**, −0.37 eV for twist-tilted **3**, and −0.18 eV for tilted **4**. Our theoretical calculations for the energy difference Δ*E*_1CT-3LE_ (−0.38, −0.33, −0.62, and −0.16 eV for **1**–**4**, respectively, in their S_1_ geometry, see computational details in SI) supports the largest value for complex **3** and are in a reasonable agreement with the experimental data. Also, theory predicts a near-degeneracy between ^1^CT and ^3^CT states for gold complexes in S_1_ geometry (Δ*E*_1CT-3CT_ is in the range from +0.01 to +0.05 eV), whereas there is a large energy gap (Δ*E*_1CT-3CT_ is +0.30 eV) calculated for the copper complex **2**.

Complex **1** has an excited-state lifetime of 0.85 μs and achieves unity quantum yield at room temperature, which is superior to that of the parent complex (^Ad^CAAC)AuCz (CMA1, τ = 1.25 μs and PLQY 98%).^44^ Such difference in excited-state lifetime for complexes with nearly coplanar geometry is in line with smaller HOMO–LUMO overlap for complex **1** (0.27) compared with parent CMA1 (0.36). In contrast, **2** exhibits a significantly longer lifetime, consistent with the reduced radiative rate arising from the smaller oscillator strength due to the twisted structure of **2**. This reduced radiative rate is also responsible for the significant reduction in PLQY for **2** compared with **1**.

The twist-tilted complex **3** is an orange-red emitter in toluene (λ_em_ = 590 nm), which has the lowest PLQY value in toluene (5%) while showing the largest nonradiative rate.

**Scheme 1:**
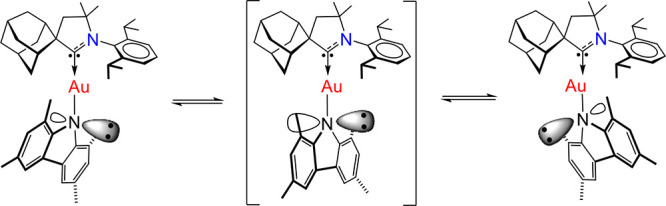
Inversion of the Pyramidal Configuration at the Nitrogen Atom of the Carbazole for Complex **3** in Solution

By contrast, the radiative rate of **3** is comparable to **1**, which agrees with the reasonably high oscillator strength of **3** (*f* = 0.050, Figure S28). Compared with **3**, toluene solutions of the tilted complex **4** show a much higher PLQY (57%). The conjugated structure in complex **4** increases the HOMO–LUMO overlap (0.23 for **3** to 0.31 for **4**) and consequently the S_1_ oscillator strength (*f* = 0.131). We suggest that lower PLQY values for the solutions of the tilted complexes **3** and **4** are due to an additional nonradiative process associated with the inversion of the pyramidal configuration at the nitrogen atom of the carbazole as shown on Scheme 1. This is more pronounced for the twisted **3** as evidenced by very short excited-state lifetime in toluene (77 ns) and much less for nearly coplanar, tilted **4**. In the solid state, such a nonradiative process is impossible, eliminating this decay route and markedly increasing the PL efficiency, as shown by the high PLQY values in o-CBP host films of >50% for twisted **2** and **3** and 86% for tilted **4**.

### OLED Devices

To demonstrate the practical potential of the proposed molecular designs, we fabricated two sets of OLED devices, using thermal vapor deposition under high vacuum (10^–7^ Torr) for **1** (coplanar) and **2** (twisted) and solution-processing methods for **3** and **4**.

**Figure 8:**
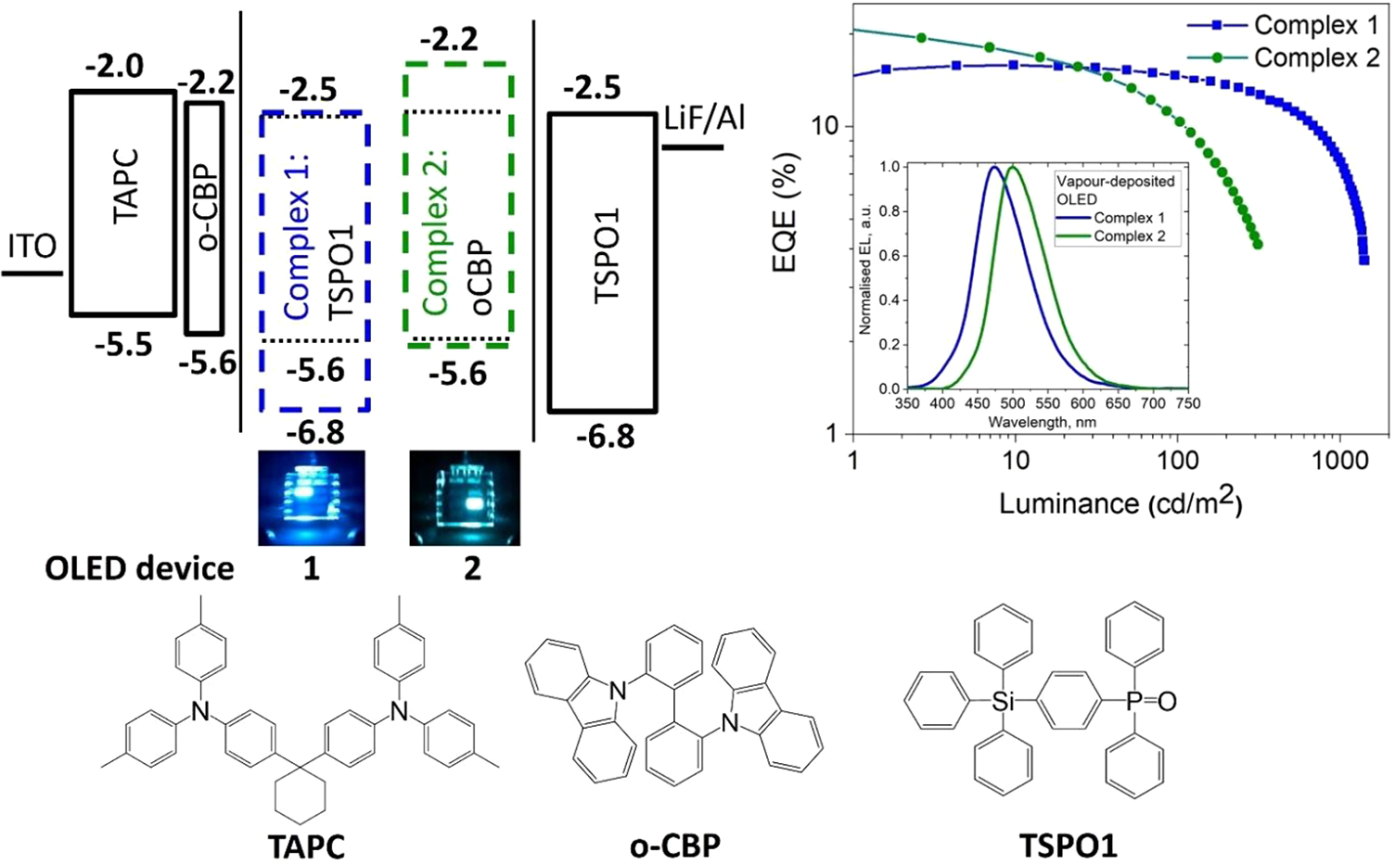
Vapor-deposited OLED device architectures for **1** and **2** (left); EQE versus luminance and EL spectra of OLEDs based on **1** and **2** (right), and structures of host materials (bottom). The driving voltage for the OLEDs in the photographs is set to 6 V.

Several device architectures were employed (Figures 8, S18–S24) to identify the optimal OLED stack. For **1** and **2**, the following structure shows the best performance: ITO/TAPC (40 nm)/o-CBP (5 nm)/emissive layer (EML) (30 nm)/TSPO1 (40 nm)/LiF (0.8 nm)/Al (100 nm) (Figure 8). The TSPO1 host has a high triplet energy of 3.4 eV, which gives an acceptable energy alignment with the triplet energy of the nearly coplanar dopant **1** (3.01 eV, Table 1). Champion devices with complex **1** show sky-blue electroluminescence (EL, λ_max_ = 480 nm) with a maximum external quantum efficiency (EQE) of 11.3% and small roll-off at a practical brightness of 100 cd m^–2^. For the twisted complex **2**, the low-polarity host o-CBP provided the best OLED devices, with an EL peak at 500 nm and an EQE of 10.6% at a practical brightness of 100 cd m^–2^. We optimized the OLED device stack to achieve a charge balance and almost negligible current leakage (for current density–voltage and luminance–voltage characteristics of the evaporated devices, see Figures S18–S21). All device stacks show high EQEs at low current density, together with low turn-on voltages (Table 4).

**Table 4:** Performance Data of Vapor-Deposited and Solution-Processed OLEDs

		EQE (%)				
dopant	*V*_ON_ (V)^a^	max	100 cd m^–2^	1000 cd m^–2^	EL (nm)	CIE (*x*,*y*)^b^
Vapor-Deposited						
TSPO1:**1**	3.2	11.3	10.5	5.9	445	(0.17, 0.26)
o-CBP:**2**	3.2	23.2	10.6	–	500	(0.22, 0.46)
Solution-Processed						
o-CBP:**3**	4.3	14.1	14.1	12.5	540	(0.36, 0.56)
o-CBP:**4**	3.9	19.1	19.0	16.7	525	(0.28, 0.57)

a Values at brightness >1 cd m^–2^;

b Commission Internationale de l’Éclairage (CIE) color coordinates

**Figure 9:**
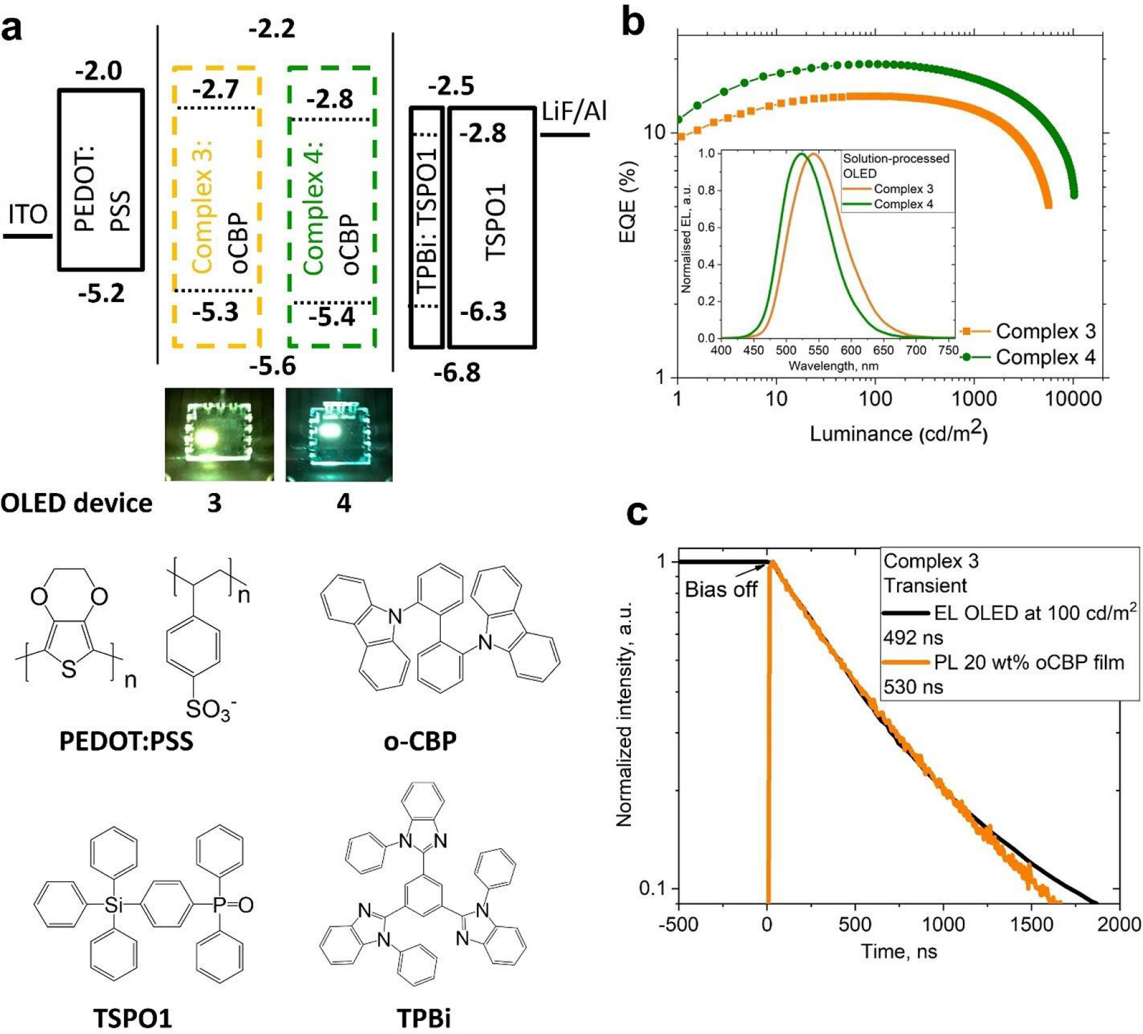
(a) Solution-processed OLED device architectures based on **3** and **4**; (b) EQE versus luminance of champion OLEDs based on **3** and **4** with an inset showing the EL spectra of these OLEDs; (c) Time-resolved photo- (PL) and electroluminescence (EL) decays for complex **3**. The driving voltage for the OLEDs in the photographs is set to 6 V.

Because of the lower volatility of **3** and **4**, OLEDs were fabricated using solution-processing methods (Figure 9). The device architecture employed was ITO/PEDOT:PSS (40 nm)/EML(40 nm)/10 wt % TPBi:TSPO1 (10 nm)/TSPO1 (40 nm)/LiF (0.8 nm)/Al (100 nm) (Figure 9 and Figures S22–S24). Fabrication of solution-processed devices with a small molecule host is a challenging target. We found that a thin layer of TSPO1 doped with 10% of TPBi improves the charge balance in all device architectures based on **3** and **4**. Using this method, highly efficient OLEDs were obtained, with peak EQEs of 14.1% (λ_max_ 540 nm) and 19.0% (λ_max_ 525 nm) for **3** and **4**, respectively (Figure 9). Compared with the vacuum-deposited OLEDs based on **1** and **2**, the roll-off effect was significantly reduced (to only 2–3%), while high efficiency was maintained, with EQEs of 14.1% and 19.0% for **3** and **4**, respectively, measured at a brightness of 100 cd m^–2^.

We also measured the transient-EL (electroluminescence) characteristic of the OLED device for the complex **3**, having the most promising molecular design, to compare the transient PL for 20 wt % oCBP film prepared in the same way and shown on Figure 9c. The PL excited-state lifetime for complex **3** (0.50 μs) is similar to transient electroluminescence EL (0.49 μs) in the OLED device held at a practical brightness of 100 cd m^–2^ (or current density of 0.08 mA cm^–2^). Such similarity between PL and EL-transient kinetics indicates that molecular design concepts for new CMA emitters can be successfully translated into the OLED device.

## Conclusion

**Figure 10:**
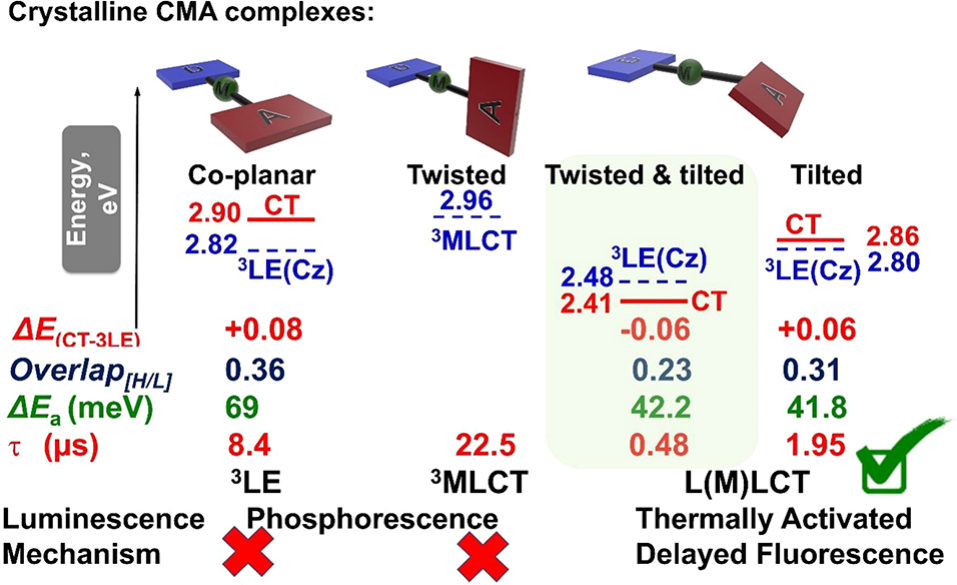
General overview of the key emission mechanisms for complexes **1**–**4** in a crystalline state depending on their conformation with the key electronic properties (energy gap between charge transfer and triplet local excited states Δ*E*_CT-3LE_, RISC activation energy, Δ*E*_a_, and excited-state lifetime, τ) and calculated overlap integral between HOMO and LUMO (Overlap_[H/L]_).

We have successfully demonstrated that a molecular design strategy based on twisted and tilted donor–acceptor orientation is able to realize highly efficient CMA materials beyond the conventional nearly coplanar or twisted approach applied in purely organic materials (Figure 10). Our findings disclose the role of sterically bulky ligands and the size of the metal atom as effective tools to control the geometry of the CMA materials in the solid state and the photophysical properties and most notably the excited-state lifetimes. The structure of the CMA emitters in fluid media is different than that observed in the solid state because of constant rotation and inversion of the pyramidal geometry of the N atom in line with the small rotational barriers calculated for the crystallographically determined conformations of the emissive complexes. Although the metal atoms make only small contributions to the HOMO and LUMO levels, their high spin–orbit coupling coefficients lead to fast intersystem crossing and hence short luminescence lifetimes at the expense of competing phosphorescence processes, particularly in the gold complexes. We find that crystalline CMA complexes with nearly coplanar **1** and twisted geometry demonstrate phosphorescence originating from the ligand for **1** or ^3^MLCT state for twisted complex **2**. Both fully or partially twisted emitters **3** and **4** suffer from significant nonradiative losses in fluid media; however, these are suppressed in rigid environments, and high PLQY values are observed. We demonstrate that a combination of tilted and partially twisted molecular designs can reduce the excited-state lifetimes down to 470 ns, while radiative rates increase to 1.3 × 10^6^ s^–1^ in host–guest systems. These short excited-state lifetimes place gold-based CMA emitters on par with champion silver-based CMAs.^48,50^ In the tilted and tilt-twisted complexes **3** and **4**, the rISC activation energies (Δ*E*_a_) are reduced to values as low as 42.2 meV, which is nearly half the value found for nearly coplanar CMA1 (69 meV, Figure 10).^34,62^ Crystallization of twisted and tilted complex **3** from different solvents enabled us to establish a correlation between the carbene and carbazole ligands twist angle and excited-state lifetime: the greater the twist angle the shorter the excited-state lifetime. This correlation parallels well with simultaneous decrease of the overlap integral between HOMO and LUMO. As a demonstration of the effects of the new molecular designs we report new vapor-deposited and solution-processed OLED device stacks which realize EQE values of up to 19% at a practical brightness of 100 cd m^–2^. Solution-processed OLED devices with twisted and tilted complexes **3** and **4** demonstrate small roll-off values of up to 2% at a brightness of 1000 cd m^–2^. Overall, this study demonstrates that the structural versatility available for organometallic complexes based on CMAs are an effective platform to control the mutual donor–acceptor orientation and provides a facile means for optimizing excited-state lifetimes.

## Experimental Section

### General Considerations

Unless stated otherwise, all reactions were carried out in air. Solvents were distilled and dried as required. Sodium *tert*-butoxide, 2-bromo-1-chloro-3-fluorobenzene, and 2-fluoroaniline were purchased from FluoroChem. SPhos Pd G2 was purchased from Sigma-Aldrich. 5,12-Dihydro-5-phenyl-indolo[3,2-*a*]-9*H*-carbazole was purchased from TCI. All chemical were used as received. The carbene ligand (^Ad^L),^65−67^*N*-(2-chloro-4-(trifluoromethyl)phenyl)acetamide and 1,3,6,8-tetramethyl-9*H*-carbazole,^68^ and complexes (^Ad^L)MCl (M = Cu and Au)^69,70^ were obtained according to literature procedures. In the present work, difluorocarbazole ligand is synthesized in a way that unambiguously rules out the presence of any isomeric impurity from the commercial carbazole to avoid misleading photophysical data.^71^^1^H and ^13^C{^1^H} NMR spectra were recorded using a Bruker Avance DPX-300 MHz NMR spectrometer. ^1^H NMR spectra (300.13 MHz) and ^13^C{^1^H} (75.47 MHz) were referenced to CD_2_Cl_2_ at δ 5.32 (^13^C, δ 53.84), C_6_D_6_ at δ 7.16 (^13^C, δ 128.4), CDCl_3_ at δ 7.26 (δ ^13^C 77.16) ppm. All electrochemical experiments were performed using an Autolab PGSTAT 302N computer-controlled potentiostat. Cyclic voltammetry (CV) was performed using a three-electrode configuration consisting of either a glassy carbon macrodisk working electrode (GCE) (diameter of 3 mm; BASi, Indiana, U.S.A.) combined with a Pt wire counter electrode (99.99%; GoodFellow, Cambridge, U.K.) and an Ag wire pseudoreference electrode (99.99%; GoodFellow, Cambridge, U.K.). The GCE was polished between experiments using alumina slurry (0.3 μm), rinsed in distilled water, and subjected to a brief sonication to remove any adhering alumina microparticles. The metal electrodes were then dried in an oven at 100 °C to remove residual traces of water, and the GCE was left to air-dry and residual traces of water were removed under vacuum. The Ag wire pseudoreference electrodes were calibrated to the ferrocene/ferrocenium couple in THF at the end of each run to allow for any drift in potential, following IUPAC recommendations.^72^ All electrochemical measurements were performed at ambient temperatures under an inert Ar atmosphere in THF containing the complex under study (0.14 mM) and supporting electrolyte [*n*-Bu_4_N][PF_6_] (0.13 mM). Data were recorded with Autolab NOVA software (v. 1.11). Elemental analyses were performed by London Metropolitan University. UV–visible absorption spectra were recorded using a PerkinElmer Lambda 35 UV–vis spectrometer. Mass spectrometry data were obtained using APCI(ASAP) (Atmospheric Solids Analysis Probe) at the National Mass Spectrometry Facility at Swansea University.

### Synthesis of 1,8-Difluorocarbazole

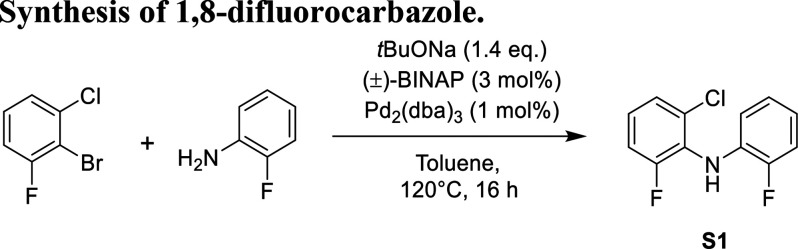



#### Synthesis of 2-Chloro-6-fluoro-*N*-(2-fluorophenyl)aniline (**S1**)

Pd_2_(dba)_3_ (1 mol %, 0.15 mmol, 137 mg) and *rac*-BINAP (3 mol %, 0.45 mmol, 280 mg) were mixed in toluene (30 mL) for 10 min. 2-Bromo-1-chloro-3-fluorobenzene (1 eq., 15 mmol, 3.14 g), *t*BuONa (1.4 eq., 21 mmol, 2.02 g) and 2-fluoroaniline (1 eq., 15 mmol, 1.45 mL) were successively added, and the atmosphere was flushed with argon. The vessel was sealed, and the mixture was heated at 120 °C for 16 h. The reaction was cooled to r.t., Et_2_O (90 mL) was added, and the mixture was filtered through Celite. The filtrate was diluted with Et_2_O (200 mL), washed with water and brine, and dried with MgSO_4_. The solvent was evaporated, and the residue was purified by silica column chromatography (100% PE) to afford the product as a colorless oil (72%, 2.58 g).

^1^H NMR (300 MHz, CDCl_3_): δ 7.29–7.24 (m, 1H, overlapping with CDCl_3_ residual signal), 7.14–7.04 (m, 3H), 7.01–6.93 (m, 1H), 6.91–6.77 (m, 1H), 6.69–6.57 (m, 1H), 5.85–5.62 (bs, NH). ^13^C{^1^H} NMR (75 MHz, CDCl_3_) δ 157.06 (d, *J* = 250.2 Hz, C–F), 152.6 (d, *J* = 241.3 Hz, C–F), 131.9 (dd, *J* = 10.9, 1.3 Hz, C–N), 130.0 (d, *J* = 3.7 Hz, C–Cl), 127.1 (d, *J* = 14.2 Hz, C–N), 125.7 (d, *J* = 3.4 Hz, CH), 124.9 (d, *J* = 8.8 Hz, CH), 124.2 (d, *J* = 3.7 Hz, CH), 120.6 (d, *J* = 7.1 Hz, CH), 116.0 (dd, *J* = 3.7, 2.3 Hz, CH), 115.4 (d, *J* = 6.1 Hz, CH), 115.1 (d, *J* = 4.2 Hz, CH). ^19^F NMR (282 MHz, CDCl_3_) δ −116.2 (m), −133.5 (m). Anal. Calcd for C_12_H_8_ClF_2_N (239.65): C, 60.14; H, 3.36; N, 5.84. Found: C, 60.30; H, 3.13; N, 5.69. C_12_H_8_ClF_2_N theoretical [M+H^+^] = 240.0392, HRMS (APCI(ASAP)) = 240.0392.
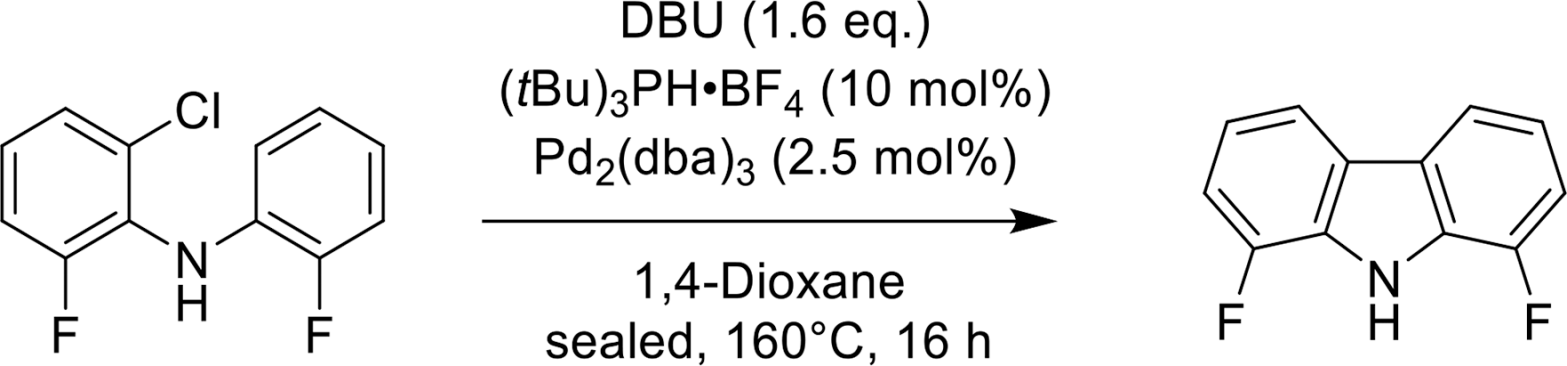


#### Synthesis of 1,8-Difluoro-9*H*-carbazole (**S2**)

In a J. Young valve Schlenk tube, Pd_2_(dba)_3_ (2.5 mol %, 0.20 mmol, 181 mg) and [(*t*Bu)_3_PH]BF_4_ (10 mol %, 0.79 mmol, 229 mg) were mixed in 1,4-dioxane (30 mL) for 10 min. 1,8-Diazabicyclo(5.4.0)undec-7-ene (“DBU”, 1.6 eq., 12.7 mmol, 1.90 mL) and **2-chloro-6-fluoro-*****N*****-(2-fluorophenyl)aniline (S1)** (1 eq., 7.93 mmol, 1.90 g) dissolved in 1,4-dioxane (10 mL) were successively added. The vessel was sealed, and the mixture was stirred at 160 °C for 16 h. The reaction was cooled to r.t., and the volatiles were evaporated. The residue was taken back in AcOEt (250 mL) and washed with H_2_O and brine and dried with MgSO_4_. The solvent was evaporated, and the residue was purified by silica column chromatography (100% PE) to afford the product as a white solid (93%, 1.50 g).

^1^H NMR (300 MHz, CDCl_3_): δ = 8.40–8.28 (bs, 1H, NH), 7.86–7.80 (m, 2H, CH^4^ Cz), 7.23–7.15 (m, 4H, CH^2^ and CH^3^ Cz overlapping). ^13^C{^1^H} NMR (75 MHz, CDCl_3_): δ 149.4 (d, *J* = 243.7 Hz, C–F), 127.8 (dd, *J* = 13.4, 1.0 Hz, C–NH), 126.8 (dd, *J* = 4.9, 2.6 Hz, NHC–C–CH^4^), 120.4 (d, *J* = 5.9 Hz, CH^3^), 116.4 (d, *J* = 3.7 Hz, CH^4^), 111.8 (d, *J* = 16.1 Hz, CH^2^). ^19^F NMR (282 MHz, CDCl_3_): δ −134.6. Anal. Calcd for C_12_H_7_F_2_N (203.19): C, 70.93; H, 3.47; N, 6.89. Found: C, 70.60; H, 3.53; N, 6.76. C_12_H_7_F_2_N theoretical [M+H^+^] = 204.0625, HRMS (APCI(ASAP)) = 204.0621.

#### Synthesis of (^Ad^CAAC)Au(1,8-Difluorocarbazole) (**1**)

In a Schlenk tube, (^Ad^CAAC)AuCl (2.44 g, 4 mmol), 1,8-difluoro-9*H*-carbazole (0.813 g, 4 mmol), and ^*t*^BuOK (0.449 g, 4 mmol) in THF (50 mL) were stirred for 6 h. The mixture was filtered through Celite. The filtrate was concentrated and washed with hexane to afford the product as a white solid. Yield: 2.23 g (2.9 mmol, 72%).

^1^H NMR (300 MHz, CD_2_Cl_2_): δ 7.75–7.68 (m, 2H, CH^4^ Cz), 7.52 (t, *J* = 7.8 Hz, 1H, *p*-CH Dipp), 7.33 (d, *J* = 7.8 Hz, 2H, *m*-CH Dipp), 6.94–6.76 (m, 4H, CH^2^ and CH^3^ Cz overlapping), 4.29 (d, *J* = 13.1 Hz, 2H, CH_2_ adamantyl), 2.86 (sept, *J* = 6.8 Hz, 2H, CH *i*Pr), 2.40 (s, 2H, CH_2_ CAAC), 2.36–1.80 (m, 12H, adamantyl), 1.34 (s, 6H, C(CH_3_)_2_ CAAC overlapping with CH_3_*i*Pr), 1.32 (d, *J* = 6.8 Hz, 12H, CH_3_*i*Pr overlapping with C(CH_3_)_2_ CAAC). ^13^C{^1^H} NMR (75 MHz, CD_2_Cl_2_): δ 241.0 (s, C_carbene_ CAAC), 151.2 (d, *J* = 247.1 Hz, C–F), 145.0 (s, *o*-C Dipp), 138.1 (d, *J* = 9.7 Hz, C–N Cz), 135.6 (s, *ipso*-C Dipp), 129.8 (s, *p*-CH Dipp), 128.6 (dd, *J* = 6.0, 3.2 Hz, N–C–C–CH^4^ Cz), 125.4 (s, *m*-CH Dipp), 116.5 (d, *J* = 6.2 Hz, CH^3^ Cz), 115.4 (d, *J* = 3.5 Hz, CH^4^ Cz), 109.6 (d, *J* = 18.6 Hz, CH^2^ Cz), 77.4 (s, C(CH_3_)_2_ CAAC), 65.0 (s, C–C_carbene_ CAAC), 49.0 (s,CH_2_ CAAC), 39.5 (s, CH_2_ adamantyl), 37.5 (s, CH adamantyl), 35.4 (CH_2_ adamantyl), 35.0 (s, CH_2_ adamantyl), 29.5 (s, CH *i*Pr), 29.4 (s, C(CH_3_)_2_ CAAC), 28.3 (CH adamantyl), 27.7 (s, CH adamantyl), 26.5 (s, CH_3_*i*Pr), 23.2 (s, CH_3_*i*Pr). ^19^F{^1^H} NMR (282 MHz, CD_2_Cl_2_): δ −128.7. Anal. Calcd for C_39_H_45_AuF_2_N_2_ (776.77): C, 60.31; H, 5.84; N, 3.61. Found: C, 60.15; H, 5.93; N, 3.59. C_39_H_45_AuF_2_N_2_ theoretical [M+H^+^] = 777.3295, HRMS (APCI(ASAP)) = 777.3301.

#### Synthesis of (^Ad^CAAC)Cu(1,8-Difluorocarbazole) (**2**)

Following the procedure described for **1**, the complex was made from (^Ad^CAAC)CuCl (2.38 g, 5 mmol), 1,8-difluoro-9*H*-carbazole (1.02 g, 5 mmol), and *t*BuOK (0.561 g, 5 mmol) as a white solid. Yield: 2.79 g (4.4 mmol, 88%).

^1^H NMR (300 MHz, CD_2_Cl_2_): δ 7.74 (pseudo d, *J* = 7.1 Hz; 2H, CH^4^ Cz), 7.35 (t, *J* = 7.7 Hz, 1H, *p*-CH Dipp), 7.23 (d, *J* = 7.7 Hz, 2H, *m*-CH Dipp), 6.98–6.72 (m, 4H, CH^2^ and CH^3^ Cz overlapping), 3.83 (d, *J* = 13.0 Hz, 2H, CH_2_ adamantyl), 2.99 (sept, *J* = 6.7 Hz, 2H, CH *i*Pr), 2.32 (s, 2H, CH_2_ CAAC), 2.14–1.71 (m, 12H, adamantyl), 1.38 (s, 6H, C(CH_3_)_2_ CAAC), 1.32 (d, *J* = 6.7 Hz, 6H, CH_3_*i*Pr), 1.22 (d, *J* = 6.7 Hz, 6H, CH_3_*i*Pr). ^13^C{^1^H} NMR (75 MHz, CD_2_Cl_2_) δ 255.1 (s, C Carbene), 151.7 (d, *J* = 243.4 Hz, C–F), 145.5 (s,*o*-CDipp), 138.2 (d, *J* = 10.9 Hz, N–C Cz), 136.0 (s, *ipso*-C Dipp), 129.6 (s, *p*-CH Dipp), 128.4 (dd, *J* = 6.7, 3.3 Hz, N–C–C–CH^4^ Cz), 125.1 (s, *m*-CH Dipp), 115.8 (d, *J* = 6.4 Hz, CH^3^ Cz overlapping with CH^4^ Cz), 115.7, (d, *J* = 3.0 Hz, CH^4^ Cz overlapping with CH^3^ Cz), 108.9 (d, *J* = 17.9 Hz, CH^2^ Cz), 79.1 (s, C(CH_3_)_2_ CAAC), 66.1 (s, C–C: CAAC), 48.3 (s, CH_2_, CAAC), 39.0 (s, CH_2_ adamantyl), 37.8 (s, CH adamantyl), 36.1 (s, CH_2_ adamantyl), 34.8 (s, CH_2_ adamantyl), 29.8 (s, C(CH_3_)_2_), 29.5 (s, CH iPr), 28.4 (s, CH adamantyl), 27.7 (s, CH adamantyl), 26.8 (CH_3_ iPr), 23.1 (s, CH_3_*i*Pr). ^19^F NMR (282 MHz, CD_2_Cl_2_): δ −129.1. Anal. Calcd for C_39_H_45_CuF_2_N_2_ (643.35): C, 72.81; H, 7.05; N, 4.35. Found: C, 72.66; H, 7.18; N, 4.25. C_39_H_45_CuF_2_N_2_ theoretical [M+H^+^] = 643.2925, HRMS (APCI(ASAP)) = 643.2924.

#### Synthesis of (^Ad^CAAC)Au(1,3,6,8-Tetrametylcarbazole) (**3**)

Following the procedure described for **1**, the complex was made from (^Ad^CAAC)AuCl (2.44 g, 4 mmol), 1,3,6,8-tetramethyl-9*H*-carbazole (0.893 g, 4 mmol), and ^*t*^BuOK (0.449 g, 4 mmol) as a yellow solid. Yield: 2.64 g (3.4 mmol, 83%).

^1^H NMR (300 MHz, CD_2_Cl_2_): δ 7.52 (s, 2H, CH^4^ Cz), 7.44 (t, *J* = 7.8 Hz, 1H, *p*-CH Dipp), 7.29 (d, *J* = 7.8 Hz, 2H, *m*-CH Dipp), 6.77 (s, 2H, CH^2^ Cz), 3.79 (d, *J* = 12.1 Hz, 2H, CH_2_ adamantyl), 2.93 (sept, *J* = 6.6 Hz, 2H, CH *i*Pr), 2.51 (s, 6H, 1,8-Me_2_, Cz), 2.40 (6H, 3,6-Me_2_, Cz) overlapping with 2.41–1.74 (m, 14H, CH and CH_2_ adamantyl), 1.40 (s, 6H, C(CH_3_)_2_ CAAC), 1.34 (d, *J* = 6.6 Hz, 6H, CH_3_*i*Pr) overlapping with 1.30 (d, *J* = 6.6 Hz, 6H, CH_3_*i*Pr). ^13^C{^1^H} NMR (75 MHz, CD_2_Cl_2_): δ 244.6 (C_carbene_ CAAC), 148.8 (*ipso*-C–N, Cz), 145.2 (*o*-C Dipp), 136.1 (*ipso*-C Dipp), 129.9 (*p*-CH Dipp), 127.0 (CH^2^, Cz), 125.8 (*ipso*-C–C, Cz), 125.6 (*m*-CH Dipp), 125.1 (*ipso*-C^3^, Cz), 122.9 (*ipso*-C^1^, Cz), 116.8 (CH^4^, Cz), 77.5 (C(CH_3_)_2_ CAAC), 65.2 (C–C_Carbene_ CAAC), 48.0 (CH_2_ CAAC), 39.0 (CH_2_ adamantyl), 37.3 (CH adamantyl), 35.4 (CH_2_ adamantyl), 34.7 (s, CH_2_ adamantyl), 29.6 (CH *i*Pr), 29.4 (s, C(CH_3_)_2_ CAAC), 28.0 (CH adamantyl), 27.6 (CH adamantyl), 26.7 (CH_3_*i*Pr), 23.6 (CH_3_*i*Pr), 21.3 (3,6-CH_3_, Cz), 20.6 (1,8-CH_3_, Cz). Anal. Calcd for C_43_H_55_AuN_2_ (796.40): C, 64.81; H, 6.96; N, 3.52. Found: C, 64.62; H, 6.90; N, 3.42. C_43_H_55_AuN_2_ theoretical [M+H^+^] = 797.4109, HRMS (APCI(ASAP)) = 797.4121.

#### Synthesis of (^Ad^CAAC)Au(5,12-Dihydro-5-phenyl-indolo[3,2-*a*]carbazole) (**4**)

Following the procedure described for **1**, the complex was made from (^Ad^CAAC)AuCl (1.82 g, 3 mmol), 5,12-dihydro-5-phenyl-indolo[3,2-*a*]-9*H*-carbazole (0.997 g, 3 mmol), and ^*t*^BuOK (0.338 g, 3 mmol) as a yellow solid. Yield: 2.65 g (2.9 mmol, 97%). Complex **4** is poorly soluble in a majority of common organic solvents (toluene, hexane, chlorobenzene, acetonitrile), and solubility increases upon heating.

^1^H NMR (300 MHz, CD_2_Cl_2_): δ 9.47 (d, *J* = 7.5 Hz, 1H), 7.99 (d, *J* = 8.5 Hz, 1H), 7.93–7.86 (m, 1H), 7.64–7.59 (m, 5H, N-Ph), 7.50–7.45 (m, 3H), 7.37 (d, *J* = 8.0 Hz, 1H), 7.30 (t, *J* = 7.5 Hz, 1H), 7.22 (t, *J* = 7.4 Hz, 1H), 7.03 (d, *J* = 8.4 Hz, 1H), 6.98–6.91 (m, 2H), 6.51–6.42 (m, 1H), 3.70 (d, *J* = 12.5 Hz, 2H, CH_2_ adamantyl), 3.05 (sept, *J* = 6.6 Hz, 2H, CH *i*Pr), 2.51 (s, 2H, CH_2_ CAAC), 2.16–1.59 (m, 12H, adamantyl), 1.50 (s, 6H, C(CH_3_)_2_ CAAC), 1.45 (d, *J* = 6.6 Hz, 6H, CH_3_*i*Pr), 1.37 (d, *J* = 6.6 Hz, 6H, CH_3_*i*Pr). ^13^C{^1^H} NMR (75 MHz, CD_2_Cl_2_): δ 244.3 (C_carbene_ CAAC), 155.7 (*ipso*-C–N, Cz), 145.9 (*o*-C Dipp), 141.0 (*ipso*-C, Cz), 140.2 (*ipso*-C, Cz), 138.9 (*ipso*-C, Cz), 136.5 (*ipso*-C Dipp), 130.1 (*p*-CH Dipp), 130.0 (CH, Cz), 128.1 (CH, Cz), 127.5 (CH, Cz), 125.7 (*m*-CH Dipp), 124.7 (CH, Cz), 123.6 (CH, Cz), 123.4 (CH, Cz), 121.5 (CH, Cz), 119.5 (*ipso*-C, Cz), 118.3 (CH, Cz), 118.2 (CH, Cz), 117.8 (CH, Cz), 117.0 (CH, Cz), 116.0 (CH, Cz), 109.5 (*ipso*-C, Cz), 108.9 (CH, Cz), 100.3 (CH, Cz), 77.5 (C(CH_3_)_2_ CAAC), 64.8 (C–C_Carbene_ CAAC), 48.3 (CH_2_ CAAC), 38.7 (CH_2_ adamantyl), 37.3 (CH adamantyl), 35.7 (CH_2_ adamantyl), 34.6 (CH_2_ adamantyl), 29.6 (CH *i*Pr), 29.5 (C(CH_3_)_2_ CAAC), 27.8 (CH adamantyl), 27.5 (CH adamantyl), 26.8 (CH_3_*i*Pr), 23.5 (CH_3_*i*Pr). Anal. Calcd for C_51_H_54_AuN_3_ (905.98): C, 67.61; H, 6.01; N, 4.64. Found: C, 67.79; H, 6.32; N, 4.45. C_51_H_54_AuN_3_ theoretical [M+H^+^] = 906.4061, HRMS (APCI(ASAP)) = 906.4083.

### Theory and Computational Details

All calculations were performed with the Q-Chem 5.0 quantum chemistry package.^73^ Optimizations were done in the gas phase using the DFT and TD-DFT methods for the ground and excited states, respectively, with the PBE0 functional^74^ and the def2-SVP basis set^75^ with the corresponding electron core potential on the Au atom.^76^ Electronic structure calculations were performed at the optimized geometry with the PBE0 functional and the larger def2-TZVP basis set with the corresponding ECP on the Au atom. An implicit dichloromethane solvent has been included with the COSMO approach with a dielectric constant of dichloromethane (ε = 9.08).^77^ The Tamm-Dancoff approximation (TDA)^78^ has been employed to avoid the overstabilization of low-lying intraligand triplet states. Conformation barriers were estimated from a relaxed torsion scan in the ground S_0_ state along the torsion angle: N1–C1–N2–C28.

## Supporting Information Available

Detailed experimental procedures, single-crystal X-ray diffraction data, photophysical and OLED device characterization, and computational details (PDF)
X-ray data (CIF)

## Supplementary Material

cm2c01938_si_001.pdf

cm2c01938_si_002.cif
