# Supplementary material for: Excited-State Lifetime Modulation by Twisted and Tilted Molecular Design in Carbene-Metal-Amide Photoemitters
Source: Chem Mater. 2022 Aug 4;34(16):7526–42. doi: 10.1021/acs.chemmater.2c01938 (PMC9404540; doi:10.1021/acs.chemmater.2c01938)
Supplement: Supplementary file 1 — cm2c01938_si_001.pdf [file cm2c01938_si_001.pdf]

Supporting Information for

Excited State Lifetime Modulation by Twisted and Tilted Molecular Design in Carbene-Metal-Amide Photoemitters

Qinying Gu, Florian Chotard, Julien Eng, Antti-Pekka M. Reponen, Inigo J. Vitorica-Yrezabal, Adam W. Woodward, Thomas J. Penfold,* Dan Credgington, Manfred Bochmann,* Alexander S. Romanov*

Table of Contents

NMR spectroscopy	p. S3
TGA curves for the complexes	p. S18
Electrochemistry	p. S19
X-ray Crystallography	p. S19
Photophysical characterisation	p. S26
OLED device fabrication	p. S38
Computational details	p. S47

**Table S1.** Values for the rate equations for complexes **1–4**.	p. S34
**Table S2.** Additional photophysical characterisation for complex **3** and **4**.	p. S38
**Table S3.** OLED performance summary of complex **1**	p. S41
**Table S4.** OLED performance summary of complex **2**	p. S42
**Table S5.** OLED performance summary of complex **3**	p. S44
**Table S6.** OLED performance summary of complex **4**	p. S45
**Table S7.** Energy of the HOMO and LUMO (in eV) and their overlap (in %).	p. S55
**Table S8.** Coordinates for ground and excited states (S1 and T1) geometries	p. S56

NMR spectroscopy.

2-chloro-6-fluoro-*N*-(2-fluorophenyl)aniline.

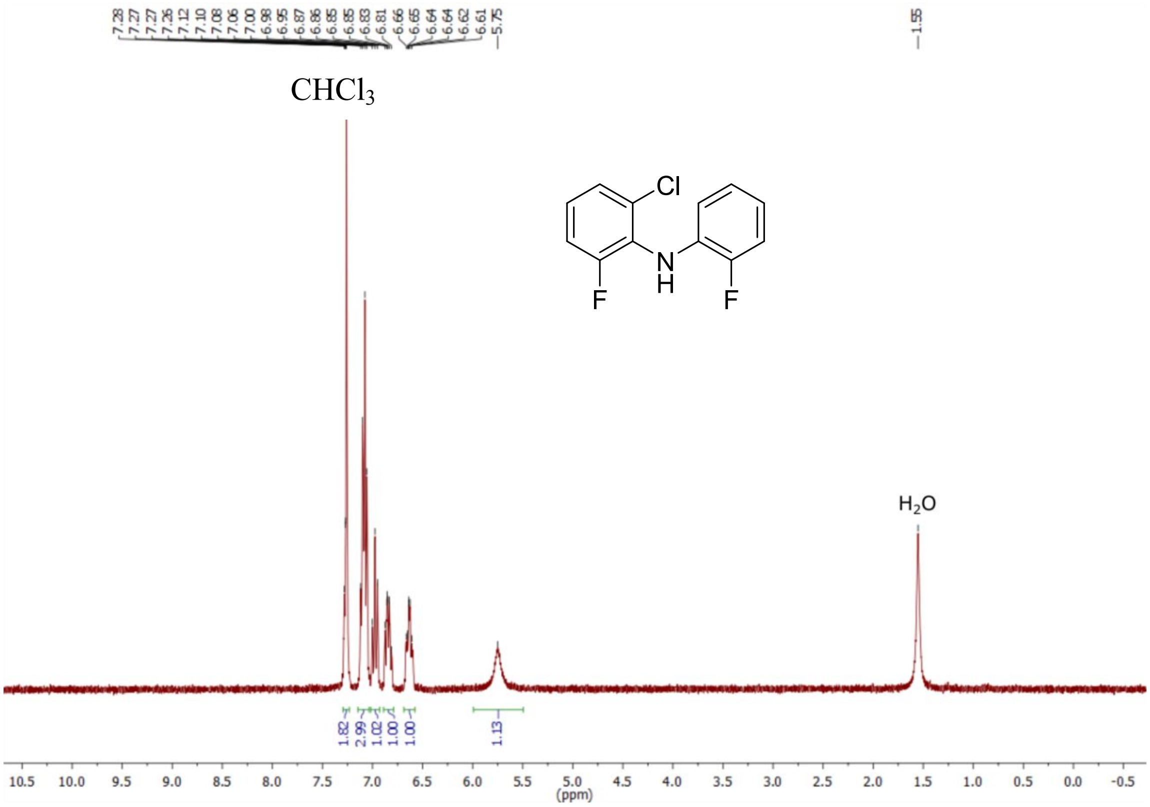

^{1}H NMR (300 MHz, $CDCl_3$)

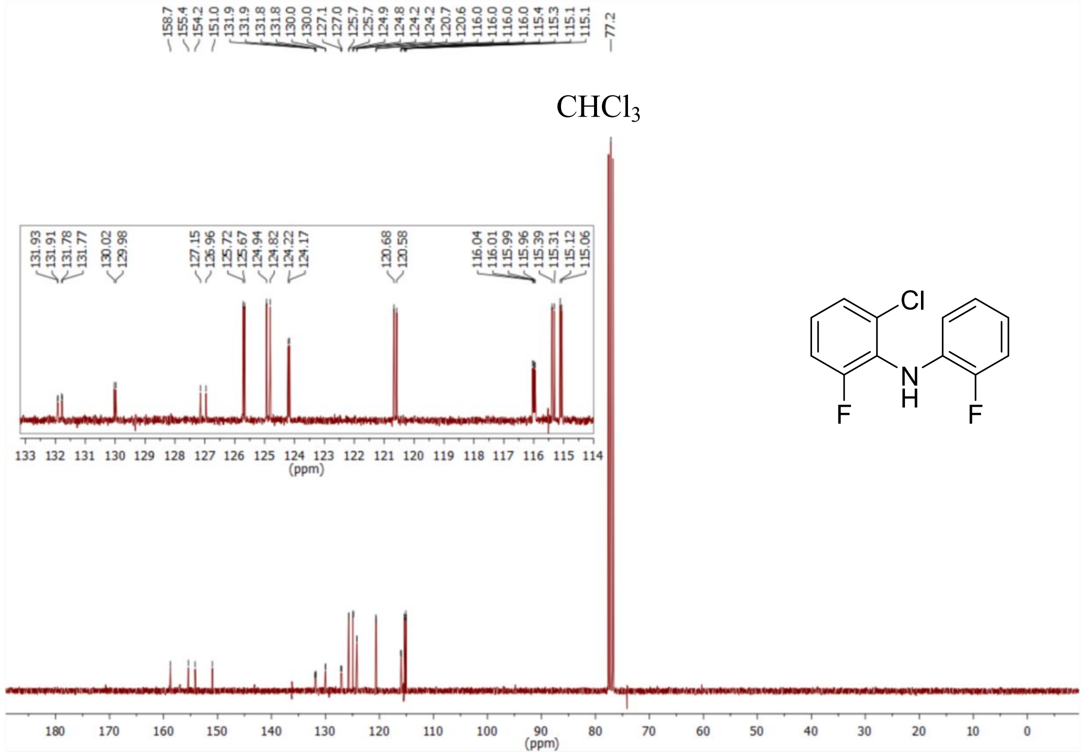

$^{13}C\{^1H\}$ NMR (75 MHz, $CDCl_3$)

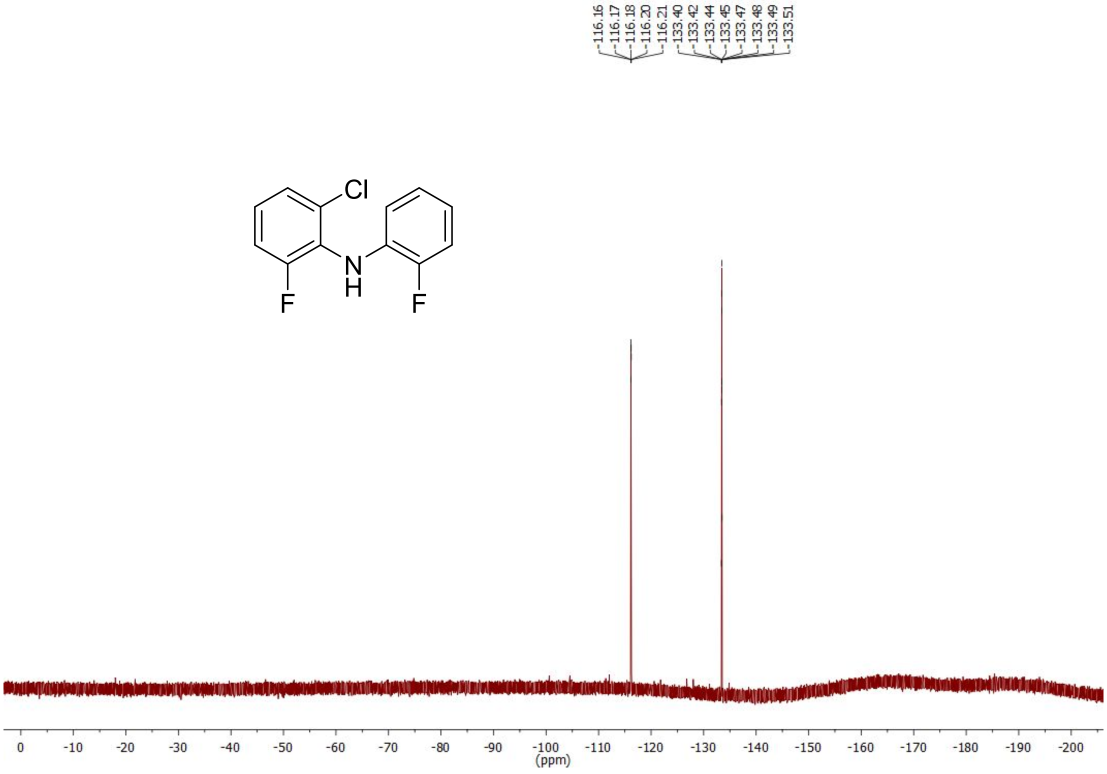

^{19}F NMR (282 MHz, $CDCl_3$)

1,8-difluoro-9H-carbazole (S2)

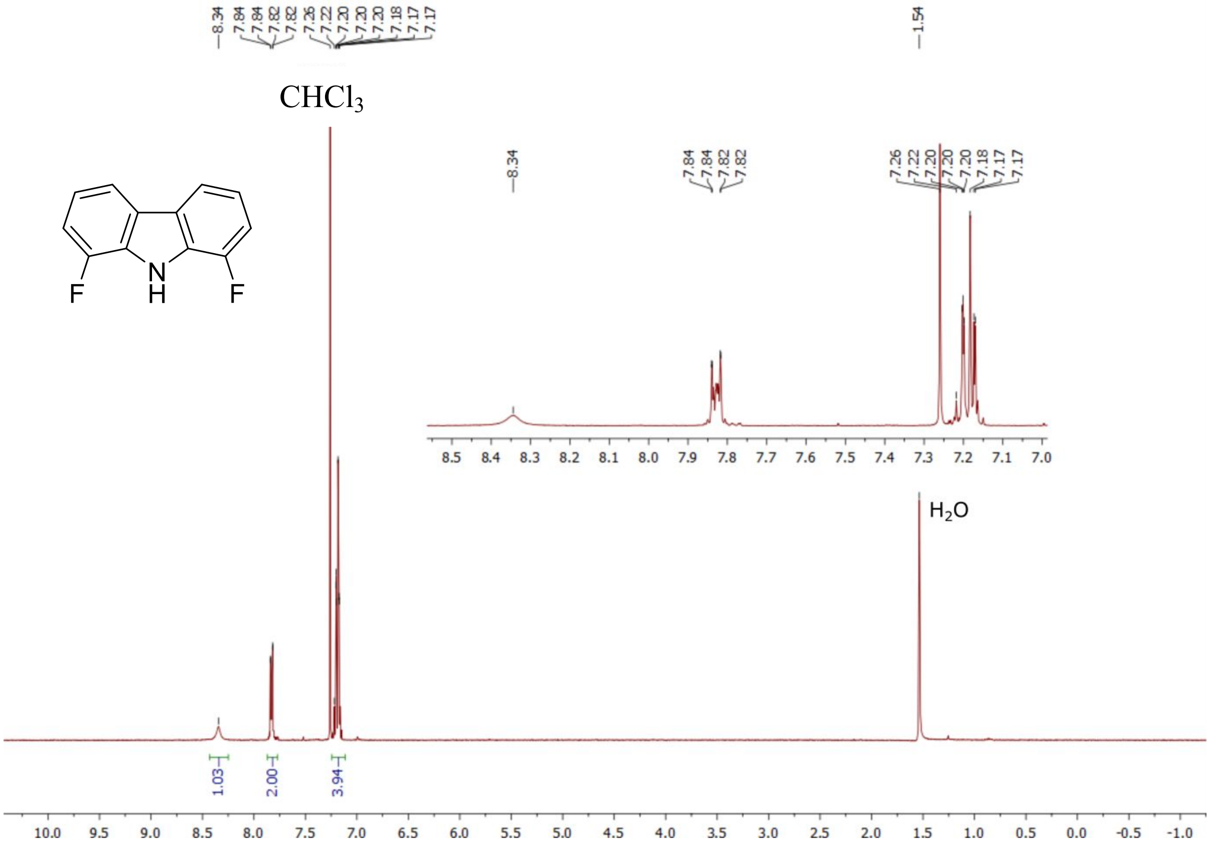

^{1}H NMR (300 MHz, $CDCl_3$)

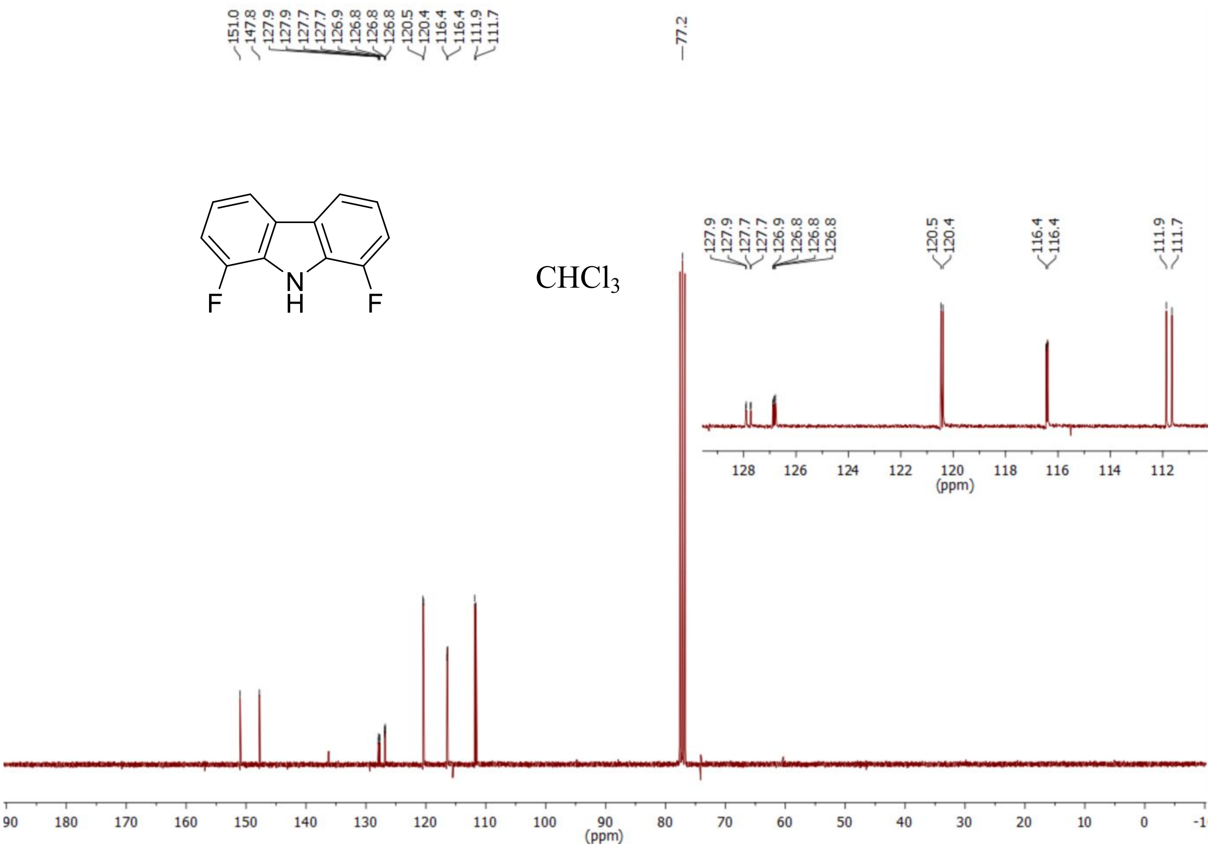

$^{13}C\{^1H\}$ NMR (75 MHz, $CDCl_3$)

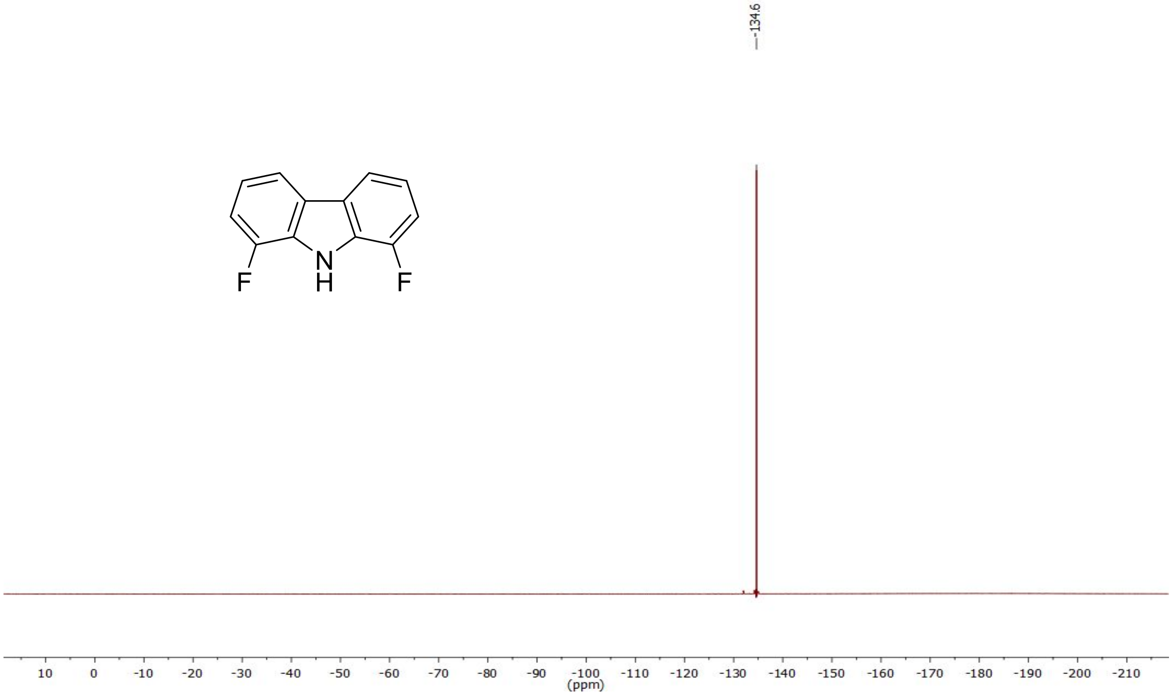

$^{19}F\{1H\}$ NMR (282 MHz, $CDCl_3$)

$(^{Ad}CAAC)Au(1,8\text{-difluorocarbazole})$ (1).

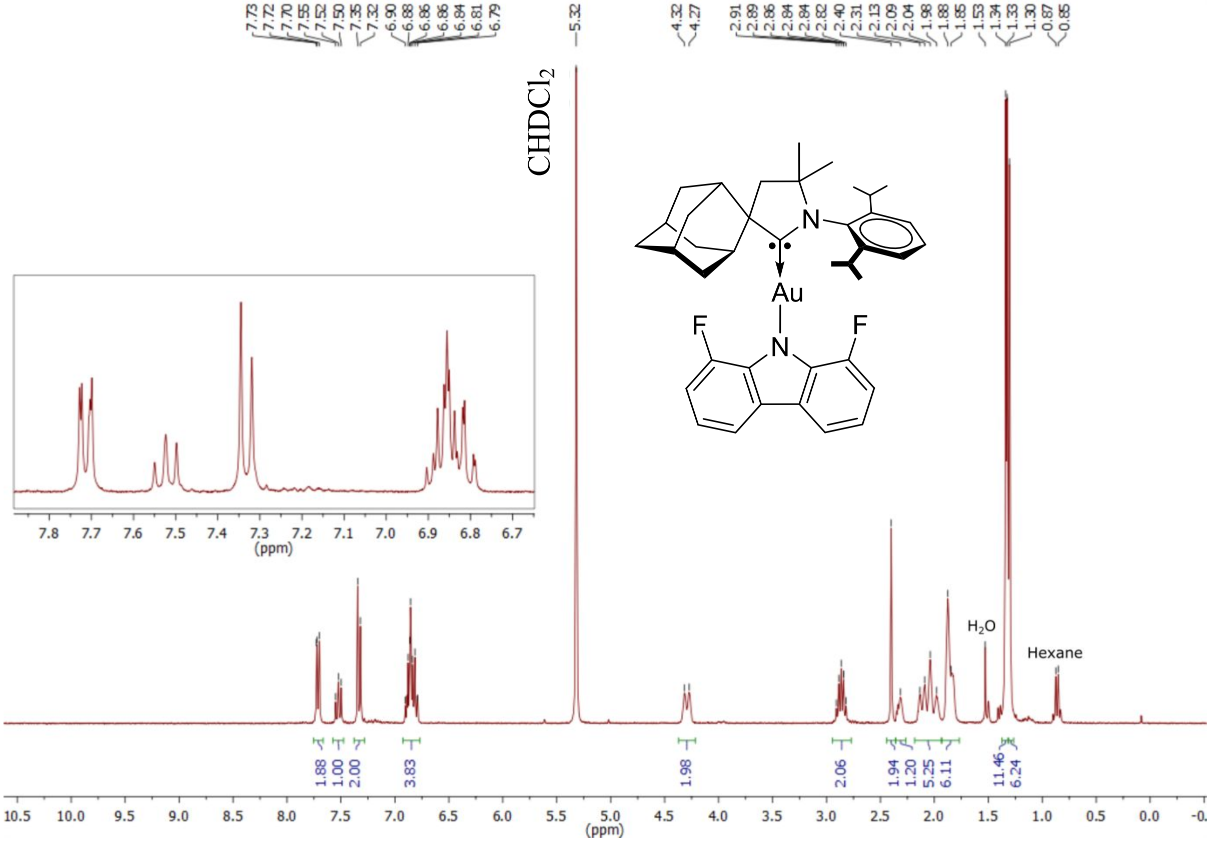

^{1}H NMR (300 MHz, CD_2Cl_2)

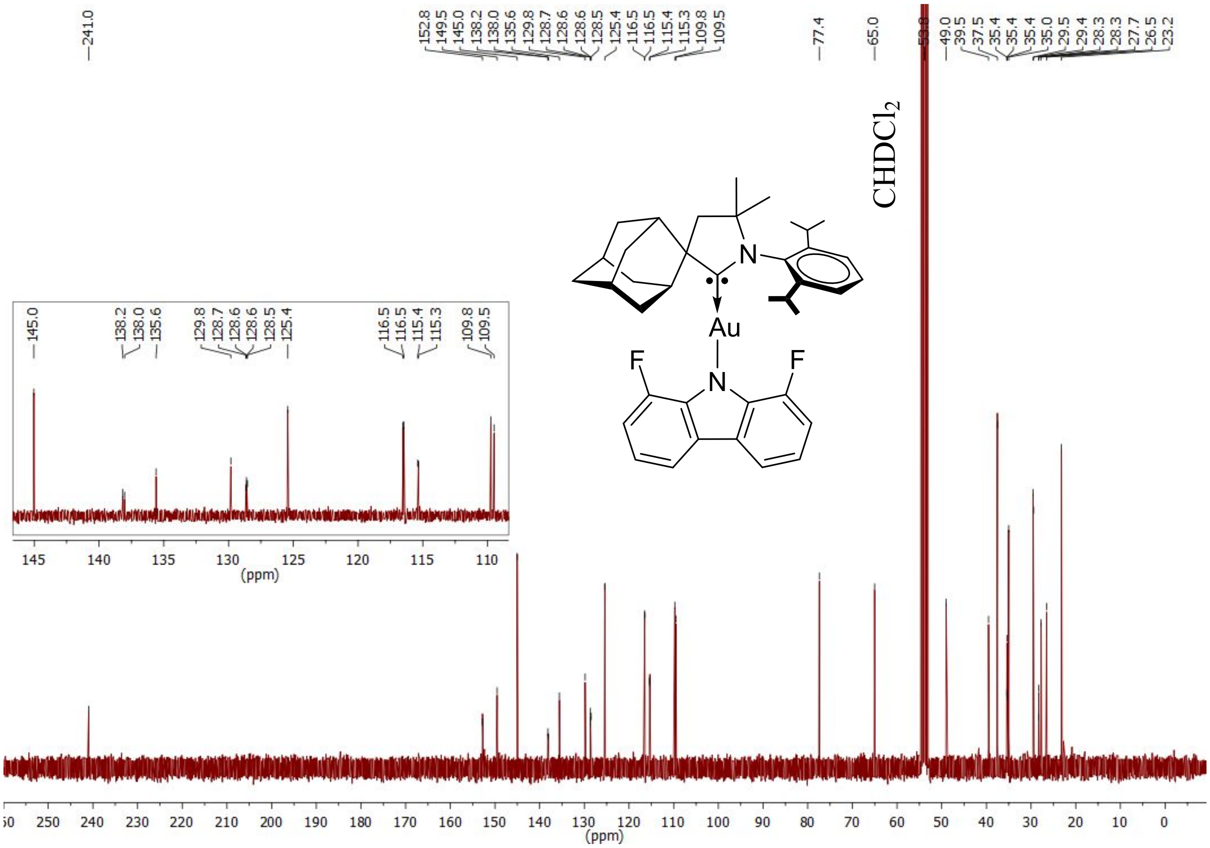

$^{13}C\{^{1}H\}$ NMR (75 MHz, CD_2Cl_2)

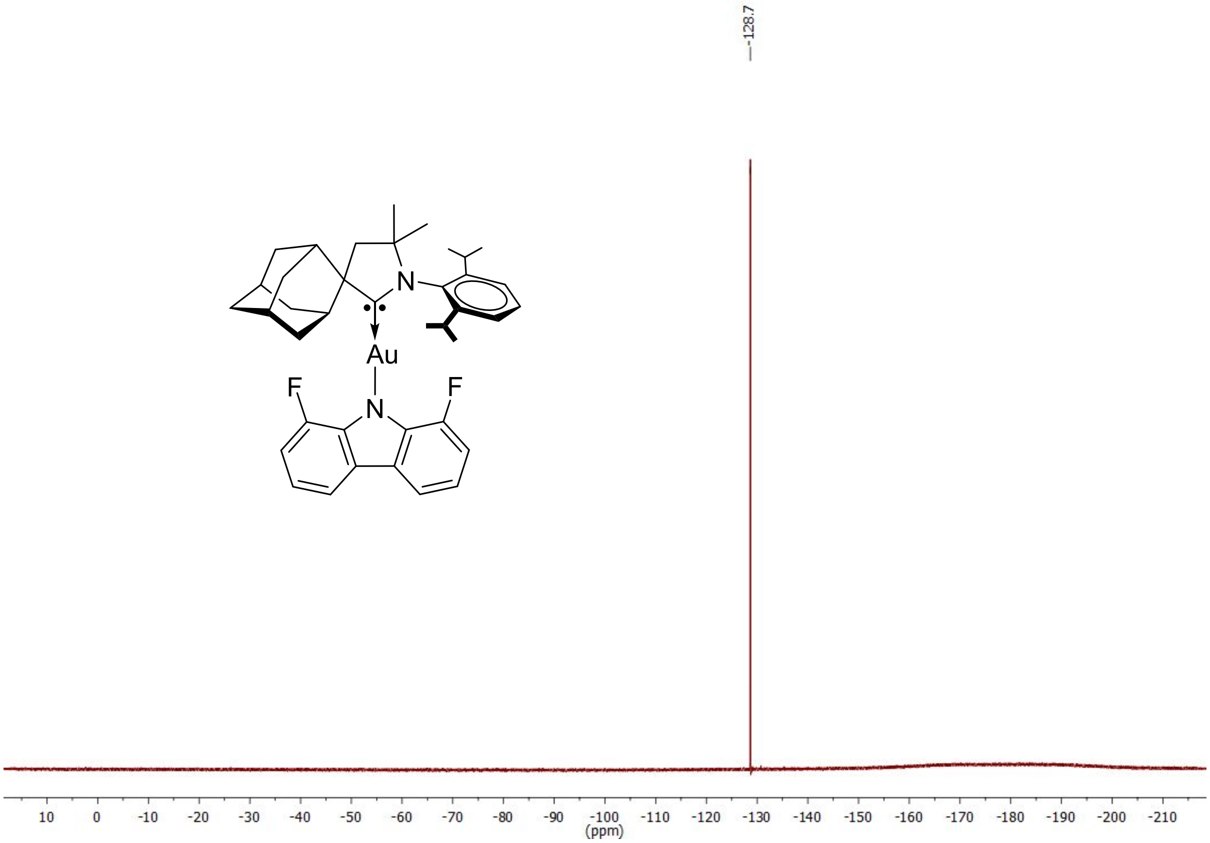

$^{19}F\{1H\}$ NMR (282 MHz, CD_2Cl_2)

(^{Ad}CAAC)Cu(1,8-difluorocarbazole) (2).

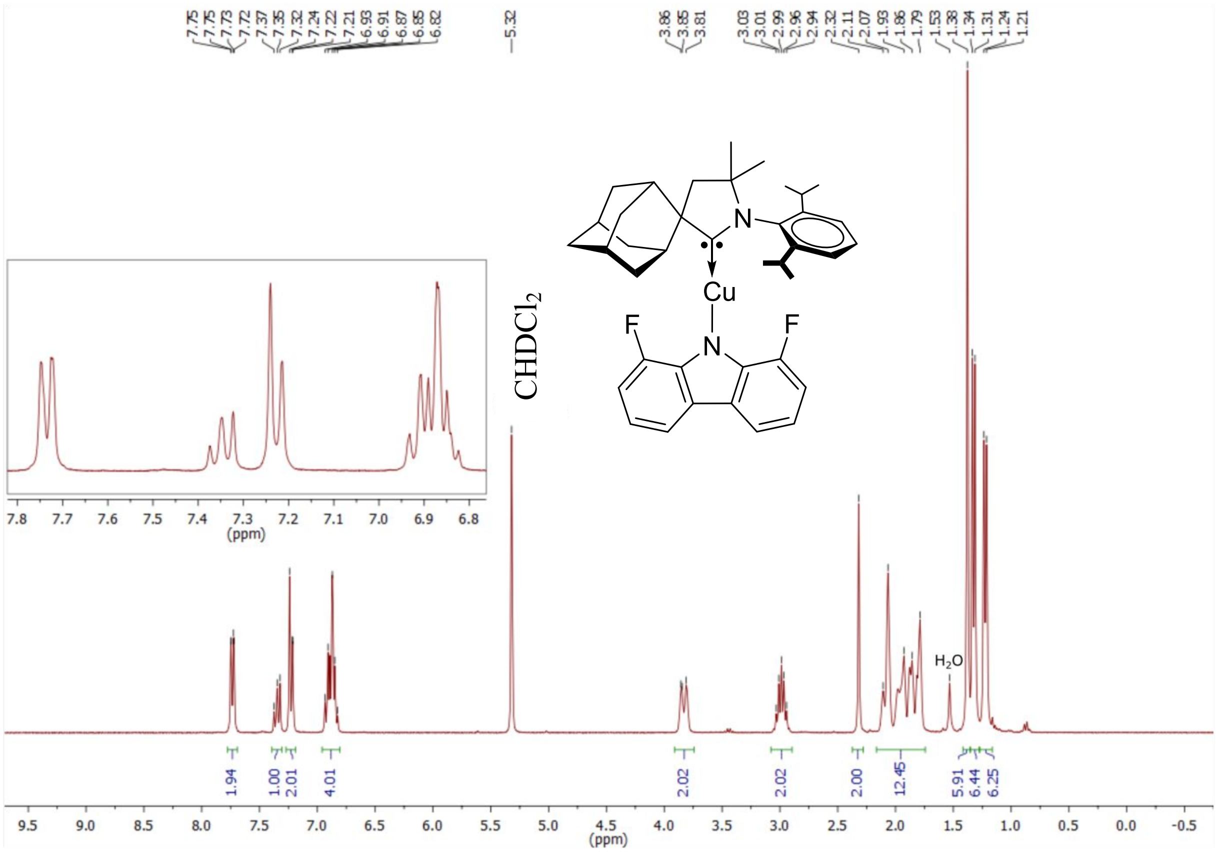

^1H NMR (300 MHz, CD_2Cl_2)

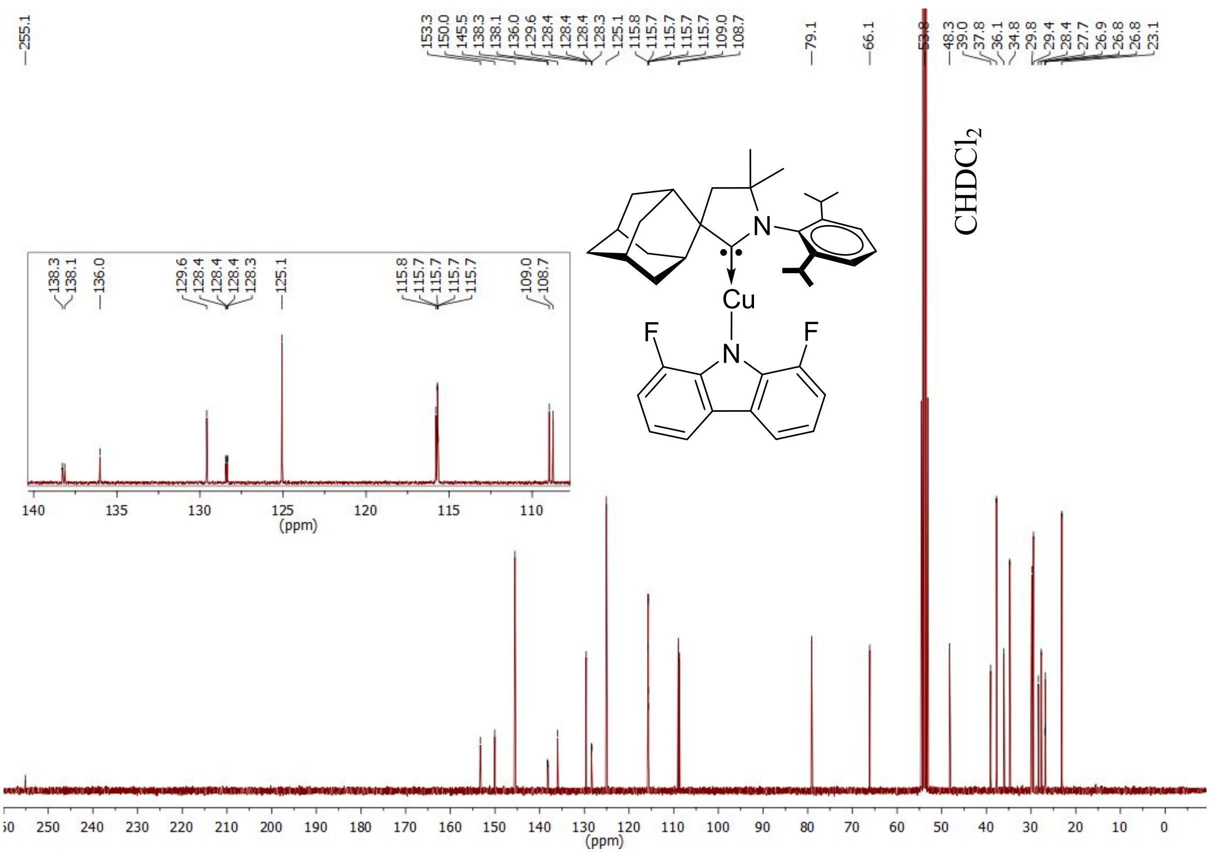

$^{13}C\{^1H\}$ NMR (75 MHz, CD_2Cl_2)

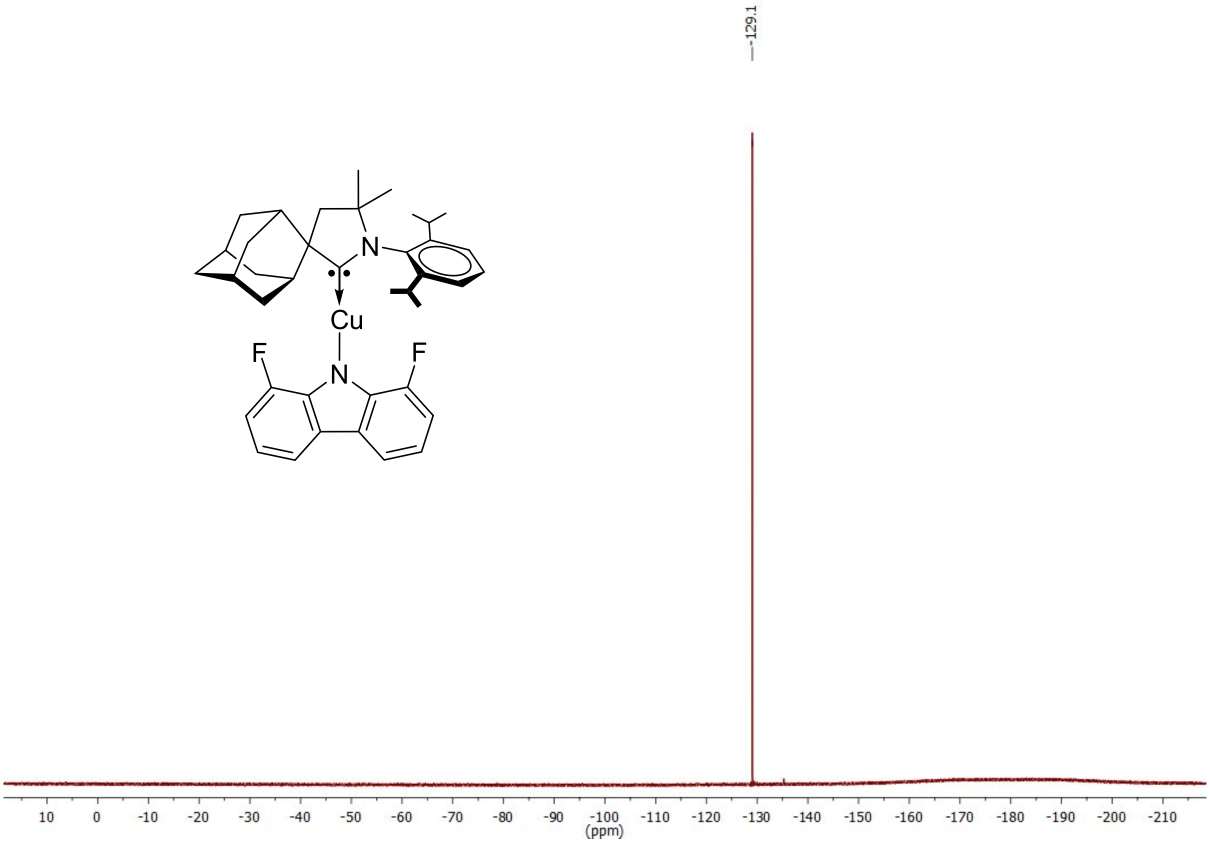

^{19}F NMR (282 MHz, CD_2Cl_2)

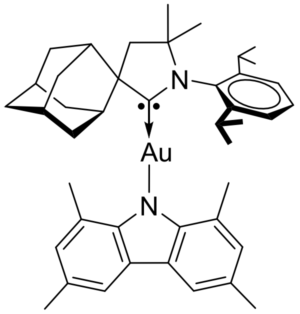

(^{Ad}CAAC)Au(1,3,6,8-tetrametylcarbazole).

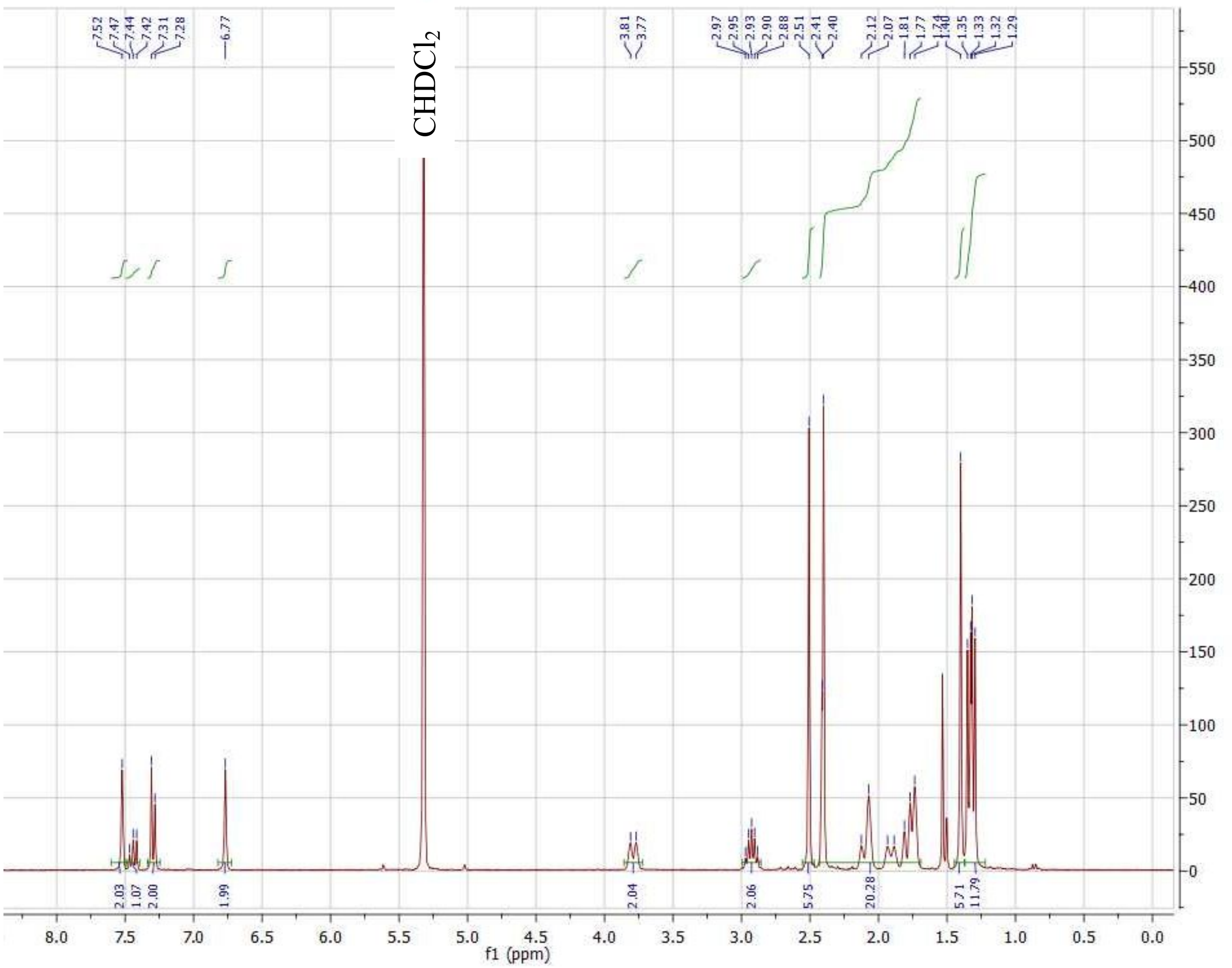

^{1}H NMR (300 MHz, CD_2Cl_2)

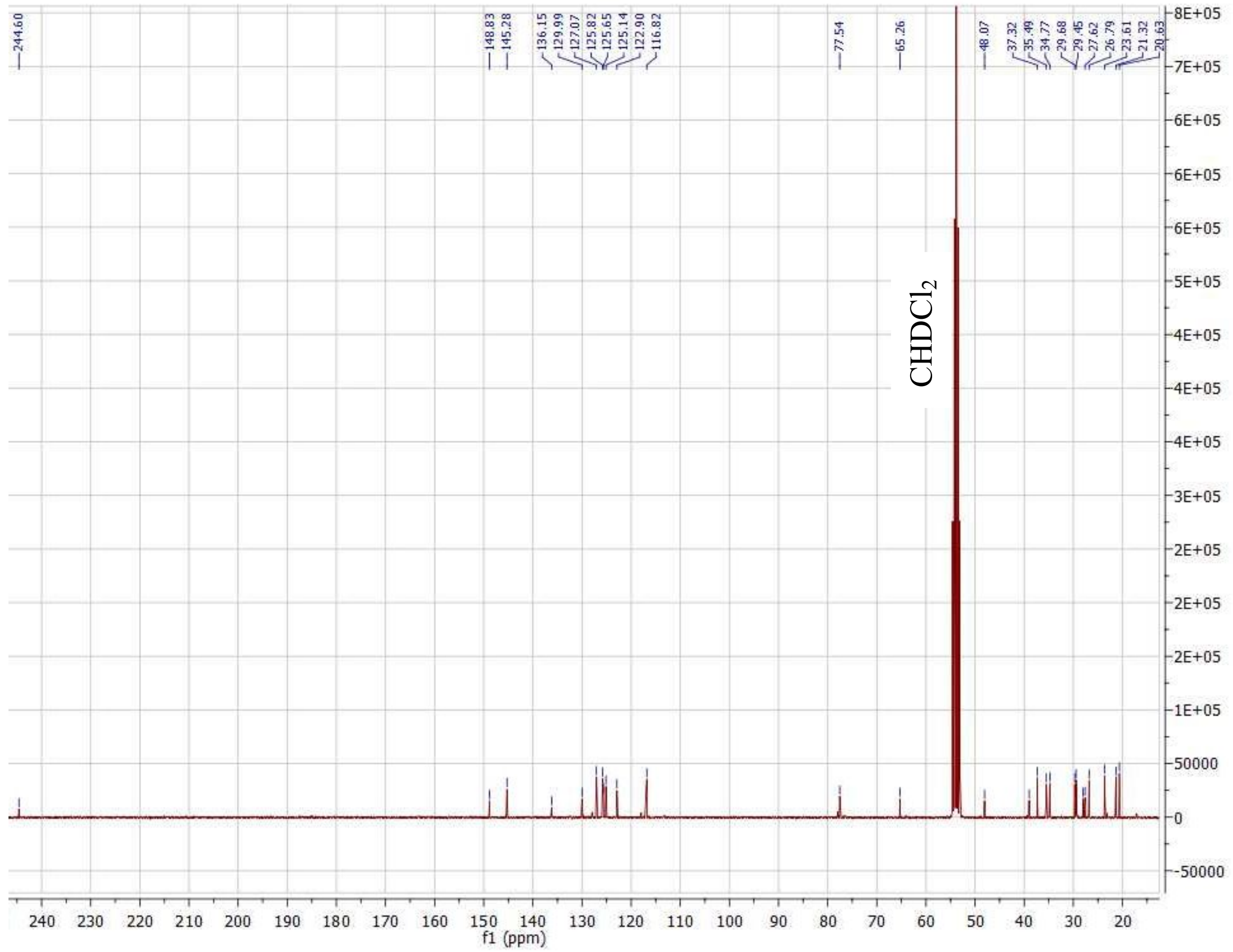

$^{13}C\{^{1}H\}$ NMR (75 MHz, CD_2Cl_2)

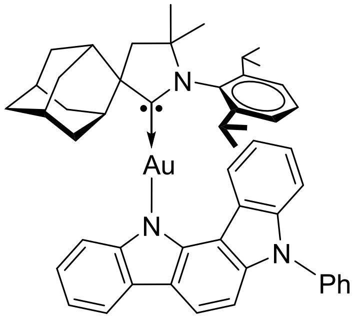

(^{Ad}CAAC)Au(5,12-dihydro-5-phenyl-indolo[3,2-*a*]carbazole) (4)

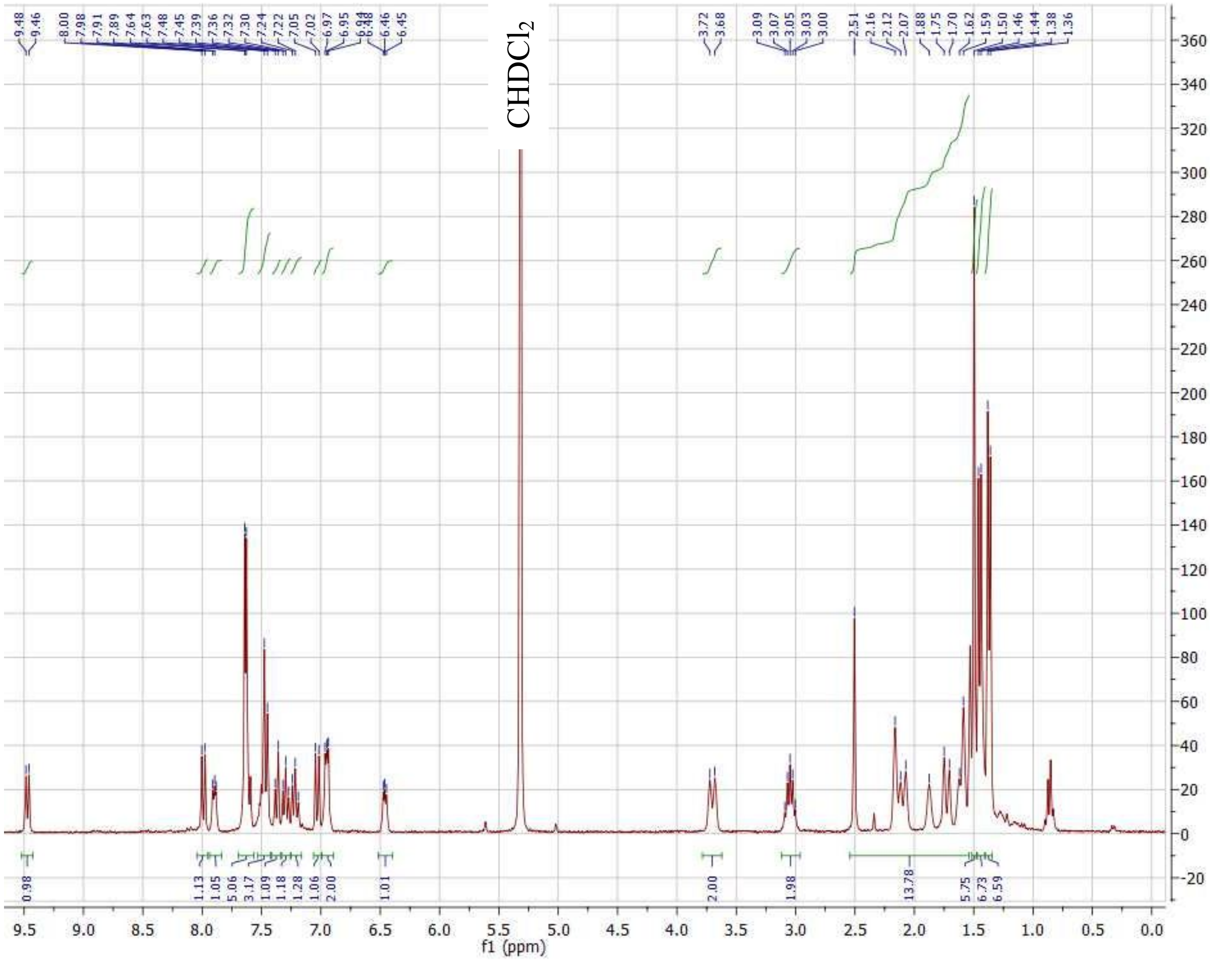

^{1}H NMR (300 MHz, CD_2Cl_2)

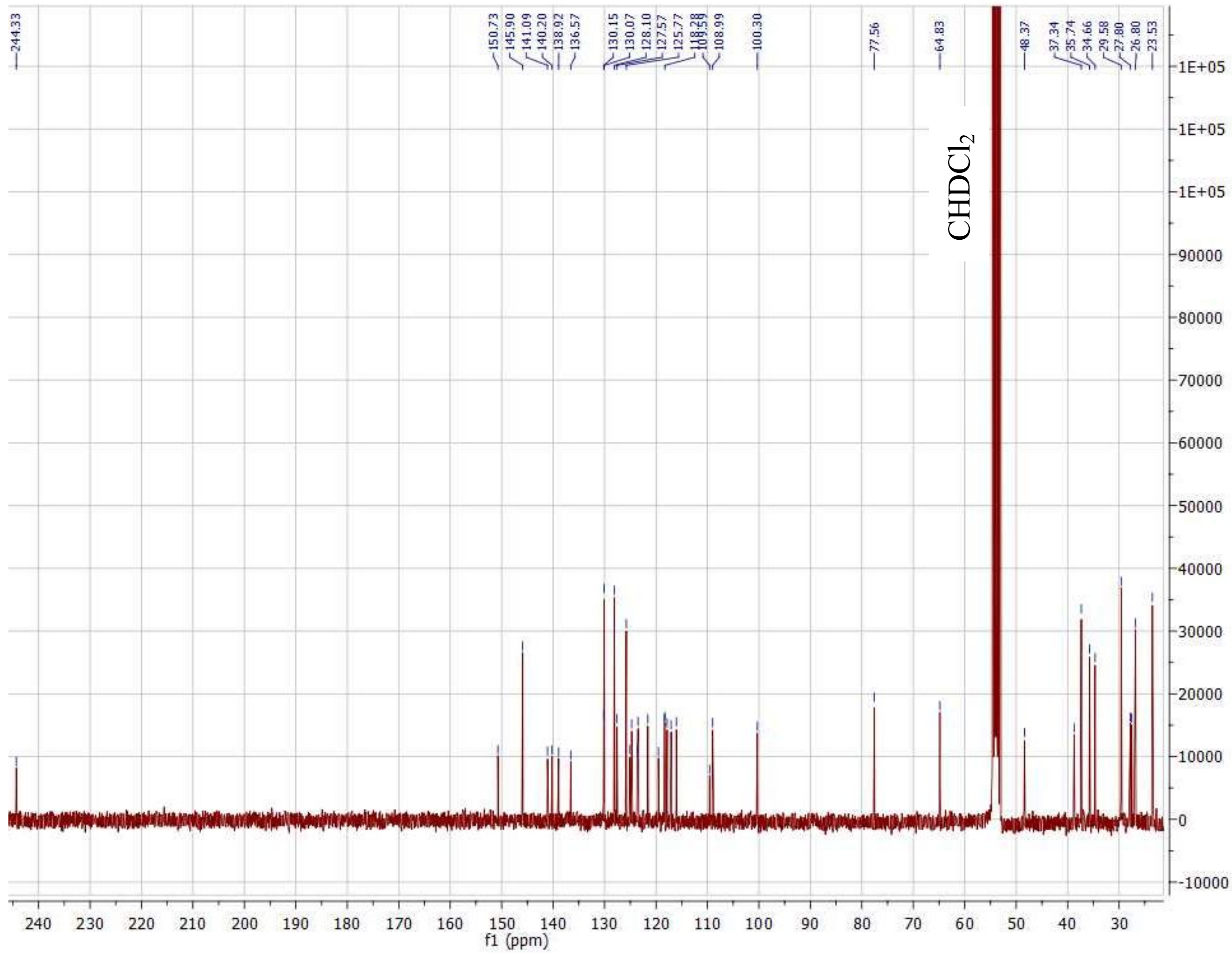

$^{13}C\{^{1}H\}$ NMR (75 MHz, CD_2Cl_2)

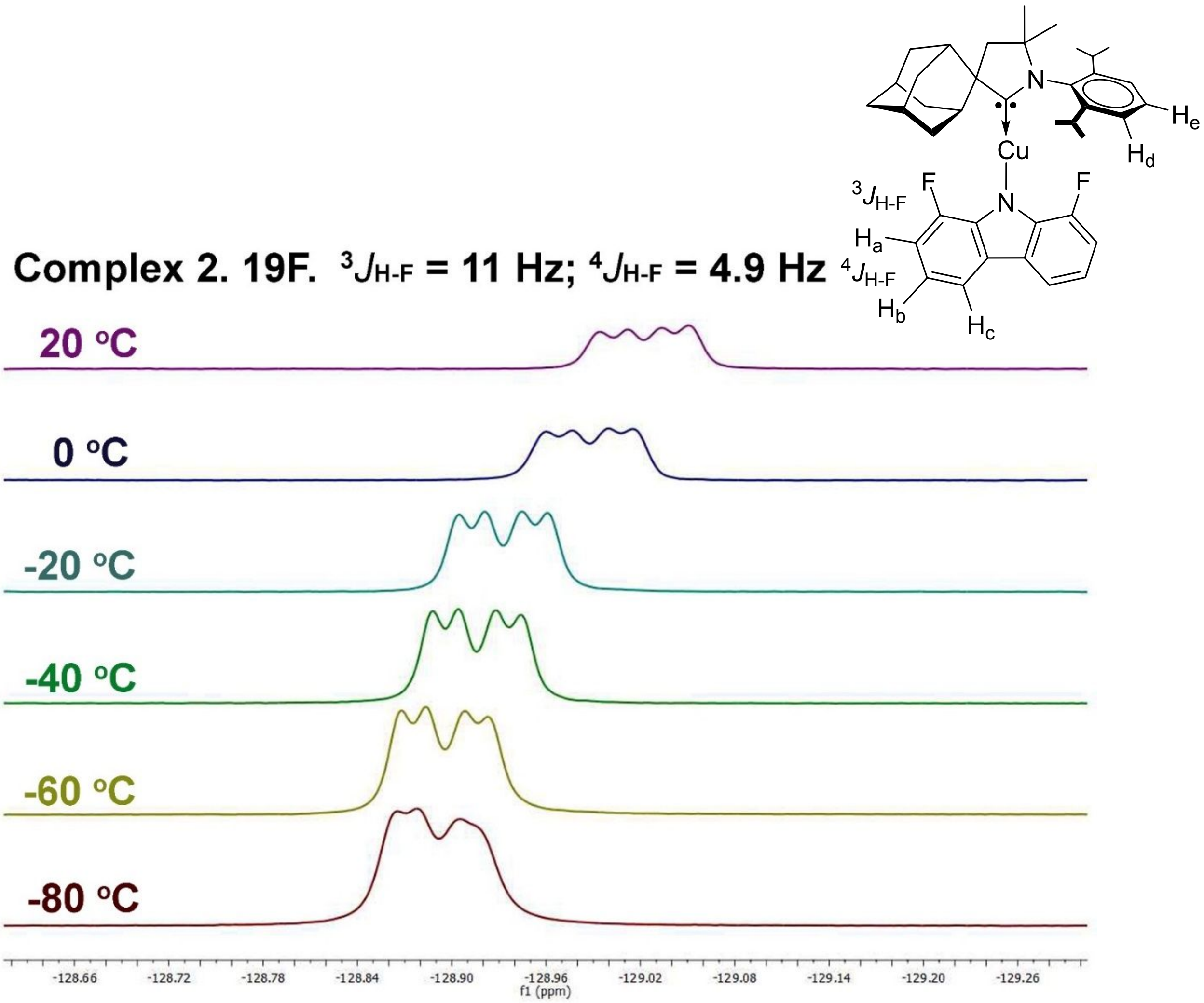

Figure S1. VT NMR ^{19}F (282 MHz) in DCM-d^2 for complex **2.**

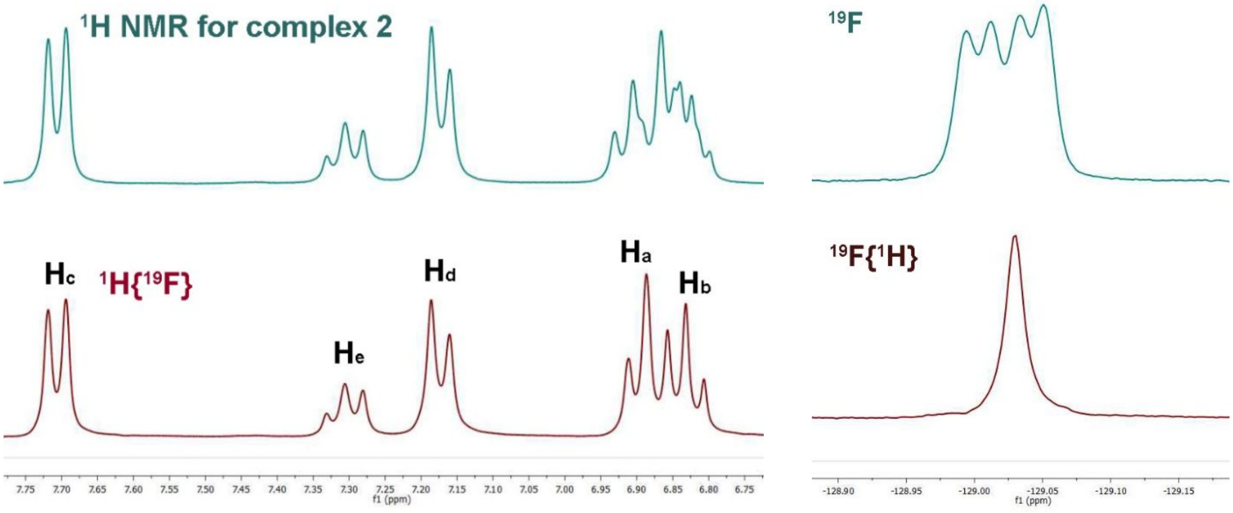

Figure S2. NMR ^{1}H{^{19}F} (bottom left) and ^{1}H (top left) in DCM-d^2 (282 MHz) for complex **2** at 20 °C and NMR ^{19}F{^{1}H} (bottom right) and ^{19}F (top right). The spin-splitting of protons H_a and H_b is caused by ^{19}F atoms.

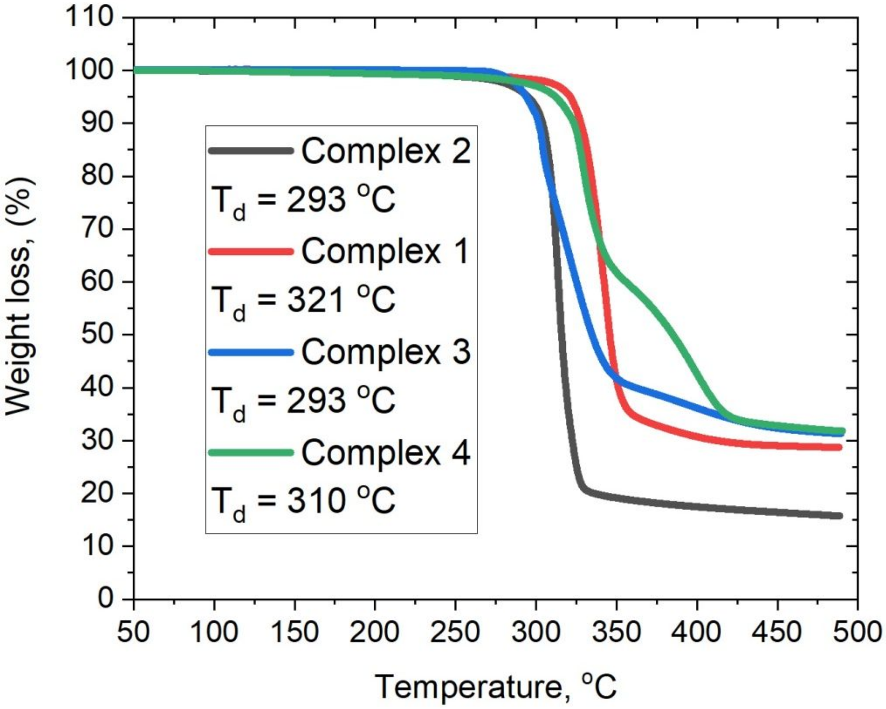

Figure S3. TGA curves for the complexes **1–4**.

Electrochemistry.

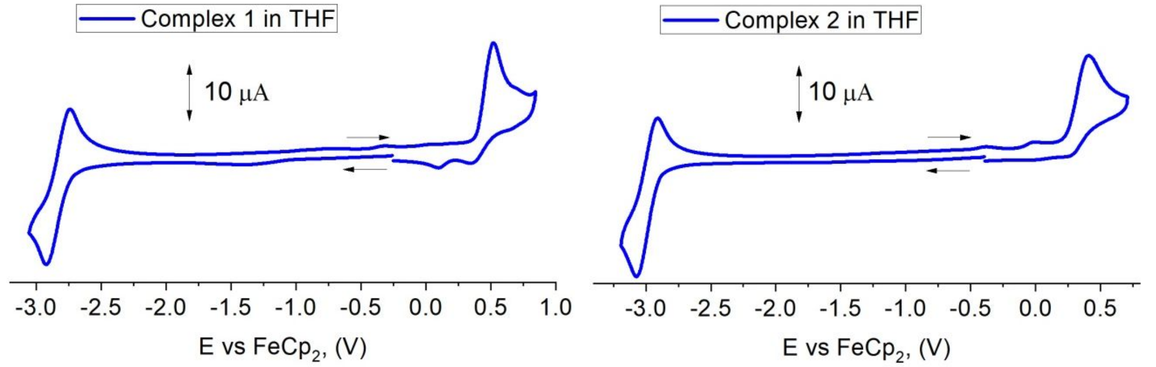

Figure S4. Full range cyclic voltammogram for gold **1** and copper complex **2**. Recorded using a glassy carbon electrode in THF solution (1.4 mM) with $[n\text{-}Bu_4N]PF_6$ as supporting electrolyte (0.13 M), scan rate 0.1 Vs^{-1}.

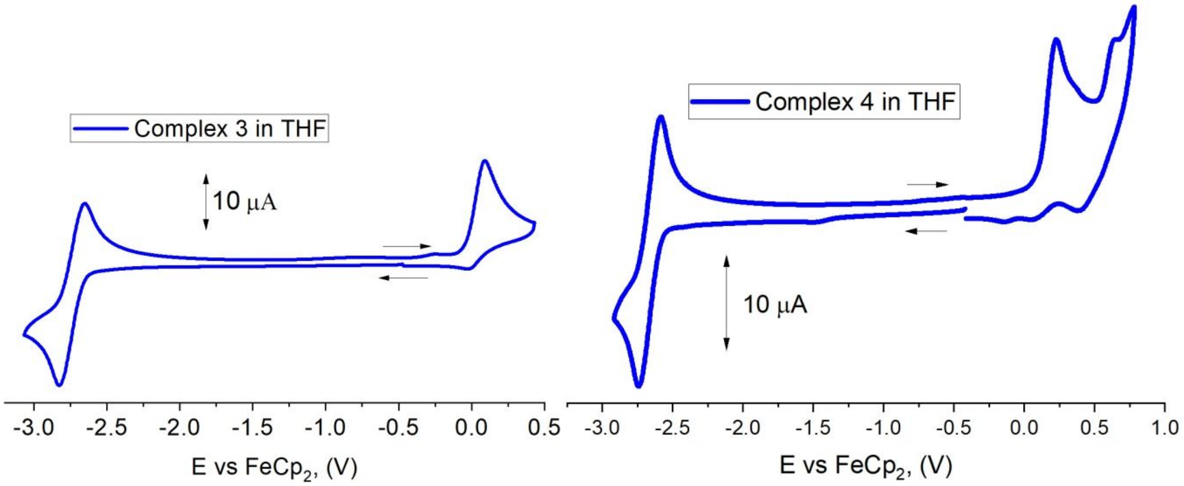

Figure S5. Full range cyclic voltammogram for the gold complex **3** and **4**. Recorded using a glassy carbon electrode in THF solution (1.4 mM) with $[n\text{-}Bu_4N]PF_6$ as supporting electrolyte (0.13 M), scan rate 0.1 Vs^{-1}.

X-Ray Crystallography.

Complex **1** crystallizes with two independent molecules in the unit cell. The atoms C3, C26 and C27 were disordered over two half-populated positions for complex **1**. The unit cell for monoclinic polymorph of complex **2** contains two independent molecules whereas triclinic polymorph of **2** contains three independent molecules and one 2-methyl-pentane molecules as a solvate. Further analysis of the residual electron density for the triclinic polymorth of **2** indicates the presence of two additional and severely disordered 2-methylpentane molecules which

contribution was removed from the diffraction data with PLATON/SQUEEZE for the final refinement.[1,2] The unit cell for of complex **3·Benzene** contains two independent molecules of **3** and two co-crystallized benzene molecules. The unit cell for of complex **3·CH₂Cl₂** contains one independent molecule of **3** and one co-crystallized CH_2Cl_2 molecule. Crystals were mounted in oil on glass fiber and fixed on the diffractometer in a cold nitrogen stream. Data were collected using an Oxford Diffraction Xcalibur-3/Sapphire3-CCD diffractometer with graphite monochromated Mo K_α radiation (λ = 0.71073 Å) at 140 K. Data were processed using the CrystAlisPro-CCD and –RED software.[3] The structure was solved by direct methods and refined by the full-matrix least-squares against F^2 in an anisotropic (for non-hydrogen atoms) approximation. All hydrogen atom positions were refined in isotropic approximation in a "riding" model with the $U_{iso}(H)$ parameters equal to 1.2 $U_{eq}(C_i)$, for methyl groups equal to 1.5 $U_{eq}(C_{ii})$, where $U(C_i)$ and $U(C_{ii})$ are respectively the equivalent thermal parameters of the carbon atoms to which the corresponding H atoms are bonded. All calculations were performed using the SHELXTL software.[4]

The principal crystallographic data and refinement parameters:

Crystals suitable for X-ray diffraction study were obtained by layering the CH_2Cl_2 solution of complex **1** hexanes at room tempearature followed by cooling at -20 °C. CCDC number 1956198, $C_{39}H_{45}AuF_2N_2$, Orthorhombic, space group $P2_12_12_1$, a = 9.4894(3) Å, b = 15.5514(6) Å, c = 21.9698(7) Å, V = 3242.16(19) Å^3, Z = 4, d_{calc} = 1.591 g cm^{-3}, μ = 4.580 mm^{-1}, colourless/block, crystal size 0.36 × 0.24 × 0.20 mm, F(000) = 1560, T_{min}/T_{max} 0.36383/1.00000, R_1 = 0.0289 (from 7835 unique reflections with I>2σ(I); R_{int} = 0.0482, R_{sigma} = 0.0422) and wR_2 = 0.0603 (from all 37125 reflections), GOF = 1.019, $\Delta\rho_{min}/\Delta\rho_{max}$ = 0.99/–0.88. Another crystallization attempt by layering the toluene solution resulted in identical unit cell parameters and no inclusion of the solvated molecules: $C_{39}H_{45}AuF_2N_2$, Orthorhombic, space group $P2_12_12_1$, a = 9.4987(4) Å, b = 15.459(1) Å, c = 21.9894(8) Å, V = 3229.1(3) Å^3, Z = 4, d_{calc} = 1.598 g cm^{-3}, μ = 4.598 mm^{-1}, colourless/block, crystal size 0.21 × 0.17 × 0.16 mm, F(000) = 1560, T_{min}/T_{max} 0.32107/1.00000, R_1 = 0.0802 (from 6351 unique reflections with I>2σ(I); R_{int} = 0.1391, R_{sigma} = 0.0422) and wR_2 = 0.1901 (from all 30781 reflections), GOF = 1.138, $\Delta\rho_{min}/\Delta\rho_{max}$ = 2.66/–3.88.

Crystals of complex **2** suitable for X-ray diffraction study were obtained by cooling a saturated CH_2Cl_2:2-methylpentane (1:9) solution at -20 °C to give a triclinic polymorph. CCDC

number 2027574, $C_{41}H_{49.7}CuF_2N_2$, Triclinic, space group *P-1*, a = 16.6891(7) Å, b = 17.6024(7) Å, c = 22.2861(6) Å, α = 107.079(3)°, β = 105.291(3)°, γ = 90.672(3)°, V = 6008.4(4) Å^3, Z = 6, d_{calc} = 1.114 g cm^{-3}, μ = 0.582 mm^{-1}, colorless/plate, crystal size 0.19 × 0.15 × 0.03 mm, F(000) = 2140.0, T_{min}/T_{max} 0.74046/1.00000, R_1 = 0.0750 (from 23619 unique reflections with I>2σ(I); R_{int} = 0.1000, R_{sigma} = 0.1297) and wR_2 = 0.1894 (from all 76057 reflections), *GOF* = 1.024, $\Delta\rho_{min}/\Delta\rho_{max}$ = 1.06/– 0.47.

Crystals of complex **2** suitable for X-ray diffraction study were obtained by layering saturated CH_2Cl_2 solution with hexanes at room temperature to give a monoclinic polymorph. CCDC number 1956197, $C_{39}H_{45}CuF_2N_2$, Monoclinic, space group *P2₁/c*, a = 18.3730(4) Å, b = 18.3497(4) Å, c = 19.5826(4) Å, β = 92.460(2)°, V = 6596.0(2) Å^3, Z = 8, d_{calc} = 1.296 g cm^{-3}, μ = 0.703 mm^{-1}, colorless/prism, crystal size 0.23 × 0.19 × 0.10 mm, F(000) = 2720.0, T_{min}/T_{max} 0.86893/1.00000, R_1 = 0.0402 (from 15904 unique reflections with I>2σ(I); R_{int} = 0.0439, R_{sigma} = 0.0443) and wR_2 = 0.1147 (from all 63044 reflections), *GOF* = 1.052, $\Delta\rho_{min}/\Delta\rho_{max}$ = 0.47/– 0.38.

Crystals of complex **3·Benzene** suitable for X-ray diffraction study were obtained by slow layering saturated benzene solution with hexanes at room temperature, CCDC number 2027575, $C_{49}H_{61}AuN_2$, Orthorhombic, space group $P2_12_12_1$, a = 18.6537(2) Å, b = 20.2335(2) Å, c = 21.4452(2) Å, V = 8094.05(14) Å^3, Z = 8, d_{calc} = 1.436 g cm^{-3}, μ = 3.671 mm^{-1}, yellow/prism, crystal size 0.37 × 0.29 × 0.23 mm, F(000) = 3584,0, T_{min}/T_{max} 0.69359 /1.00000, R_1 = 0.0248 (from 20607 unique reflections with I>2σ(I); R_{int} = 0.0442, R_{sigma} = 0.0401) and wR_2 = 0.0464 (from all 101737 unique reflections), *GOF* = 1.023, $\Delta\rho_{min}/\Delta\rho_{max}$ = 0.86/–0.51, Flack parameter 0.018(4).

Crystals of complex **3·CH₂Cl₂** suitable for X-ray diffraction study were obtained by layering saturated CH_2Cl_2 solution with hexanes at room tempearature followed by cooling at -20 °C, CCDC number 1956199, $C_{44}H_{57}AuCl_2N_2$, Monoclinic, space group $P2_1/n$, a = 13.3315(5) Å, b = 19.3773(5) Å, c = 15.9007(5) Å, β = 105.989(4)°, V = 3948.7(2) Å^3, Z = 4, d_{calc} = 1.483 g cm^{-3}, μ = 3.894 mm^{-1}, yellow/plate, crystal size 0.47 × 0.23 × 0.05 mm, F(000) = 1792, T_{min}/T_{max} 0.37970/1.00000, R_1 = 0.0230 (from 7740 unique reflections with I>2σ(I); R_{int} = 0.0322, R_{sigma} = 0.0234) and wR_2 = 0.0555 (from all 37479 unique reflections), *GOF* = 1.017, $\Delta\rho_{min}/\Delta\rho_{max}$ = 1.49/–1.19.

Crystals of desolvated complex **3** suitable for X-ray diffraction study were obtained by layering saturated CH_2Cl_2 solution with hexanes followed by removal of the crystalls from the mother solution and slow drying at room tempearature, CCDC number 2180575, $C_{43}H_{55}AuN_2$, Monoclinic, space group *P2₁/c*, a = 20.441(2) Å, b = 19.513(4) Å, c = 18.204(2) Å, β = 93.390(11)°, V = 7248(2) Å^3, Z = 8, d_{calc} = 1.460 g cm^{-3}, μ = 7.852 mm^{-1}, yellow/prism, crystal size 0.03 × 0.06 × 0.10 mm, F(000) = 3248, T_{min}/T_{max} 0.5870/1.00000, R_1 = 0.0595 (from 7317 unique reflections with I>2σ(I); R_{int} = 0.210) and wR_2 = 0.1466 (from all 12335 unique reflections), GOF = 0.997, $\Delta\rho_{min}/\Delta\rho_{max}$ = 0.80/–2.27.

The crystals of complex **4** were obtained by layering chlorobenzene/dichloromethane (1:1) solution with hexane at room temperature. CCDC number 1956200, $C_{51}H_{54}AuN_3$, Triclinic, space group *P-1*, a = 9.6336(8) Å, b = 9.9583(6) Å, c = 20.9277(10) Å, α = 91.113(4)°, β = 100.183(5)°, γ = 93.507(7)°, V = 1971.4(2) Å^3, Z = 2, d_{calc} = 1.526 g cm^{-3}, μ = 3.772 mm^{-1}, yellow/needle, crystal size 0.10 × 0.05 × 0.04 mm, F(000) = 920, T_{min}/T_{max} 0.30830/1.00000, R_1 = 0.0341 (from 7736 unique reflections with I>2σ(I); R_{int} = 0.0451, R_{sigma} = 0.0594) and wR_2 = 0.0795 (from all 18480 unique reflections), GOF = 1.025, $\Delta\rho_{min}/\Delta\rho_{max}$ = 1.44/–1.28.

Powder X-ray diffraction. The data was collected on the Rigaku Smartlab SE powder diffractometer in reflection mode with a Bragg-Brentano geometry using Cu Kα radiation (λ = 1.54184 Å) at 300 K. The crystals of complexes **1-4** were deposited intact on the Si zero-background sample holder. The sample spinning was applied to reduce the preferred orientation effects. The data was collected in the range of 6–60° with a 0.007 step size and speed 0.8° per min. The data was processed and analyzed with SMARTLAB STUDIO II software using different preferred orientation models – March[5] or spherical harmonics model[6] to account for the preferred crystallographic orientation. The whole-powder-pattern fitting (WPPF) technique was applied to reproduce the diffraction pattern, peak intensities and unit cell parameters for diffractograms presented on Fig. 2. The WPPF calculation including texture refinement allowed to estimate the concentration of the main phase as 95±10% where significant standard deviation originates due to severe preferred orientation of the polycrystalline material.

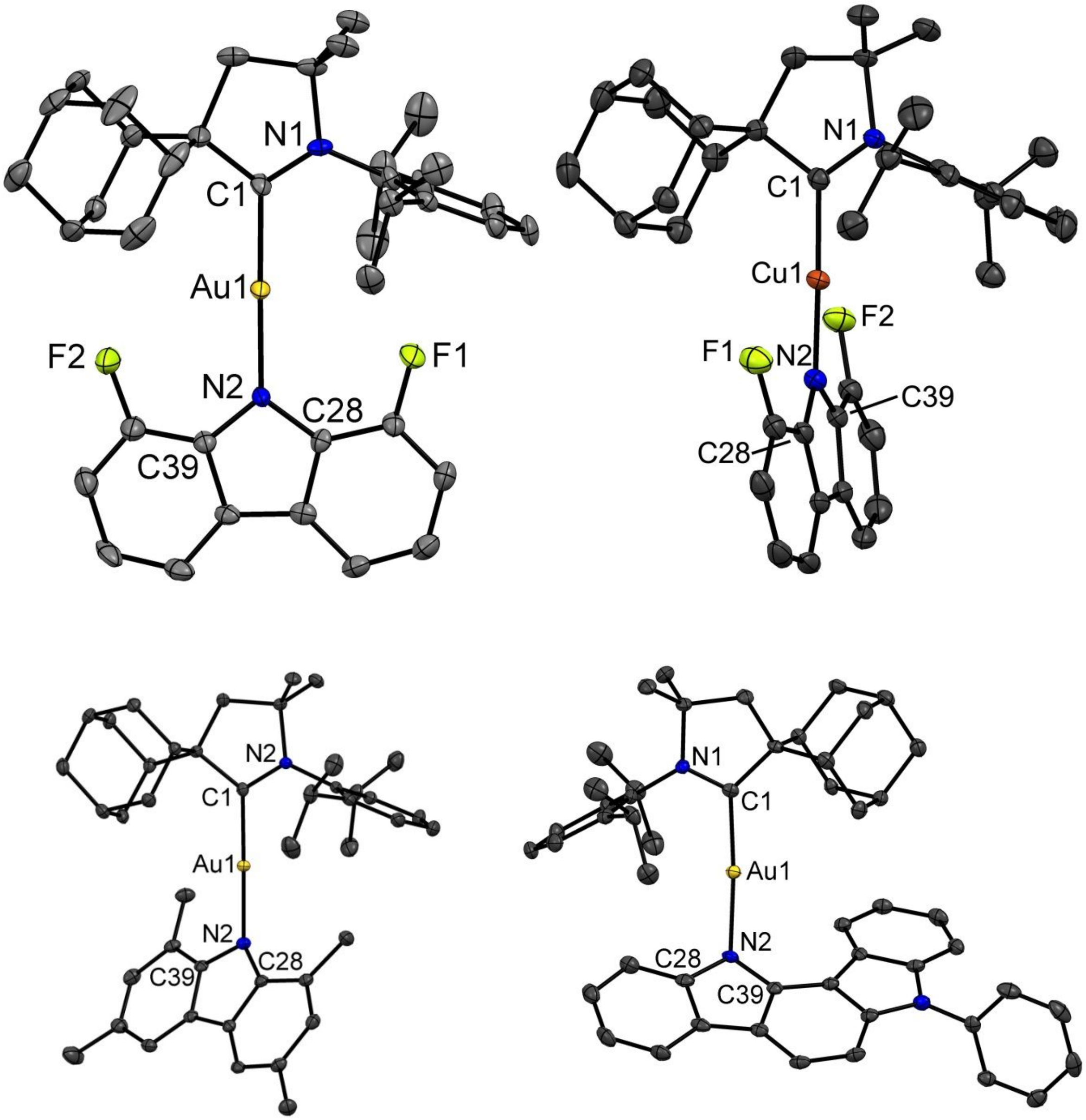

Figure S6. Crystal structures of complexes **1** (top left), **2** (top right), **3** (bottom left) and **4** (bottom right). Ellipsoids are shown at the 50% level. Hydrogen atoms and co-crystallized solvent molecules are omitted for clarity.

a)

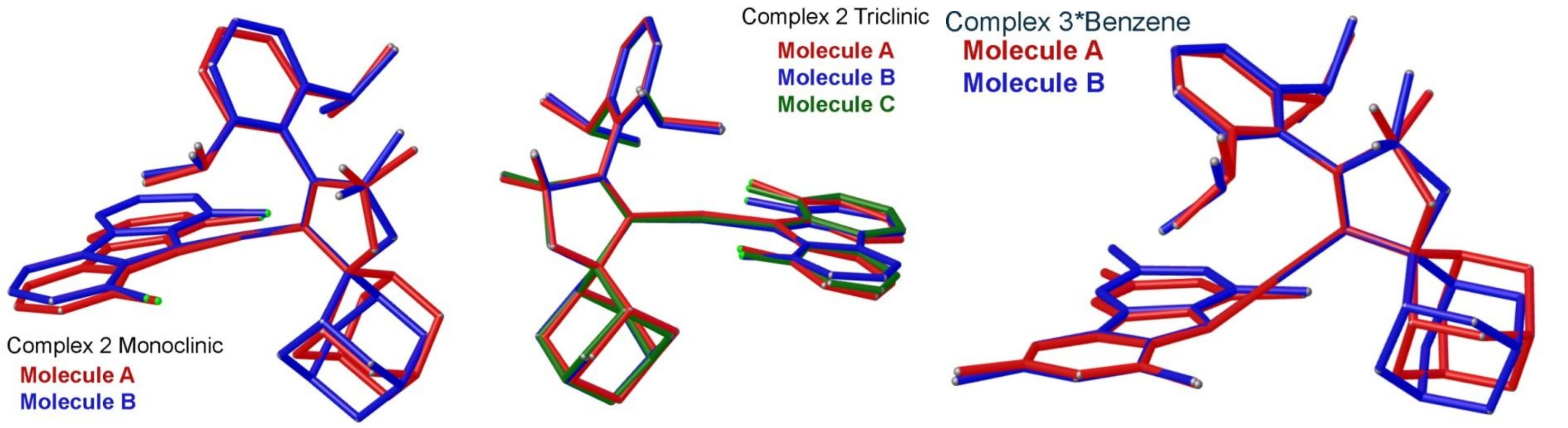
Complex 2 Monoclinic
Molecule A
Molecule B
Complex 2 Triclinic
Molecule A
Molecule B
Molecule C
Complex 3*Benzene
Molecule A
Molecule B

b)

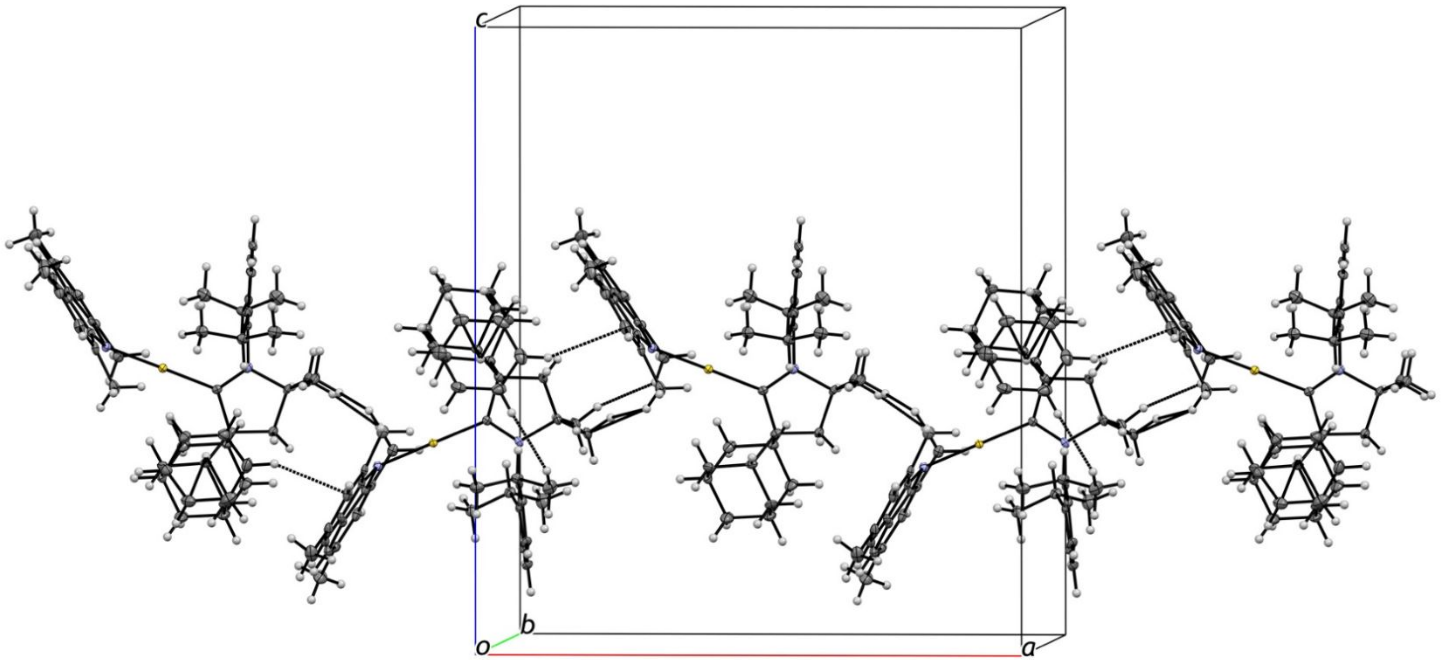
c
b
o
a

c)

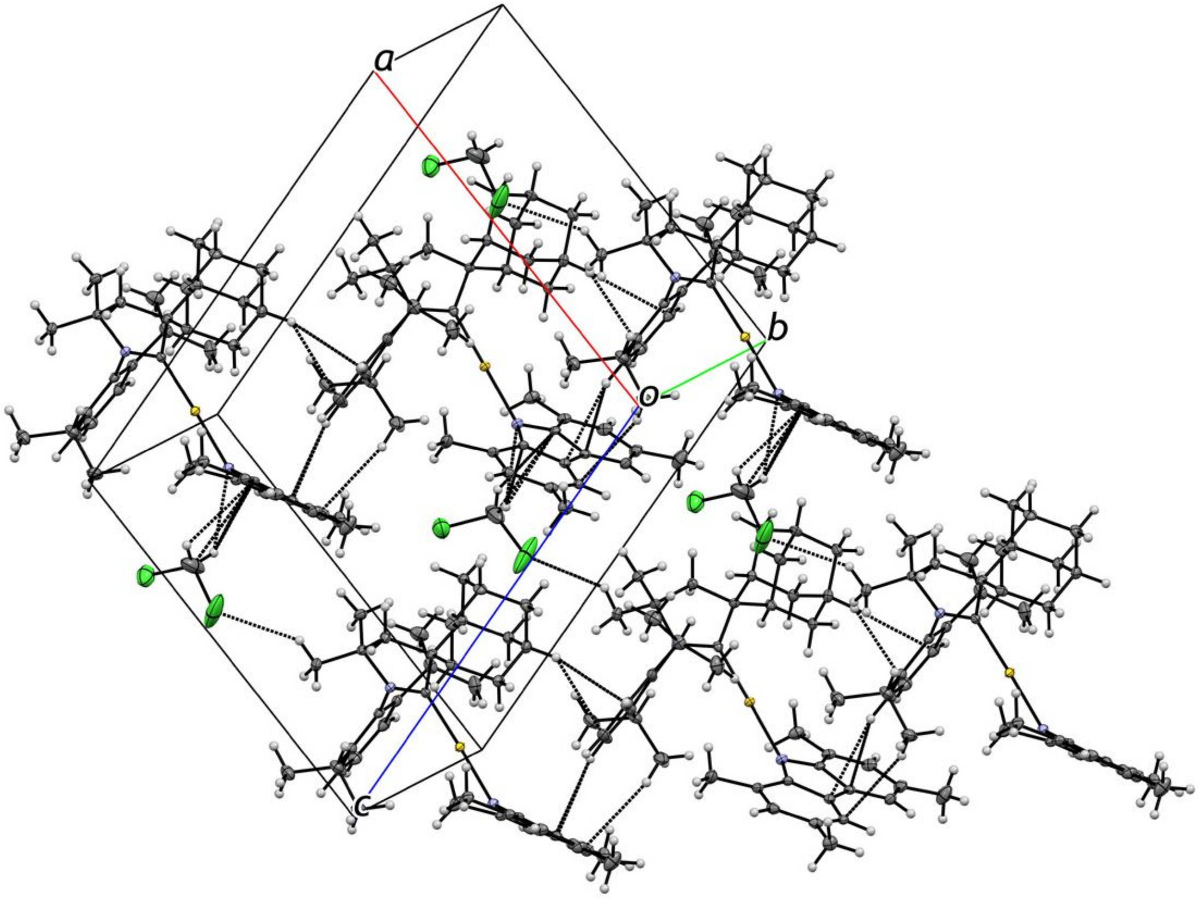

Figure S7. a) Overlap of independent molecules A, B and C for complexes **2** (Triclinic, P-1), **2** (Monoclinic, P2$_1$/c) and **3**·Benzene. b) Packing diagram for **3·benzene** (top); c) Packing diagram for **3·CH$_2$Cl$_2$** (bottom) complexes.

Thin-film preparation

The solid-state samples for steady-state UV-Vis, photoluminescence (PL) and time-resolved PL measurements were prepared on the pre-cleaned Quartz substrates. Solution-processed complex **3** and **4** were spin-coated inside a solvent glovebox from anhydrous chlorobenzene solutions in either pristine or blended conditions. The samples were then annealed in the hot plate at 90°C for 10 minutes to remove the remaining solvent. Vacuum-deposited pristine or doped thin films for complex **1** and **2** were prepared by thermal evaporation under high vacuum (10^{-7} torr). The thickness of all the thin-films is ca. 80–100 nm.

Photophysical Characterisation

Solution UV-visible absorption spectra were recorded using a Perkin-Elmer Lambda 35 UV/vis spectrometer. UV-vis spectra of solid films were recorded using an Agilent 8453 UV-visible spectrophotometer. Photoluminescence measurements for solid state, host-guest films, MeTHF solutions at 298 and 77K were recorded using an Edinburgh Instruments FLS980 and FS5 spectrometers. Absoulte photoluminescent quantum yields was measured for toluene solutions using an Edinburgh Instruments FS5 spectrometer with excitation wavelength 400 nm for complex **1** (0.02 mg mL^{-1}), with 360 nm for **2** (0.02 mg mL^{-1}), with 415 nm for complex **3** (0.05 mg mL^{-1}), and 415 nm for complex **4** (0.03 mg mL^{-1}). Toluene solutions have been prepared in a nitrogen glovebox from freshly distilled toluene and measured in a 1 cm screw-cap quartz cuvette.

All excited state lifetimes for complexes **1–4** were measured on Edinburgh Instruments FS5 spectrometer with mono- and biexponential fitting provided by Edinburgh Instruments Fluoracle software v2.6.1. The crystalline, solution, neat film and host-guest time resolved fluorescence data at 77K were collected on an Edinburgh Instruments FS5 spectrofluorimeter using the 5 W microsecond Xe flash lamp with a repetition rate of 100 Hz (360 nm excitation wavelength).

Temperature-dependent time-resolved photoluminescence measurements (cryogenic PL measurement)

Solid-state samples were prepared using the same method as thin-film preparation for steady-state measurement. Time-resolved PL spectra were carried out by using an electrically-gated intensified CCD (ICCD) camera (AndoriStar DH740 CCI-010) coupled with an image identifier

tube, giving high sensitivity and low noise level. A calibrated grating spectrometer (Andor SR303i) is connected to the ICCD for spectrum calibration. Photoexcitation is at the wavelength of 400 nm, given by femtosecond laser pulses which were created by second harmonic generation (SHG) in a BBO crystal from the fundamental 800 nm output (pulse width = 80 fs) of a Ti:Sapphire laser system (Spectra Physics Solstice), at a repetition rate of 1 kHz and ca. 200 µm diameter spotsize on a sample. A 425 nm long-pass filter was applied to avoid the scattered laser signals entering the camera. The minimum gate width of the ICCD was ca. 5 ns. The kinetics of PL emissions can be obtained by setting the gate delay steps with respect to the excitation pulse. The gate width used in the measumets was 200µm. The iCCD gate duration was 5 ns for 0-100 ns region, 10 ns for 100-1000ns, 1µs for 1-100µs region and 10µs for 100-900µs. Liquid helium was used for sample cooling and a temperature-controlled cryostat was used for temperature regulation.

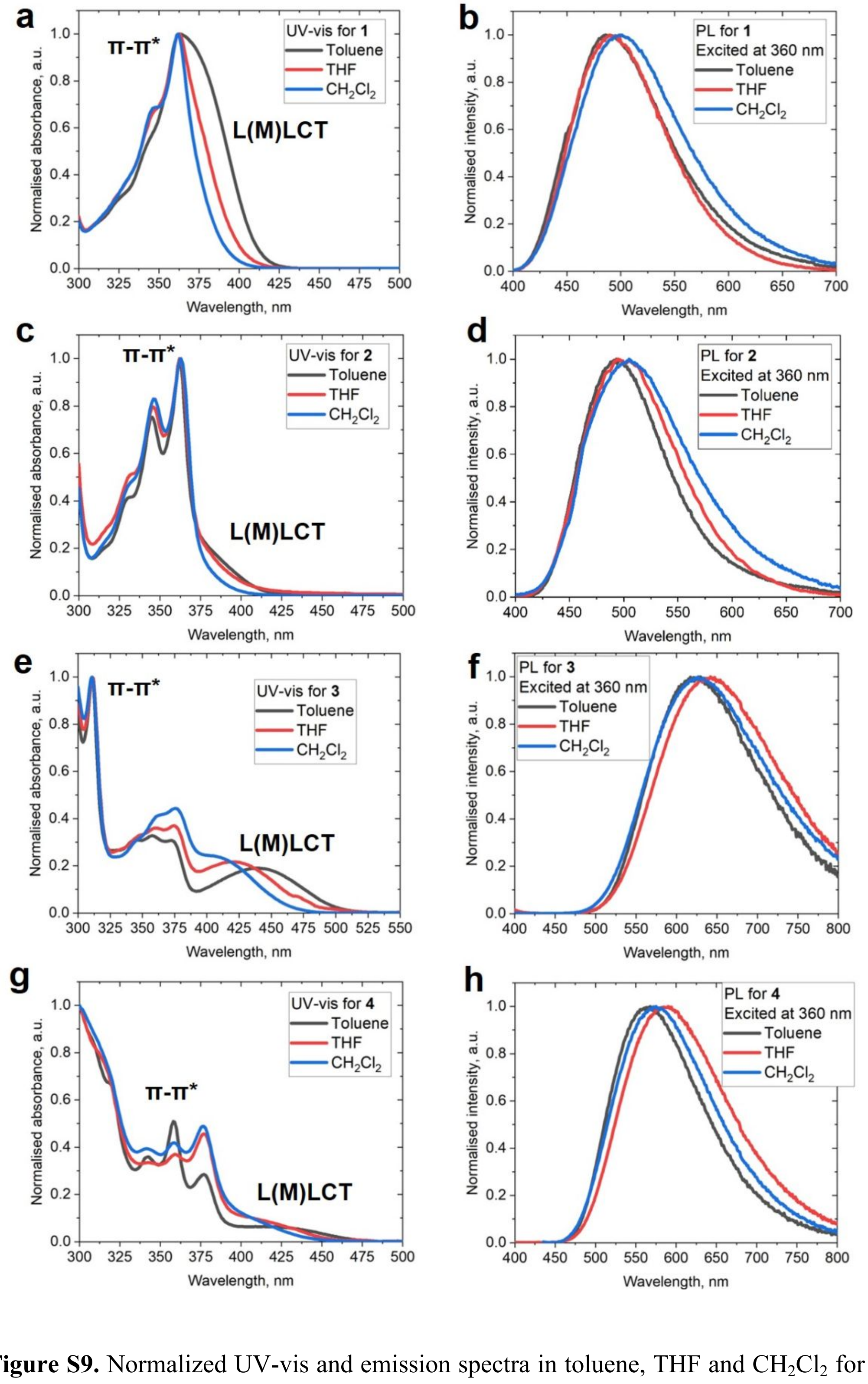

Figure S9. Normalized UV-vis and emission spectra in toluene, THF and CH_2Cl_2 for complexes **1** (a, b), **2** (c, d), **3** (e, f) and **4** (g, h) showing negative and positive solvatochromism, respectively.

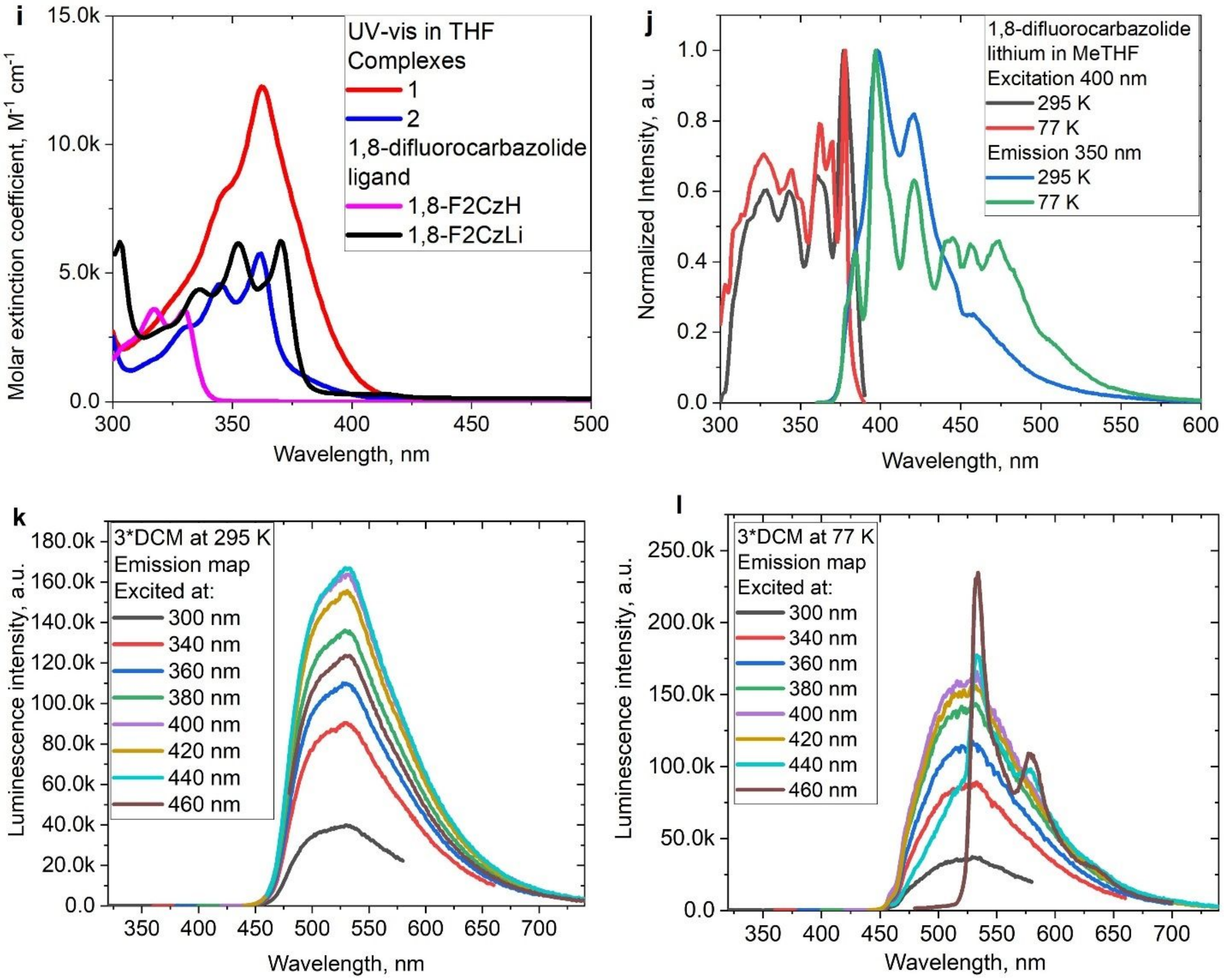

Figure S9. Normalized UV-vis and emission spectra in THF for complexes **1**, **2** and ligands 1,8-difluorocarbazole and corresponding lithium salt (i, j); emission map for complex 3*DCM at room temperature (k) and 77 K (l).

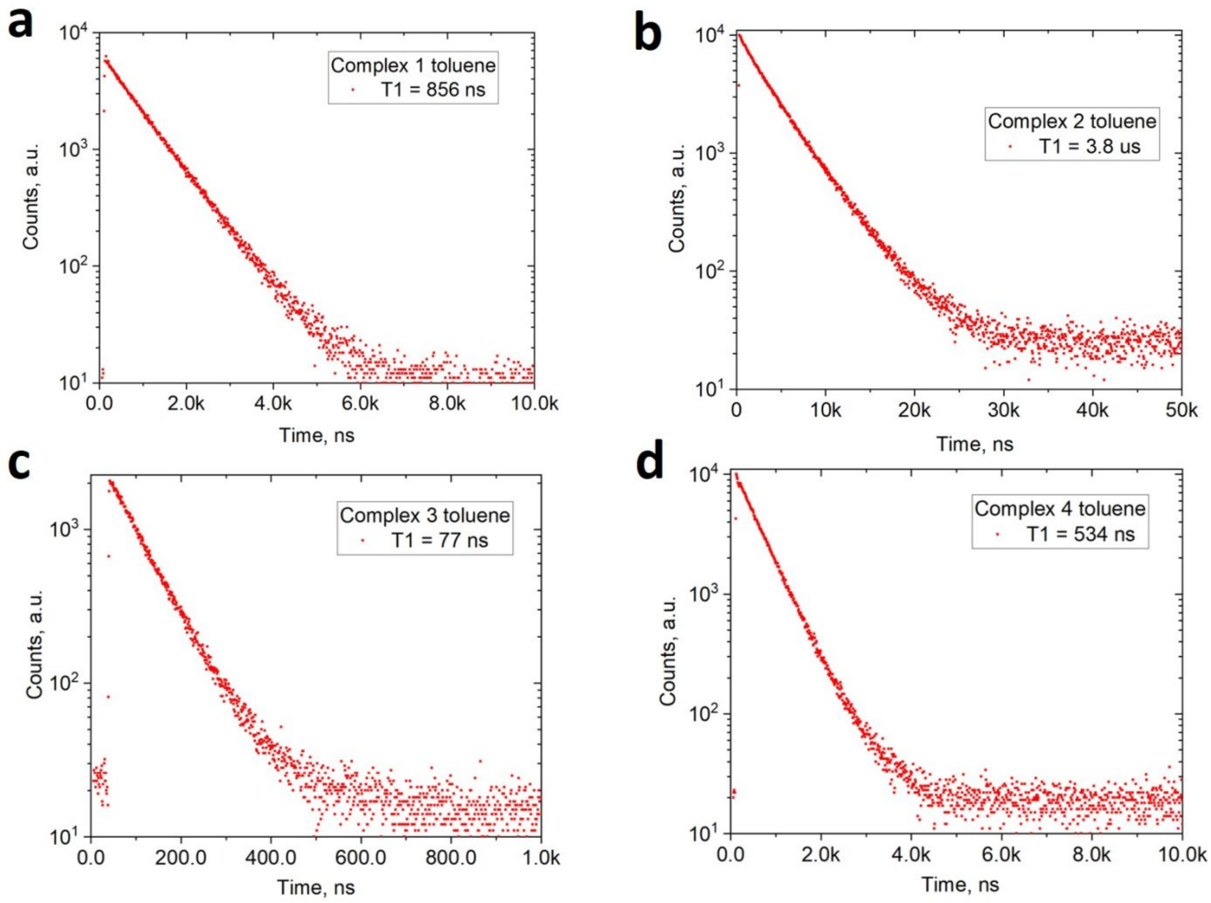

Figure S10. Emission decay kinetics for complex **1 (a), 2 (b), 3 (c)** and **4 (d)** in toluene solution at 295 K.

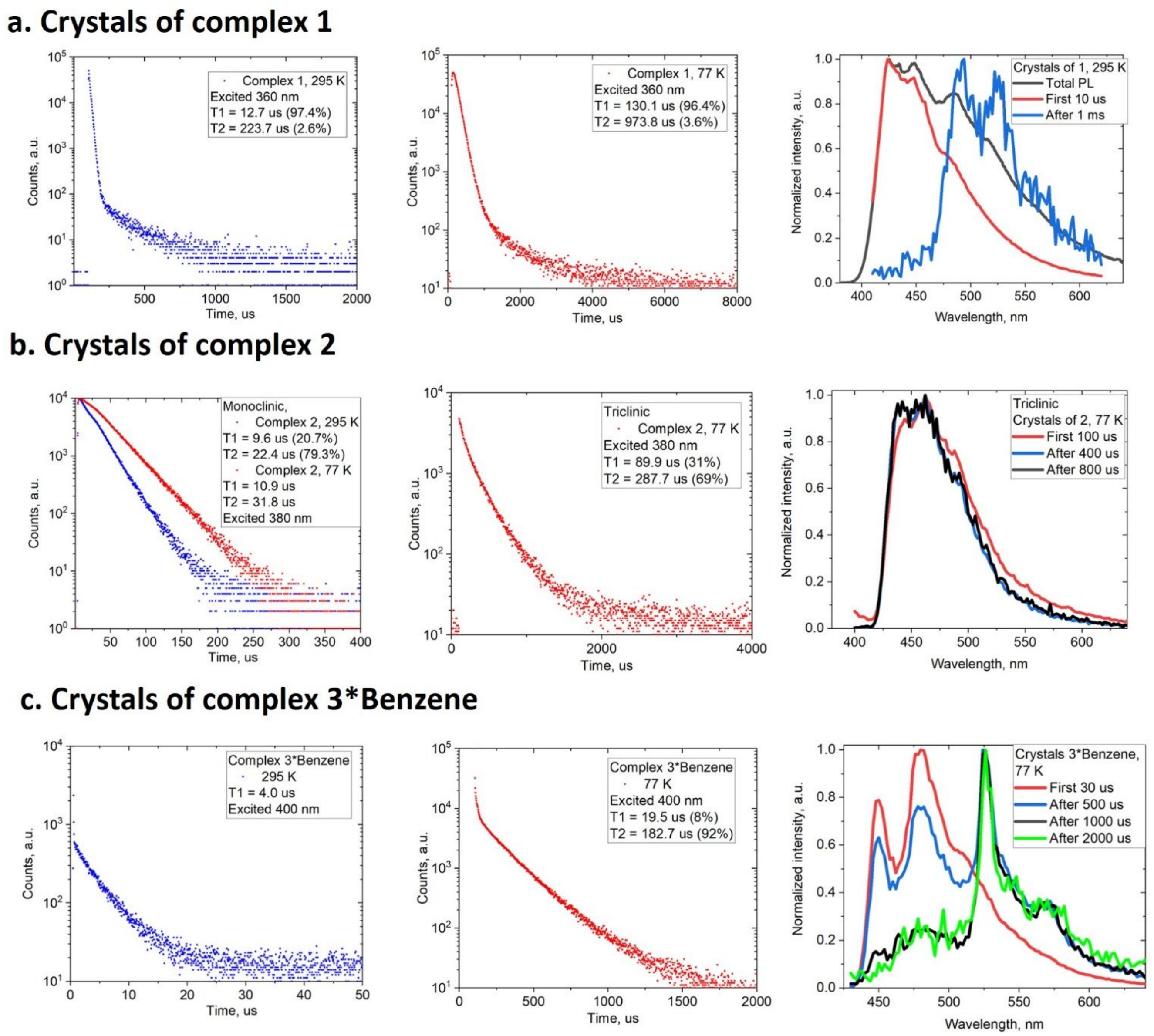

Figure S11 Emission decay kinetics for crystals of complexes **1**, **2** and **3** at 295 K (left panel), 77 K (middle), and photoluminescence profile after various delays at 77 K (right panel).

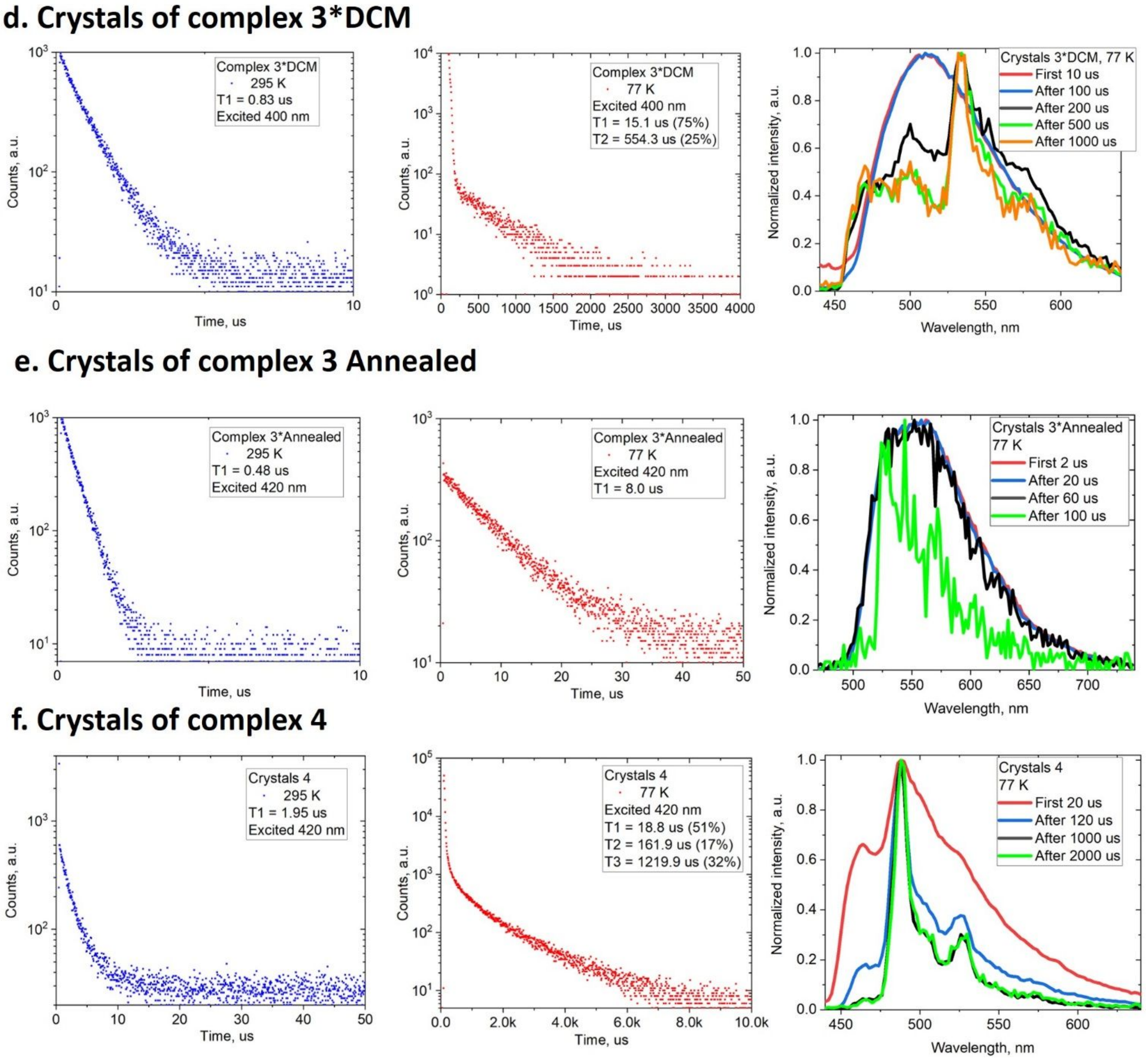

Figure S11 Continue. Emission decay kinetics for crystals of complexes **3** and **4** at 295 K (left panel), 77 K (middle), and photoluminescence profile after various delays at 77 K (right panel).

Fitting and calculation of the activation energy for reverse intersystem crosing (rISC), radiative (k_r) and non-radiative decay rate (k_{nr})

As the non-radiative decay is no longer negligible for twisted/tilted complexes, we assume it follows the same rate equation of activation energy[7]

$$k_r = A_1 \times \exp\left(\frac{\Delta E_{A,r}}{k_B T}\right) + y_0 \quad \textbf{(S1)}$$

$$k_{nr} = A_2 \times \exp\left(\frac{\Delta E_{A,nr}}{k_B T}\right) + y_1 \quad \textbf{(S2)}$$

Where $\Delta E_{A,r}$ and $\Delta E_{A,nr}$ are the activation energy for radiative decay and non-radiative decay respectively. $\Delta E_{A,r}$ is the activation energy for reverse intersystem crosing (rISC) from triplets to singlets. Both decay rates are related to the total PL intensity (PL_{tol}) and the absolute decay rate (k_T) with the following relationships:

$$k_T = k_r + k_{nr} \quad \textbf{(S3)}$$

$$PL_{tol} \propto \frac{k_r}{k_r + k_{nr}} \quad \textbf{(S4)}$$

Where PL_{tol} is the temperature dependent total PL intensities calculated by integrating the time-dependent decays over the complete measurement window and k_T is the reciprocal of the corresponded decay time when the normalized PL integral equals to $\frac{1}{e}$.

The equations **S3** and **S4** were adopted as the constraints to get all the values (A_1, A_2, $\Delta E_{A,r}$, $\Delta E_{A,nr}$, y_0 and y_1) in equations **S1** and **S2**. Because the non-radiative decay process becomes significant only at high temperature, below that temperature T_1, PL_{tol} and k_T increase with temperature. Consequently, the value of $\Delta E_{A,r}$ can be calculated by fitting the curve of k_T VS. Temperature from 10 K to K_1.

We take complex **3** as the example to demonstrate the fitting process:

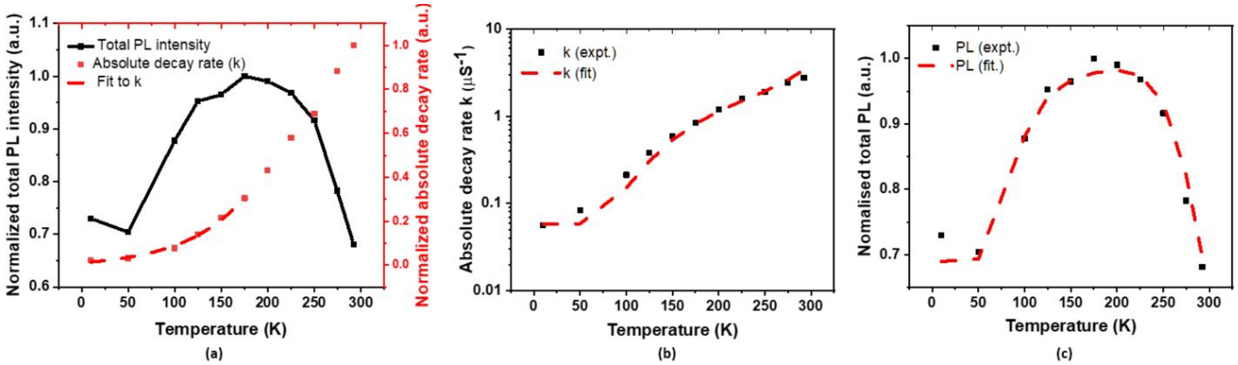

Figure S12. (a) Total PL intensity (black dots) and absolute decay rate (red dots) obtained from temperature-dependent time-resolved PL spectra by using an electrically gated ICCD, the red dash line is the fitting to the decay rate from 10 K to 175 K by using the rate equation; (b) experimental absolute decay rate k_T and fitted k_T using equation **S3**; (c) experimental total PL intensity PL_{tol} and fitted PL_{tol} using equation **S4**.

The total PL intensity begins to drop from 175 K, so we only fit the data of PL_{tol} and k_T below 175 K by using the rate equation (S1). The calculated average activation energy is 42.2 meV and we substitute this value as $\Delta E_{A,r}$ in equation S1. By fitting the PL_{tol} and k_T among the whole measurement window (10K to 100K) and applying equations **S3** and **S4**, we obtained the optimised values for other constants in equations **S1** and **S2** and collected in Table S1.

Table S1. Optimized values for the rate equations of complexes **1–4**.

	Complex **1**	Complex **2**	Complex **3**	Complex **4**
$\boldsymbol{A_1}$	1.00×10^{-2}	4.00×10^{-3}	1.25×10^{-2}	9.40×10^{-3}
$\boldsymbol{\Delta E_{A,r}}$	58.11	136.40	42.22	41.80
$\boldsymbol{y_0}$	4.00×10^{-5}	2.00×10^{-5}	4.00×10^{-5}	3.00×10^{-5}
$\boldsymbol{A_2}$	-	600.00	450.00	520.00
$\boldsymbol{\Delta E_{A,nr}}$	-	323.13	327.45	344.68
$\boldsymbol{y_1}$	-	5.00×10^{-4}	1.80×10^{-5}	1.00×10^{-5}

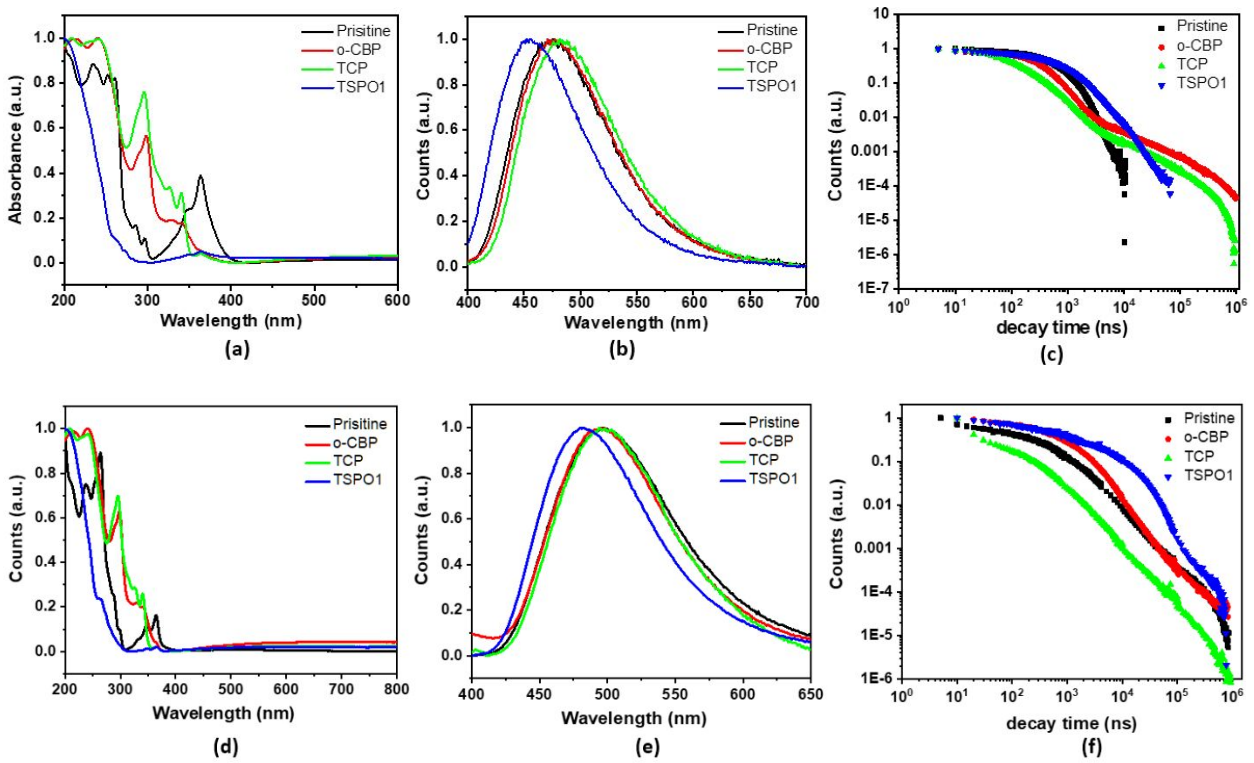

Figure S13. UV-Vis absorption spectra **(a) (d)**, steady-state PL spectra **(b) (e)** and time-resolved PL decay at room temperature **(c) (f)** of thin-film pristine complexes **1** and **2**, and 20% doped in o-CBP, TCP and TSPO1. Thin films were deposited via vacuum-deposition.

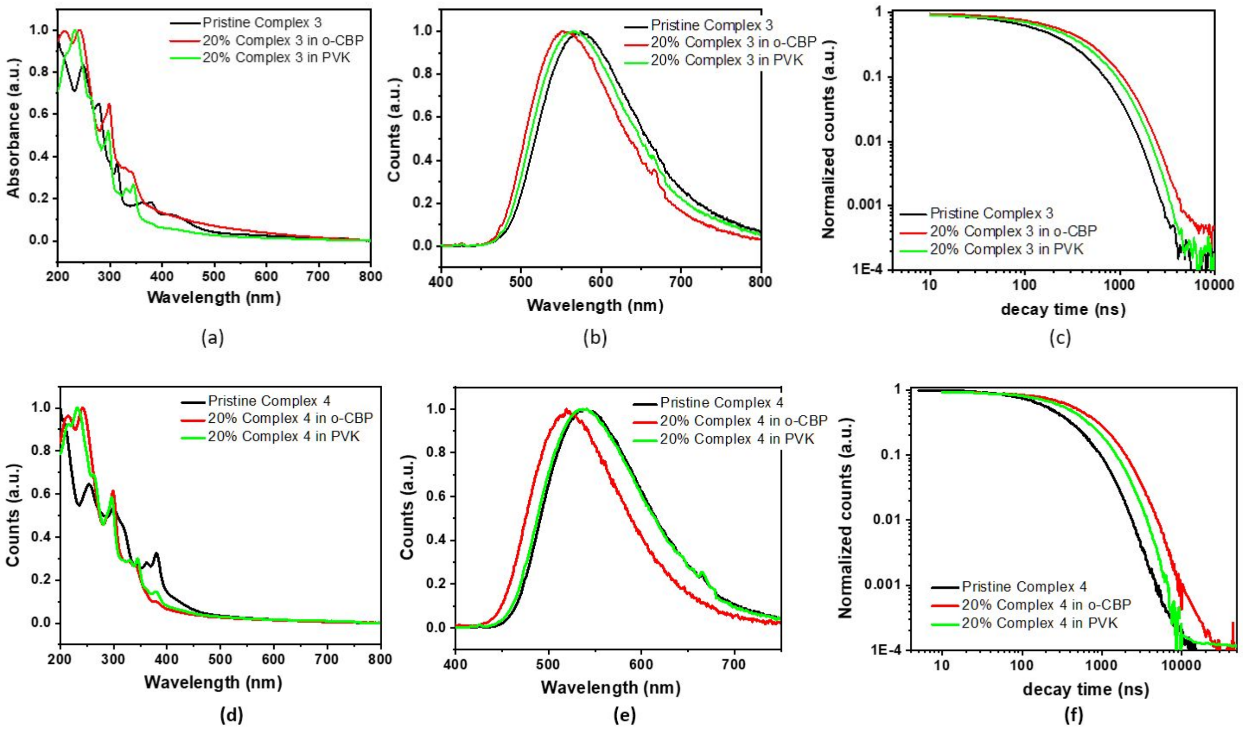

Figure S14. UV-Vis absorption spectra **(a) (d)**, steady-state PL spectra **(b) (e)** and time-resolved PL spectra at room temperature **(c) (f)** of thin-film Pristine Complexes **3** and **4**, and 20% doped in o-CBP and PVK. Thin films were spin-coated from chlorobenzene solutions.

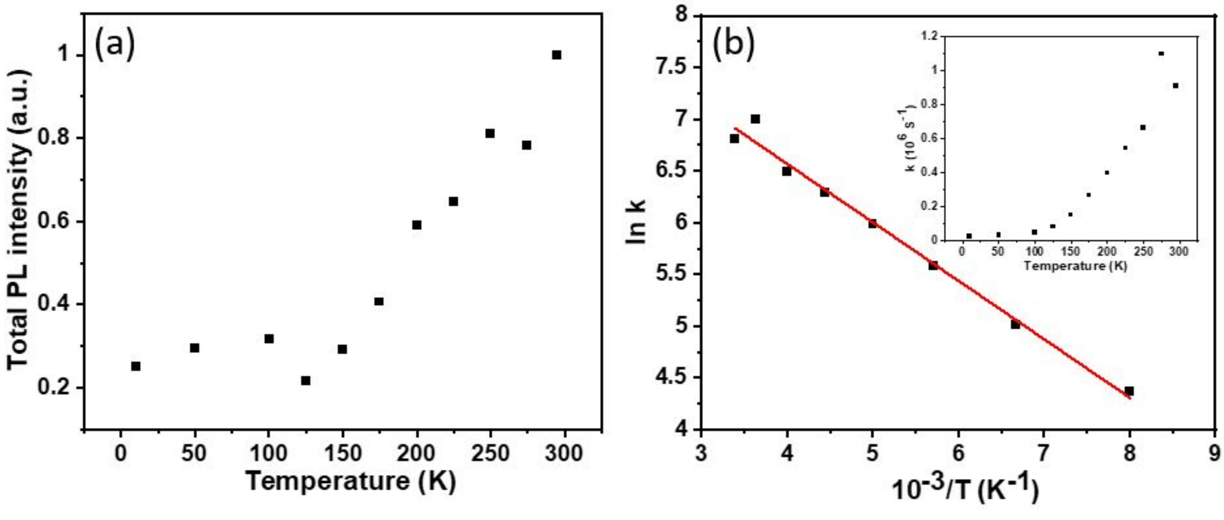

Figure S15. (a) Complex **1** temperature-dependent total PL intensities calculated by integrating the time-dependent decays over the complete measurement window; (b) Temperature-dependent decay rate k and the fitting of ln (k) to 1/T.

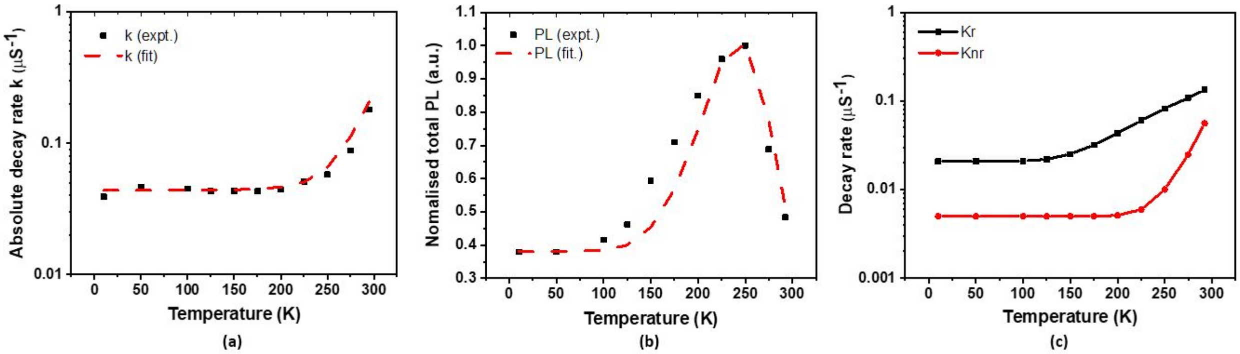

Figure S16. (a) Absolute decay rate K extracted from temperature-dependent time-resolved PL spectra and fitted decay rate of complex **2**; (b) Normalised total PL intensity extracted from temperature-dependent time-resolved PL spectra and fitted total PL of complex **2**; (c) Calculated radiative decay rate K_r and non-radiative decay rate K_{nr} or complex **2**.

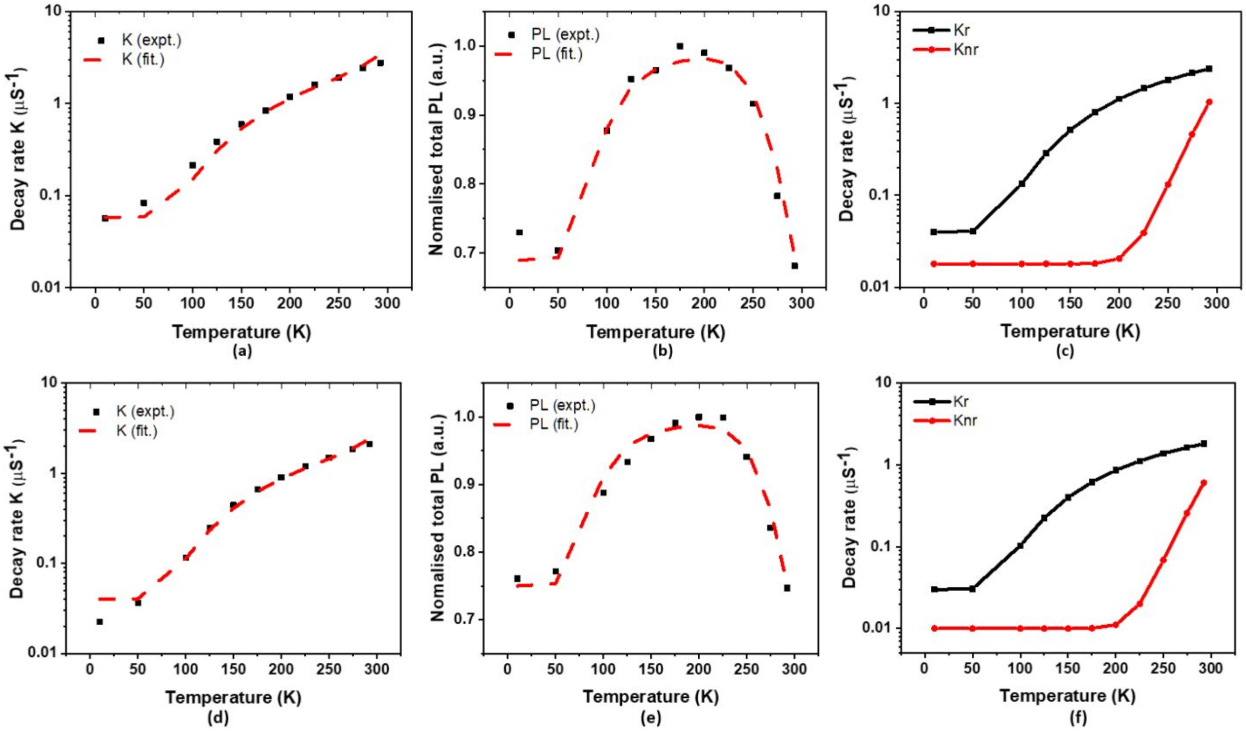

Figure S17. (a) (d) Absolute decay rate K extracted from temperature-dependent time-resolved PL spectra and fitted decay rate of complex **3** and **4**; (b) (e) Normalised total PL intensity extracted from temperature-dependent time-resolved PL spectra and fitted total PL of complex **3** and **4**; (c) (f) Calculated radiative decay rate K_r and non-radiative decay rate K_{nr} or complex **3** and **4**.

Table S2. Steady-state PL peak, decay lifetime and FWHM of solid-state pristine complexes **3** and **4** or 20% doped in o-CBP and PVK.

	Complex 3			Complex 4		
	λ_{em}	τ (μs)	FWHM (nm)	λ_{em}	τ (μs)	FWHM (nm)
**Pristine**	574	0.34	139	535	0.47	126
**o-CBP**	551	0.50	130	520	0.56	108
**PVK**	561	0.45	137	535	0.65	127

Solution-processing device fabrication

Devices were carried out by solution processing with a forward configuration as follows: ITO/PEDOT:PSS/Active Layer/ETL/LiF/Al. Indium tin-oxide (ITO; WF ~ 4.8 eV) coated glass substrates were used and subsequently cleaned by sonication in acetone and 2-propanol for 10 minutes, followed by O_2 plasma treatment for 10 minutes. PEDOT: PSS (Clevios CH4083, LumTech Taiwan) was spin-casted on top of the ITO under ambient conditions and annealed on a hot plate at 160 °C for 20 minutes, forming a 40 nm-thick film. The PEDOT: PSS-coated substrates were then transferred to a nitrogen-filled glovebox to conduct the following solution processes.

For structure **1**, first a 40 nm-thick emitting layer (EML) of o-CBP doped with 20 wt.% of complex **3** and **4** in chlorobenzene was spin-coated on top of the PEDOT: PSS layer and annealed on a hot plate at 60 °C for 5 min to remove residual solvent. Since o-CBP is sensitive to humidity and temperature, the glovebox atmosphere has to be stable to avoid crystallization. Samples were then transferred to a vacuum deposition system. 10 nm of TSPO1 doped with 10 wt.% of 1,3,5-tris(2-N-phenylbenzimidazole-1-yl)benzene (TPBi) was deposited, followed by 40 nm-thick of TSPO1 as the electron-transporting layer (ETL). For structure 2, instead, 10 nm-thick of UGH2: TPBi (9:1 in wt.%) and 40 nm-thick of TPBi were used as the hole blocking layer (HBL) and the ETL.

Similarly, for structure **3**, 50 nm-thick EML of Poly(9-vinylcarbazone) (PVK, Sigma-Aldrich) doped with 20 wt.% of complex **3** and **4** in chlorobenzene was spin-coated on top of the PEDOT: PSS and annealed at 90°C for 10 min to remove any solvent present. Samples were then transferred to the vacuum deposition system for further deposition of 10 nm-thick of UGH2: TPBi (9:1 in wt.%) and 40 nm-thick of TPBi. For all samples, 0.8 nm-thick LiF (99.99%, Sigma-Aldrich) and 100 nm-thick aluminium were subsequently deposited by thermal evaporation under high vacuum (< 3×10^{-6} mbar) as the electrode.

Vacuum thermal-evaporated device fabrication

Fully-evaporated devices were fabricated by high vacuum (10^{-7} Torr) thermal evaporation on ITO glass substrates. The optimum blue architecture for complex **1** and **2** devices comprised a 40 nm thick layer of 1,1-bis[4-[N,N-di(4-tolyl)amino]phenyl]-cyclohexane (TAPC) used as the electron blocking layer (EBL) and HTL. A 5 nm-thick of 9,9'-Biphenly-2,2'-diylbis-9H-carbazole (o-CBP) was deposited as an additional EBL to avoid triplet transfer towards anode or the formation of interfacial states between EML and HTL. The following layer is a 30 nm-thick of EML comprising either host free or 20 wt.% of complex **1** and **2** dispersed in a host material. o-CBP, 1,3,5-tris(carbazol-9-yl)benzene (TCP) and diphenyl-4-triphenylsilyl-phenylphosphine oxide (TSPO1) were used as the host materials. Then, a 40 nm layer of TSPO1 acting as the ETL and HBL was evaporated.

Another OLED architecture for complex **1** and **2** used TAPC as the EBL and HTL. A 20 nm of either host-free or 20 wt. % doped complex **1** and **2** in various host materials were evaporated on top of the TAPC. Same hosts were tested (i.e. o-CBP, TCP and TSPO1). A 10 nm of 1,4-bis(triphenylsilyl)benzene (UGH2) was deposited as the EBL. 40 nm-thick layer of TPBi was then evaporated as the ETL. For both structures, a 0.8 nm-thick LiF (99.99%, Sigma-Aldrich) and a 100 nm-thick aluminium were subsequently deposited as the cathode. Each substrate has eight 4.5 mm^2 pixels as defined by the overlap area of the shadow mask and ITO stripe.

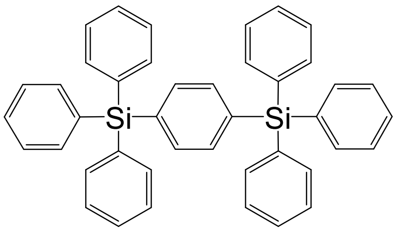

UGH2

OLED Characterisation.

The forward-viewing current–voltage–luminance characteristics of these OLED devices were measured using a Keithley 2400 source meter, Keithley 2000 multimeter and a calibrated Si photodiode (from RS components), which was placed at a distance of 4 cm from the devices. External quantum efficiencies were calculated from on-axis irradiance assuming a Lambertian emission profile and accounting for photodiode quantum efficiency across the electroluminescence spectrum. The electroluminescence spectra were obtained by a fibre spectrometer (Flame-S-VIS-NIR-ES, Ocean Optics). All the measurements were carried out at room temperature under ambient conditions.

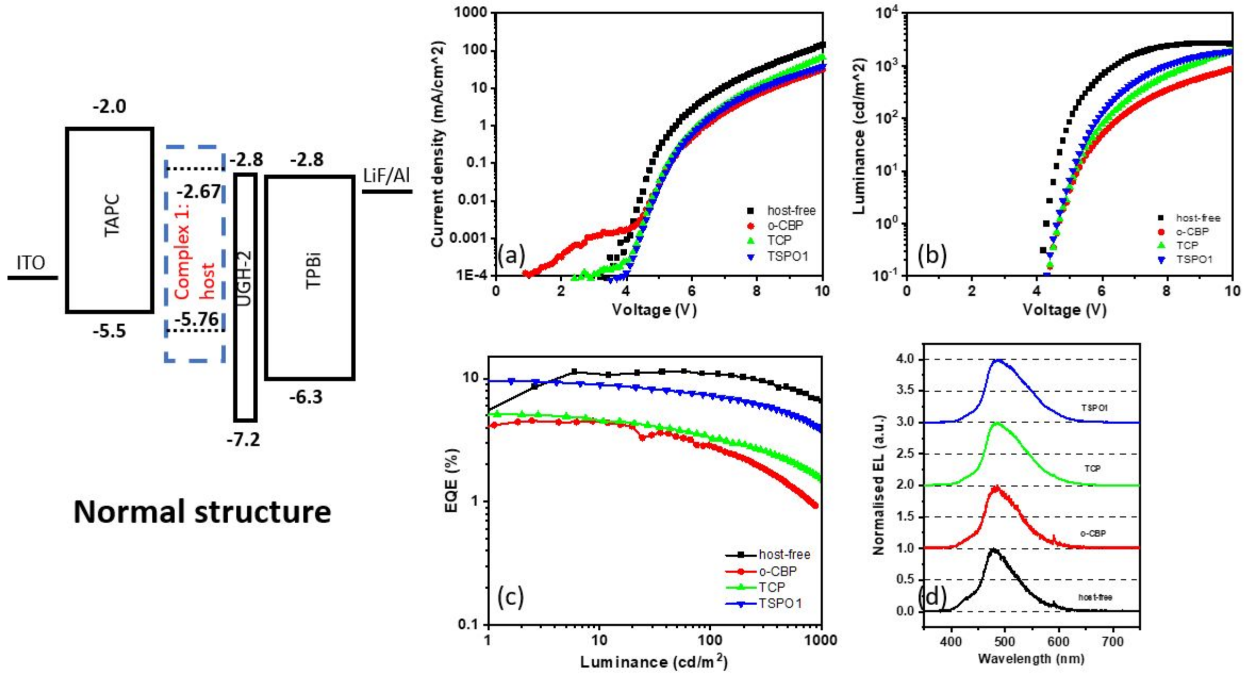

Figure S18. Typical OLED performance of complex **1** in host free and different hosts in a normal structure (left); **(a)** Current-density vs. Voltage; **(b)** Luminance vs. Voltage; **(c)** external quantum efficiency (EQE) vs. luminance; **(d)** electroluminescence (EL) spectra.

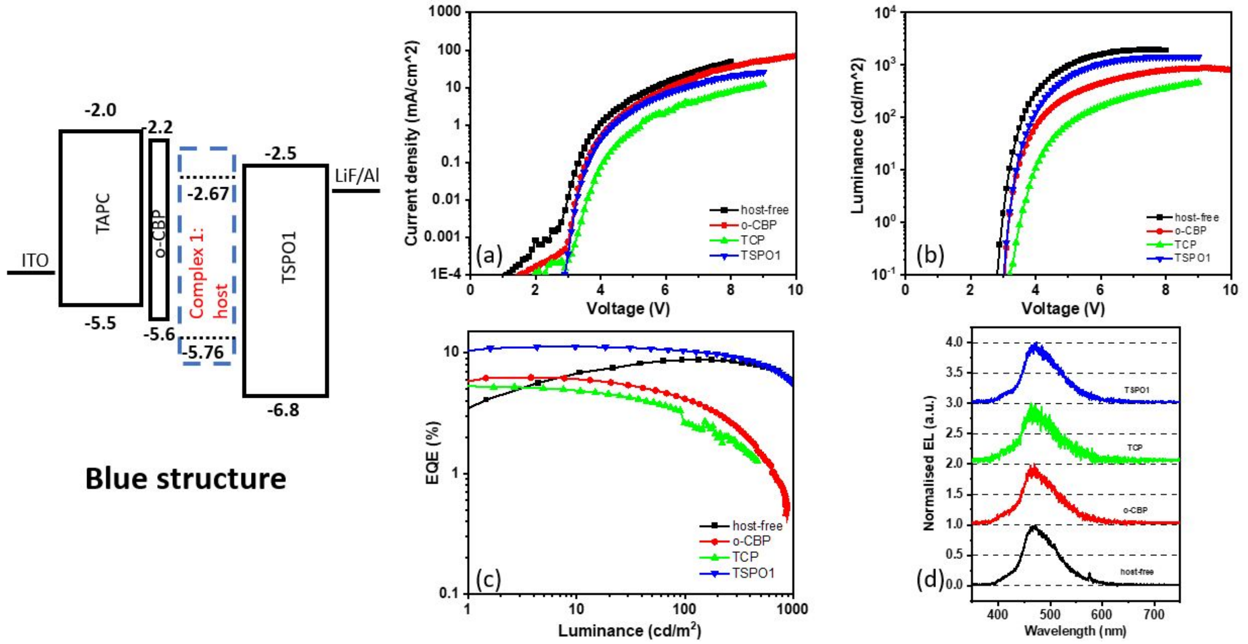

Figure S19. Typical OLED performance of complex **1** in host free and different hosts in a blue structure (left); **(a)** Current-density vs. Voltage; **(b)** Luminance vs. Voltage; **(c)** external quantum efficiency (EQE) vs. luminance; **(d)** electroluminescence (EL) spectra.

Table S3. OLED performance summary of complex **1**

	Normal architecture				
	Turn-on (V)	EQE (max. %)	EQE % (100 cd/m²)	EQE % (1000 cd/m²)	EL peaks (nm)
Host-free	4.4	11.5	11.0	6.6	485
o-CBP	4.7	6.8	4.2	1.2	485
TCP	4.7	5.2	3.5	1.5	485
TSPO1	4.7	9.6	7.4	3.9	480

	Blue architecture				
	Turn-on (V)	EQE (max. %)	EQE % (100 cd/m²)	EQE % (1000 cd/m²)	EL peaks (nm)
Host-free	3.0	8.8	8.7	6.0	480
o-CBP	3.2	6.3	4.2	-	480
TCP	3.6	5.3	2.6	-	480
TSPO1	3.2	11.3	10.5	5.9	475

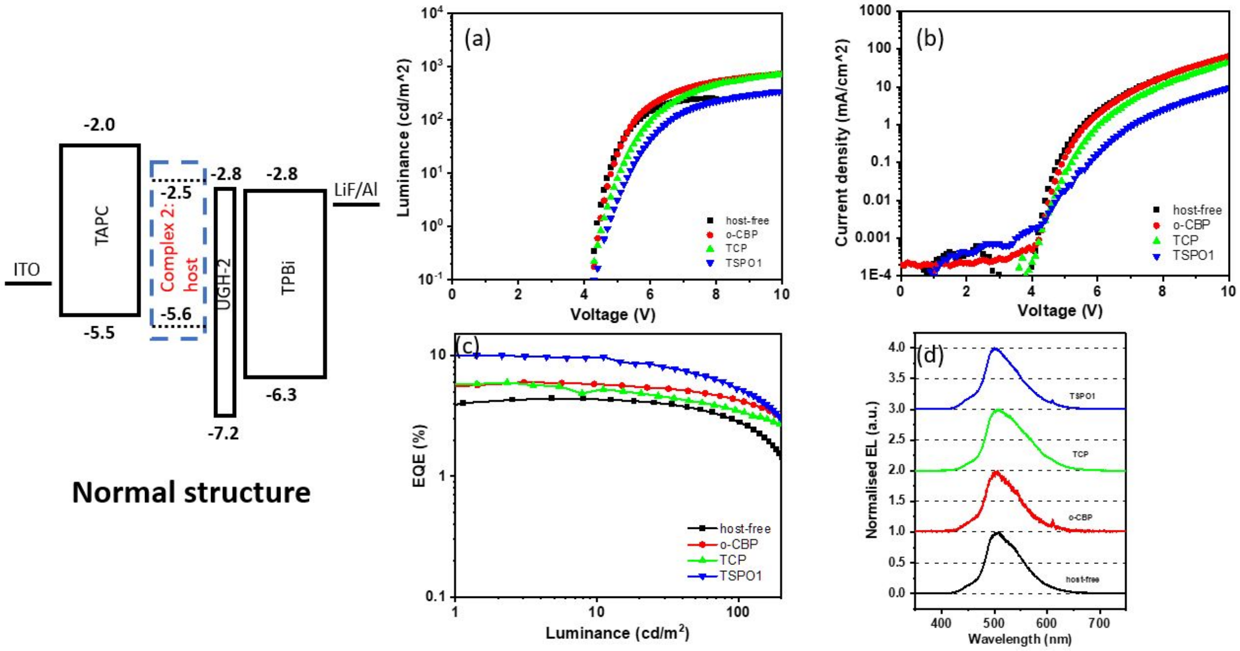

Figure S20. Typical OLED performance of complex **2** in host free and different hosts in a normal structure (left); **(a)** Current-density vs. voltage; **(b)** Luminance vs. voltage; **(c)** external quantum efficiency (EQE) vs. luminance; **(d)** electroluminescence (EL) spectra.

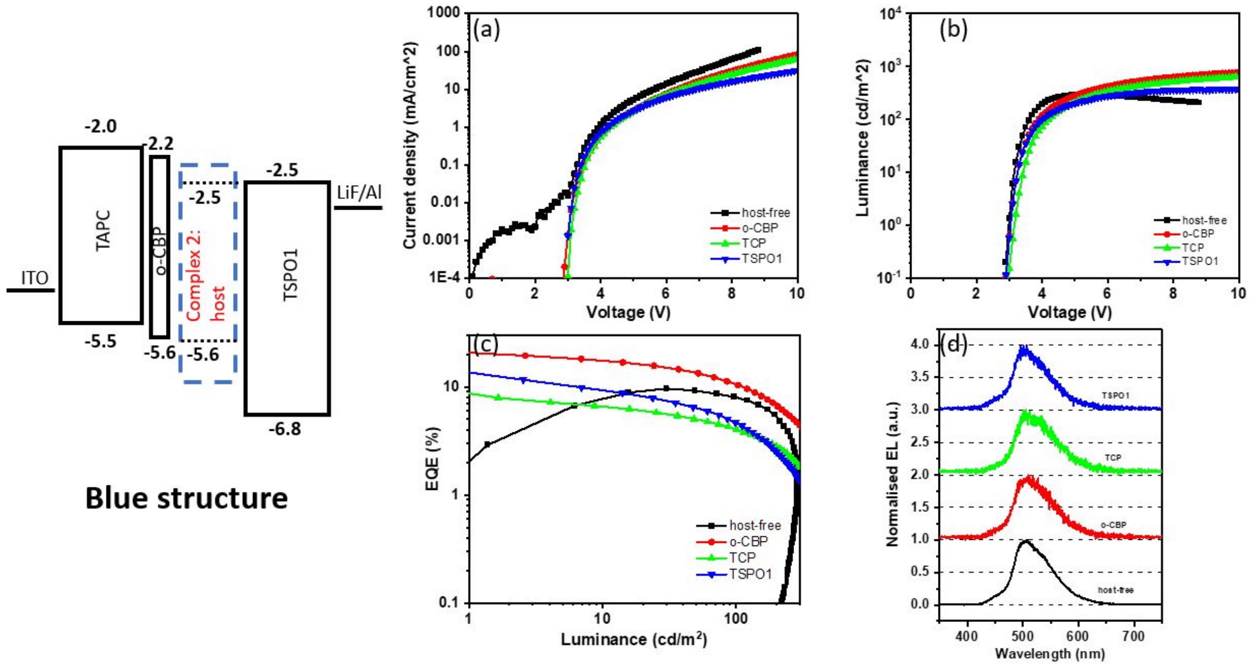

Figure S21. Typical OLED performance of complex **2** in host free and different hosts in a blue structure (left); **(a)** current-density vs. voltage; **(b)** luminance vs. voltage; **(c)** external quantum efficiency (EQE) vs. luminance; **(d)** electroluminescence (EL) spectra.

Table S4. OLED performance summary of complex **2**

	Normal architecture				
	Turn-on (V)	EQE (max. %)	EQE % (100 cd/m²)	EQE % (1000 cd/m²)	EL peaks (nm)
o-CBP	4.5	7.3	4.4	-	495
TSPO1	4.8	10.5	6.4	-	492
TCP	4.5	6.0	3.5	-	495
Host-free	4.4	4.5	2.9	-	495

	Blue architecture				
	Turn-on (V)	EQE (max. %)	EQE % (100 cd/m²)	EQE % (1000 cd/m²)	EL peaks (nm)
o-CBP	3.2	21.3	10.6	-	500
TSPO1	3.1	13.2	5.0	-	496
TCP	3.2	9.3	4.2		500
Host-free	3.0	9.6	7.9	-	498

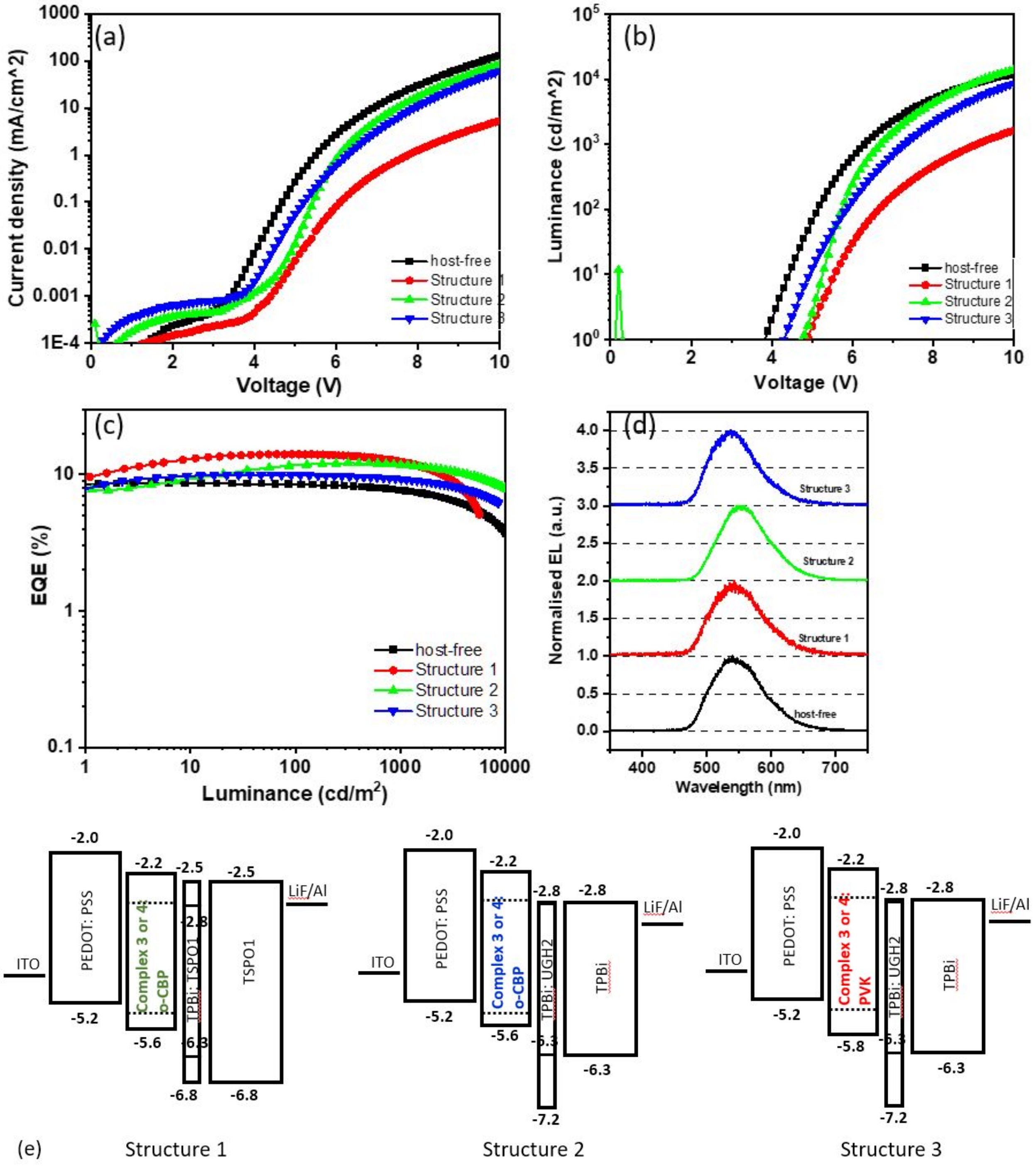

Figure S22. Typical OLED performance with complex **3** in host-free and host-guest stack; **(a)** current-density vs. voltage; **(b)** luminance vs. voltage; **(c)** external quantum efficiency (EQE) vs. luminance; **(d)** electroluminescence (EL) spectra; **(e)** Schematic of OLED stack of structure 1, 2 and 3 for Complex **3** and **4**, host-free is in structure 1.

Table S5. OLED performance summary of complex **3**

	Normal architecture				
	Turn-on (V)	EQE (max. %)	EQE % (100 cd/m²)	EQE % (1000 cd/m²)	EL peaks (nm)
**Host-free**	5.2	10.0	10.0	9.2	550
**Structure 1**	4.3	14.1	14.1	12.5	540
**Structure 2**	4.8	12.1	11.6	11.8	540
**Structure 3**	4.4	10.2	10.1	9.5	550

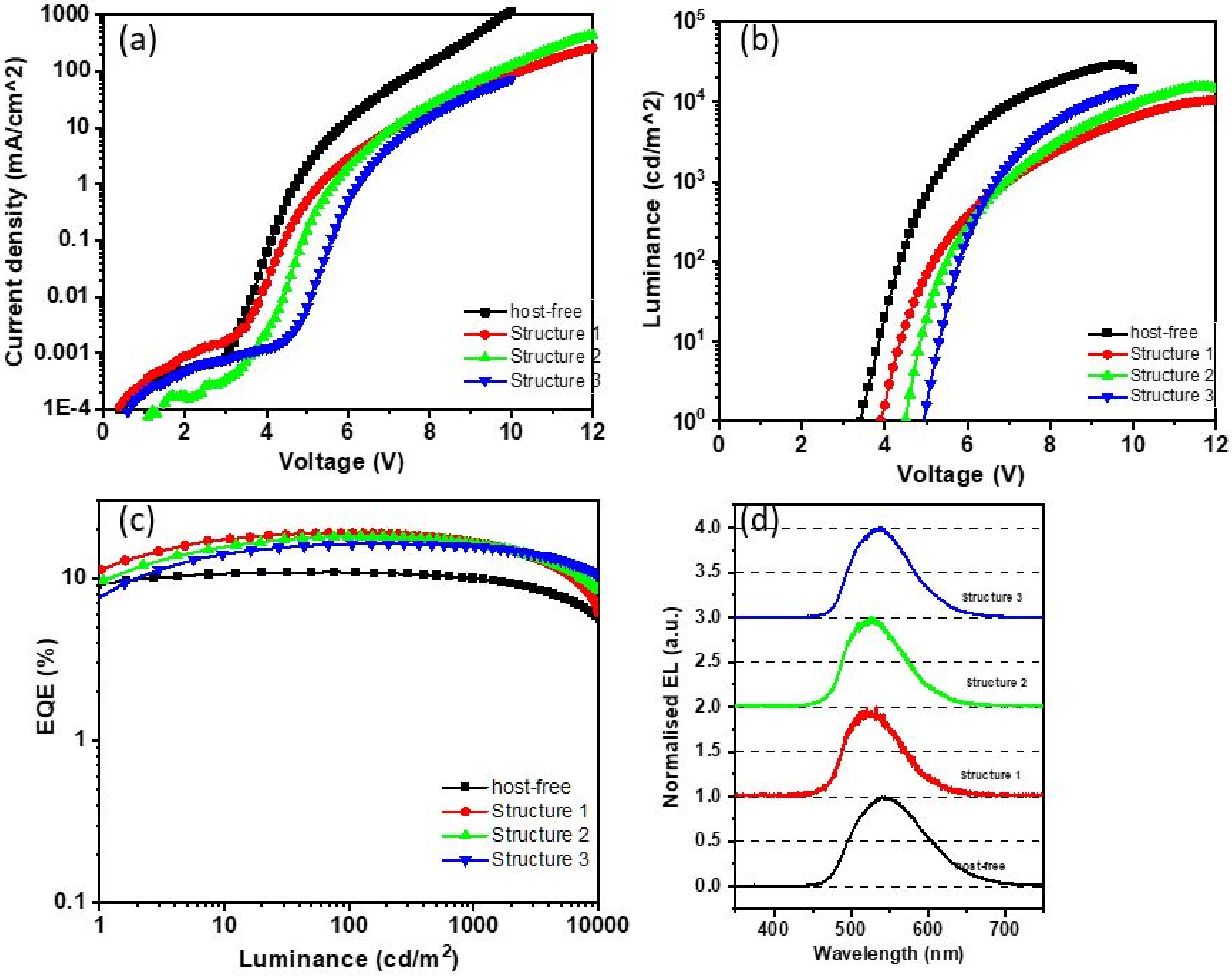

Figure S23. Typical OLED performance of Complex **4** in host free and different structures; **(a)** current-density vs. voltage; **(b)** luminance vs. voltage; **(c)** external quantum efficiency (EQE) vs. luminance; **(d)** electroluminescence (EL) spectra.

Table S6. OLED performance summary of Complex **4**

	Normal architecture				
	Turn-on (V)	EQE (max. %)	EQE % (100 cd/m²)	EQE % (1000 cd/m²)	EL peaks (nm)
**Host-free**	3.5	11.0	10.8	9.9	535
**Structure 1**	3.9	19.1	19.0	16.7	525
**Structure 2**	4.5	18.2	18.2	16.4	525
**Structure 3**	5.0	16.3	16.3	15.6	535

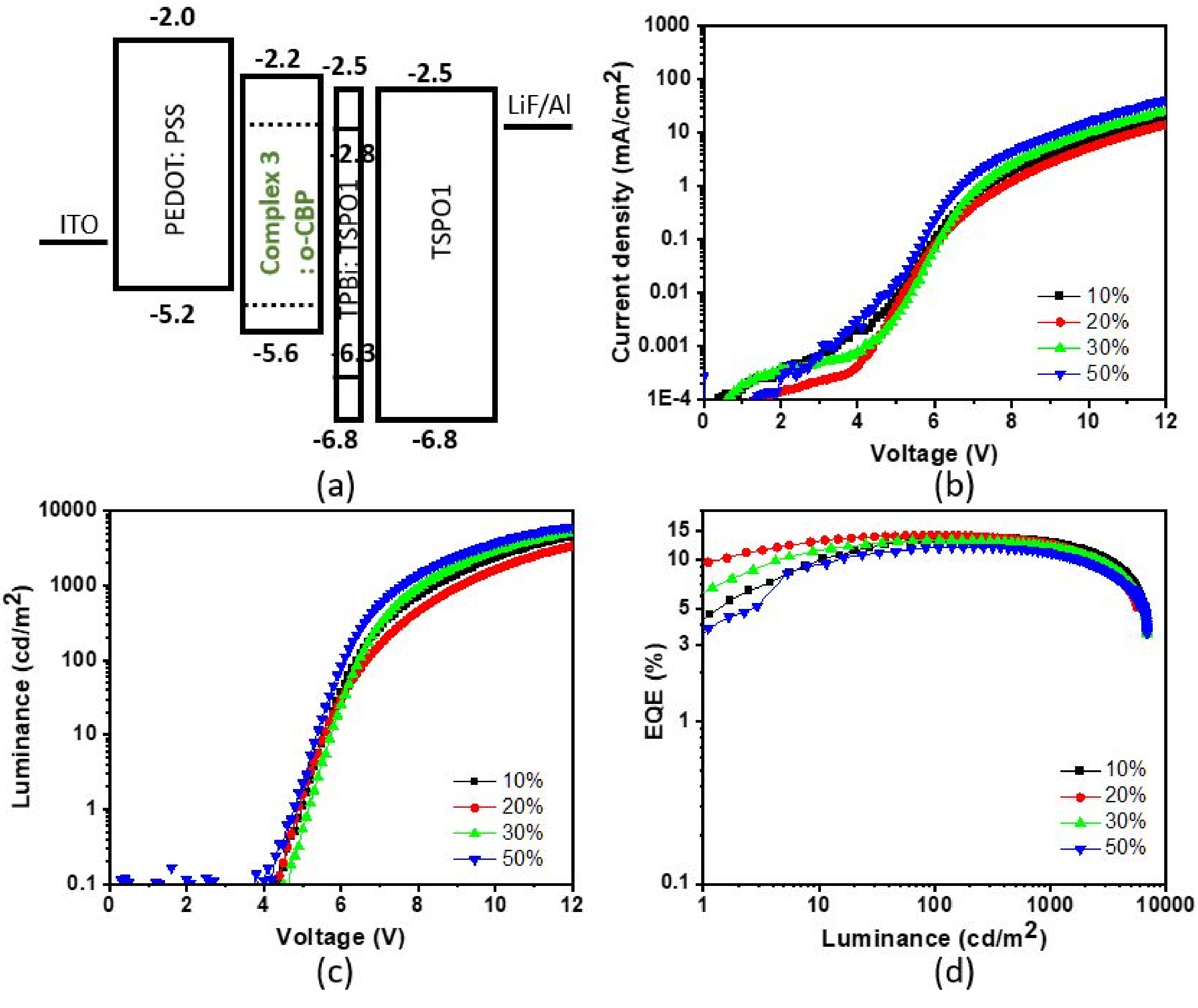

Figure S24 (a) Champion device structure for complex **3**; **(b)** Current-density vs. Voltage; **(c)** Luminance vs. Voltage; **(d)** external quantum efficiency (EQE) vs. Luminance for OLED device with varied doping concentration from 10 wt. % to 50 wt. % for complex 3. OLED device with 20 wt.% doping concentration for complex 3 shows the best performance.

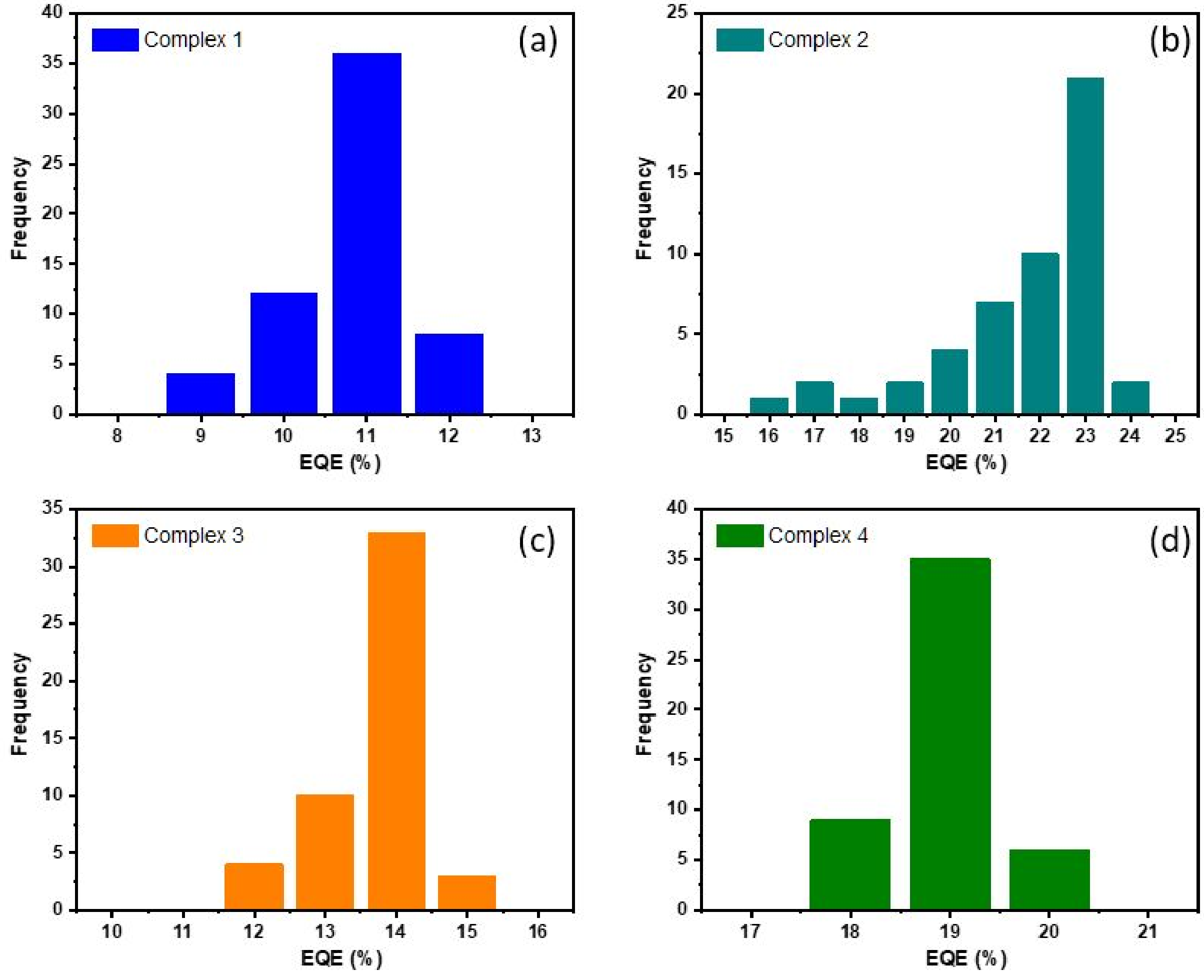

Figure S25. Maximum EQE histograms of the OLED devices based on complexes **1** (a), **2** (b), **3** (c) and **4** (d).

Computational Details

Compound 1 (Au):

	GS	S_1	T_1
φ	149.92	-116.09	-139.74
τ	-76.11	-88.06	-80.59
r_1	2.01	2.00	2.01
r_2	2.07	2.11	2.08

GS Electronic Structure:

State	Character	Osc. Str.	Energy / eV
GS	-		
T_1	^{3}LE(D)	-	3.14
T_2	^{3}CT	-	3.27
S_1	^{1}CT	0.141	3.40
T_3	^{3}LE(A)	-	3.47
T_4	^{3}LE(D)	-	3.60
T_5	^{3}LE(D)	-	3.88
S_2	^{1}LE(A)/^{1}LE(D)	-	3.92

SOC	T_1	T_2	T_3	T_4	T_5
S_1	0.844	0.246	0.510	7.771	0.327
S_2	0.845	7.543	1.056	0.597	0.052
S_3	0.612	3.010	1.820	0.600	0.049
S_4	0.146	0.550	0.715	1.582	0.182
S_5	0.477	0.974	0.525	0.176	0.737
T_1	-	1.148	0.492	1.653	0.125
T_2	-	-	0.613	12.713	0.655
T_3	-	-	-	0.769	0.031
T_4	-	-	-	-	0.477

S_1 Electronic Structure:

State	Character	Osc. Str.	Energy /
GS	-	-	0.39
T_1	^{3}CT	-	2.99
S_1	^{1}CT	0.044	3.05
T_2	^{3}LE(D)	-	3.43
T_3	^{3}LE(A)	0.006	3.44
S_2	^{1}LE(A)	-	3.83
T_4	^{3}LE(D)	-	3.88

SOC	T_1	T_2	T_3	T_4	T_5
S_1	0.223	1.071	7.976	0.626	2.198
S_2	8.877	0.039	2.073	0.192	4.434
S_3	0.712	0.071	1.161	0.622	0.535
S_4	0.997	0.050	0.455	1.949	0.296
S_5	0.487	0.006	0.633	0.038	0.865
T_1	-	1.236	12.179	0.812	3.292
T_2	-	-	0.066	0.099	0.173
T_3	-	-	-	0.209	6.624
T_4	-	-	-	-	0.557

T_1 Electronic Structure:

State	Character	Osc. Str.	Energy /
GS	-	-	0.21
T_1	^{3}LE(D)	-	3.01
T_2	^{3}CT	0.027	3.41
T_3	^{3}CT/^{3}LE(D)	-	3.43
S_1	^{1}CT	0.120	3.52
T_4	^{3}LE(A)	-	3.79
S_2	^{3}LE(D)	0.082	3.85

SOC	T_1	T_2	T_3	T_4	T_5
S_1	1.244	0.218	0.461	8.190	0.753
S_2	0.362	1.643	1.426	0.774	0.667
S_3	0.326	7.898	3.210	0.647	0.324
S_4	0.207	0.861	0.978	1.800	0.582
S_5	0.562	0.979	0.496	0.165	0.568
T_1	-	1.525	1.149	0.639	1.998
T_2	-	-	0.436	12.078	1.100
T_3	-	-	-	4.806	0.329
T_4	-	-	-	-	0.415

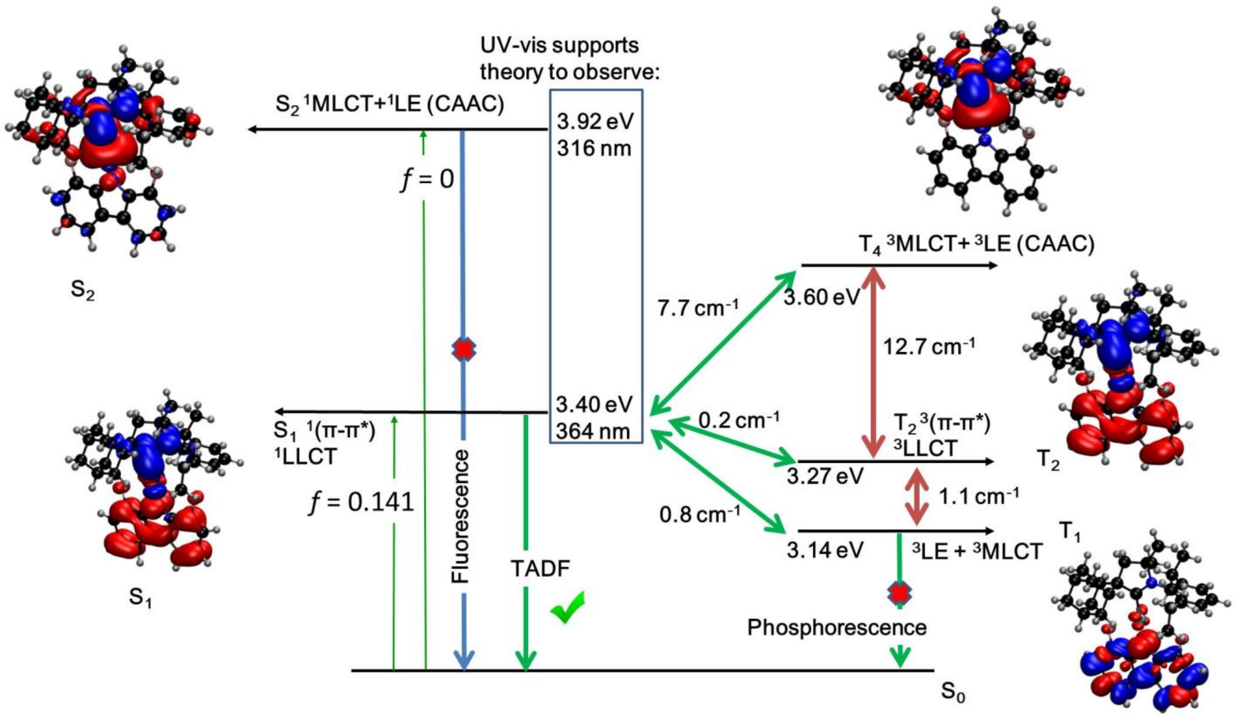

Figure S26. The difference of electronic density associated with the singlet and triplet excited states in the ground state geometry for complex **1**.

Compound 2 (Cu)

	GS	S_1	T_1
ϕ	86.31	83.21	81.93
τ	-83.84	-86.38	-84.86
r_1	1.92	1.90	1.88
r_2	1.93	1.91	1.94

GS Electronic Structure:

State	Character	Osc. Str.	Energy / eV
GS			
T_1	^{3}LE(A)	-	2.92
T_2	^{3}CT	-	3.06
S_1	^{1}CT	0.007	3.32
T_3	^{3}LE(A)	-	3.35
S_2	^{1}LE(A)/^{1}CT	0.005	3.39
T_4		-	3.48
S_3	^{1}LE(A)	0.086	3.85

SOC	T_1	T_2	T_3	T_4	T_5
S_1	82.398	12.106	403.738	36.150	14.806
S_2	319.478	2.776	91.124	8.592	6.532
S_3	22.586	1.265	6.858	1.426	0.527
S_4	15.255	2.824	75.698	7.417	8.216
S_5	15.347	1.234	20.633	1.877	46.119
T_1	-	14.399	500.070	44.804	26.857
T_2	-	-	3.175	1.512	0.456
T_3	-	-	-	1.683	10.558
T_4	-	-	-	-	1.065

S_1 Electronic Structure:

State	Character	Osc. Str.	Energy /
GS	-	-	0.32
T_1	^{3}CT/^{3}LE(A)	-	2.71
S_1	^{1}CT/^{1}LE(A)	0.003	3.01
T_2	^{3}CT	-	3.23
T_3	^{3}LE(D)	0.006	3.34
S_2	^{1}LE(D)	-	3.37
T_4	^{3}CT/^{3}LE(D)	-	3.82

SOC	T_1	T_2	T_3	T_4	T_5
S_1	97.773	338.421	15.128	2.385	441.673
S_2	323.345	182.515	9.907	2.662	486.511
S_3	24.668	14.905	9.487	0.459	2.653
S_4	38.985	51.799	2.563	1.683	105.984
S_5	570.508	138.277	4.258	1.338	399.822
T_1	-	483.552	22.718	4.871	747.891
T_2	-	-	2.364	0.439	455.930
T_3	-	-	-	1.528	19.281
T_4	-	-	-	-	3.452

T_1 Electronic Structure:

State	Character	Osc. Str.	Energy /
GS	-	-	0.30
T_1	^{3}LE(A)	-	2.68
S_1	^{1}CT/^{1}LE(A)	0.005	3.07
T_2	^{3}CT	-	3.23
S_2	^{1}CT	0.005	3.31
T_3	^{3}LE(D)	-	3.35
T_4	^{3}LE(D)	-	3.78

SOC	T_1	T_2	T_3	T_4	T_5
S_1	91.999	357.555	20.407	6.295	523.321
S_2	293.823	147.228	9.413	3.791	364.745
S_3	26.354	9.676	7.281	0.756	4.253
S_4	17.356	50.747	2.987	2.016	92.858
S_5	90.977	234.685	13.249	5.143	578.121
T_1	-	470.699	27.426	9.030	771.720
T_2	-	-	2.279	1.536	367.177
T_3	-	-	-	1.156	20.629
T_4	-	-	-	-	6.997

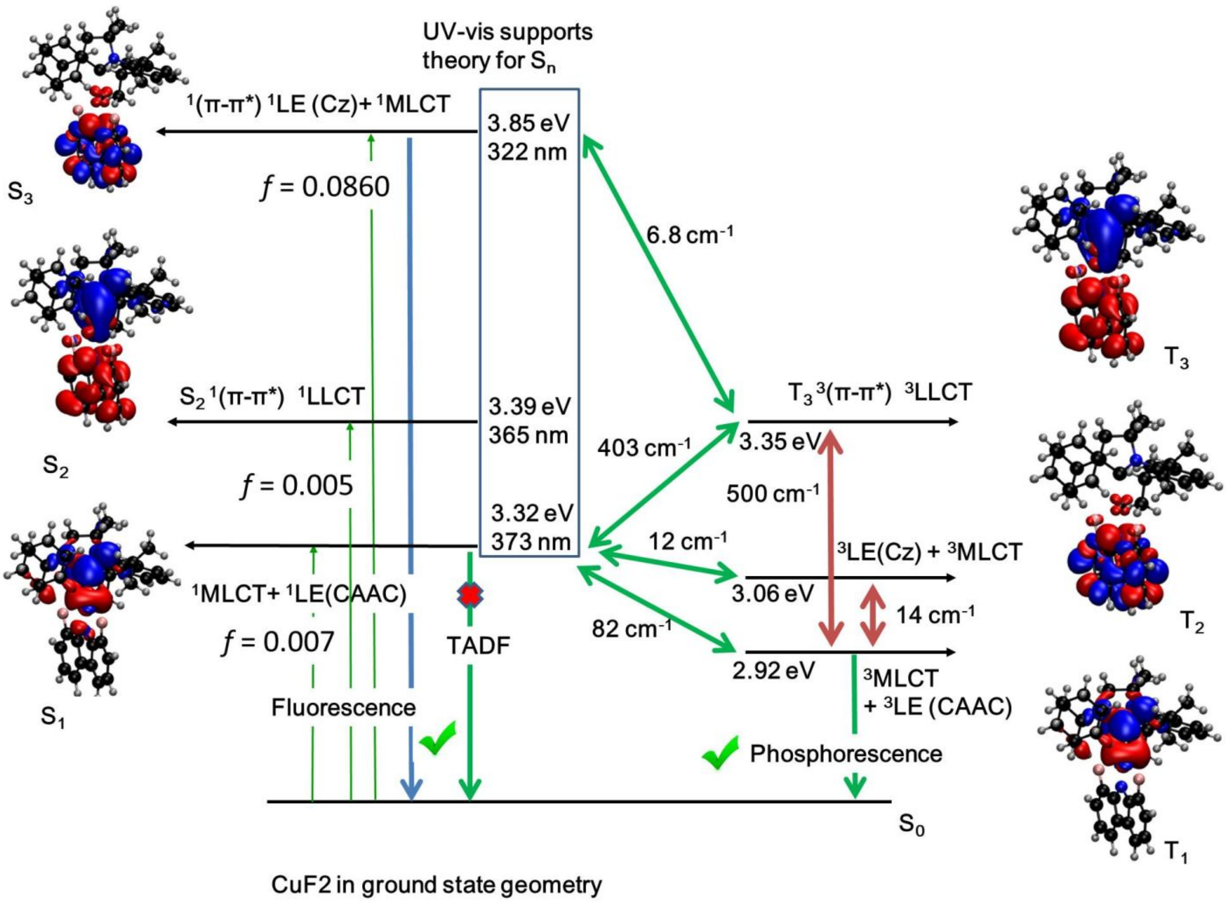

Figure S27. The difference of electronic density associated with the singlet and triplet excited states in the ground state geometry for complex **2**.

Compound 3 PLANAR:

	GS	S_1	T_1
ϕ	42.95	74.87	50.07
τ	-122.78	-108.36	-116.09
r_1	2.00	2.02	2.01
r_2	2.06	2.17	2.16

GS Electronic Structure:

State	Character	Osc. Str.	Energy /
GS	-	-	0.03
T_1	^{3}CT	-	2.95
S_1	^{1}CT	0.050	3.04
T_2	^{3}LE(D)	-	3.10
T_3	^{3}LE(D)	-	3.41
T_4	^{3}LE(A)	-	3.58
T_5	^{3}CT	0.003	3.70

SOC	T_1	T_2	T_3	T_4	T_5
S_1	0.587	1.567	0.453	5.977	0.628
S_2	0.920	0.064	1.659	1.568	0.264
S_3	1.825	0.444	1.798	0.690	0.457
S_4	6.185	0.348	0.775	0.892	2.499
S_5	1.122	0.639	0.221	0.212	0.043
T_1	-	2.347	0.791	9.844	0.911
T_2	-	-	0.924	0.620	0.240
T_3	-	-	-	1.132	2.040
T_4	-	-	-	-	2.901

S_1 Electronic Structure:

State	Character	Osc. Str.	Energy /
GS	-	-	0.64
T_1	^{3}CT	-	2.69
S_1	^{1}CT	0.003	2.73
T_2	^{3}LE(A)	-	3.50
T_3	^{3}LE(D)	-	3.51
T_4	^{3}CT	-	3.69
S_2	^{1}CT	0.003	3.69
S_3	^{1}LE(A)	0.009	3.95
T_5	^{3}LE(D)	-	3.99

SOC	T_1	T_2	T_3	T_4	T_5
S_1	0.364	4.786	1.344	0.941	0.773
S_2	1.118	0.372	0.051	0.107	0.960
S_3	5.162	2.353	0.265	1.136	0.449
S_4	1.320	0.387	0.263	0.332	2.249
S_5	0.927	0.811	0.179	0.194	0.453
T_1	-	7.382	1.822	1.403	1.207
T_2	-	-	0.321	0.992	0.691
T_3	-	-	-	0.145	0.944
T_4	-	-	-	-	1.302

T_1 Electronic Structure:

State	Character	Osc. Str.	Energy /
GS	-	-	0.48
T_1	^{3}CT	-	2.65
S_1	^{1}CT	0.003	2.73
T_2	^{3}LE(D)	-	3.37
T_3	^{3}LE(A)	-	3.55
T_4	^{3}CT	-	3.64
S_2	^{1}CT	0.003	3.65
T_5	^{3}CT	-	3.83

SOC	T_1	T_2	T_3	T_4	T_5
S_1	0.380	1.341	4.776	0.891	0.934
S_2	1.062	0.024	0.782	0.197	0.863
S_3	4.980	0.211	1.568	1.579	0.416
S_4	1.570	0.479	0.361	0.332	2.078
S_5	1.233	0.055	0.950	0.193	0.568
T_1	-	1.989	7.880	1.425	1.407
T_2	-	-	0.356	0.131	0.985
T_3	-	-	-	1.562	0.721
T_4	-	-	-	-	1.101

Compound 3 Orthogonal:

	GS	S_1	T_1
ϕ	88.46	89.71	90.42
τ	-58.51	-70.28	-68.96
r_1	2.01	2.01	2.01
r_2	2.07	2.15	2.15

GS Electronic Structure:

State	Character	Osc. Str.	Energy / eV
GS	-		
T_1	^{3}CT	-	2.91
S_1	^{1}CT	0.003	2.95
T_2	^{3}LE(D)	-	3.06
T_3	^{3}LE(D)	-	3.38
T_4	^{3}LE(A)	-	3.57
T_5	^{3}CT	-	3.66
S_2	^{1}CT	0.002	3.67

SOC	T_1	T_2	T_3	T_4	T_5
S_1	0.732	0.851	0.068	7.473	0.860
S_2	0.876	0.159	1.123	1.003	0.186
S_3	0.977	0.547	1.971	0.944	0.283
S_4	7.387	0.308	1.076	0.595	2.150
S_5	2.625	0.987	0.384	0.139	0.079
T_1	-	1.263	1.032	10.893	1.009
T_2	-	-	1.008	0.511	0.132
T_3	-	-	-	1.698	1.708
T_4	-	-	-	-	2.683

S_1 Electronic Structure:

State	Character	Osc. Str.	Energy /
GS	-	-	0.41
T_1	^{3}CT	-	2.65
S_1	^{1}CT	0.001	2.67
T_2	^{3}LE(D)	-	3.29
T_3	^{3}LE(A)	-	3.59
T_4	^{3}CT	-	3.62
S_2	^{1}CT	0.002	3.63
T_5	-	-	3.76

SOC	T_1	T_2	T_3	T_4	T_5
S_1	0.380	0.328	6.244	1.005	0.632
S_2	1.047	0.050	0.795	0.090	0.372
S_3	5.918	0.058	0.451	1.642	0.676
S_4	0.485	0.723	0.518	0.060	1.810
S_5	1.675	0.856	0.161	0.025	0.439
T_1	-	0.526	9.177	1.371	0.568
T_2	-	-	0.143	0.056	0.365
T_3	-	-	-	1.920	0.996
T_4	-	-	-	-	0.484

T_1 Electronic Structure:

State	Character	Osc. Str.	Energy / eV
GS	-	-	0.40
T_1	^{3}CT	-	2.65
S_1	^{1}CT	0.003	2.67
T_2	^{3}LE(D)	-	3.28
T_3	^{3}LE(A)	-	3.59
T_4	^{3}CT	-	3.62
S_2	^{1}CT	0.003	3.63

SOC	T_1	T_2	T_3	T_4	T_5
S_1	0.402	0.285	6.242	0.981	0.686
S_2	1.034	0.049	0.809	0.048	0.362
S_3	5.923	0.066	0.506	1.675	0.698
S_4	0.431	0.633	0.522	0.053	1.852
S_5	1.626	0.907	0.154	0.017	0.441
T_1	-	0.466	9.196	1.312	0.550
T_2	-	-	0.153	0.044	0.461
T_3	-	-	-	1.990	1.049
T_4	-	-	-	-	0.478

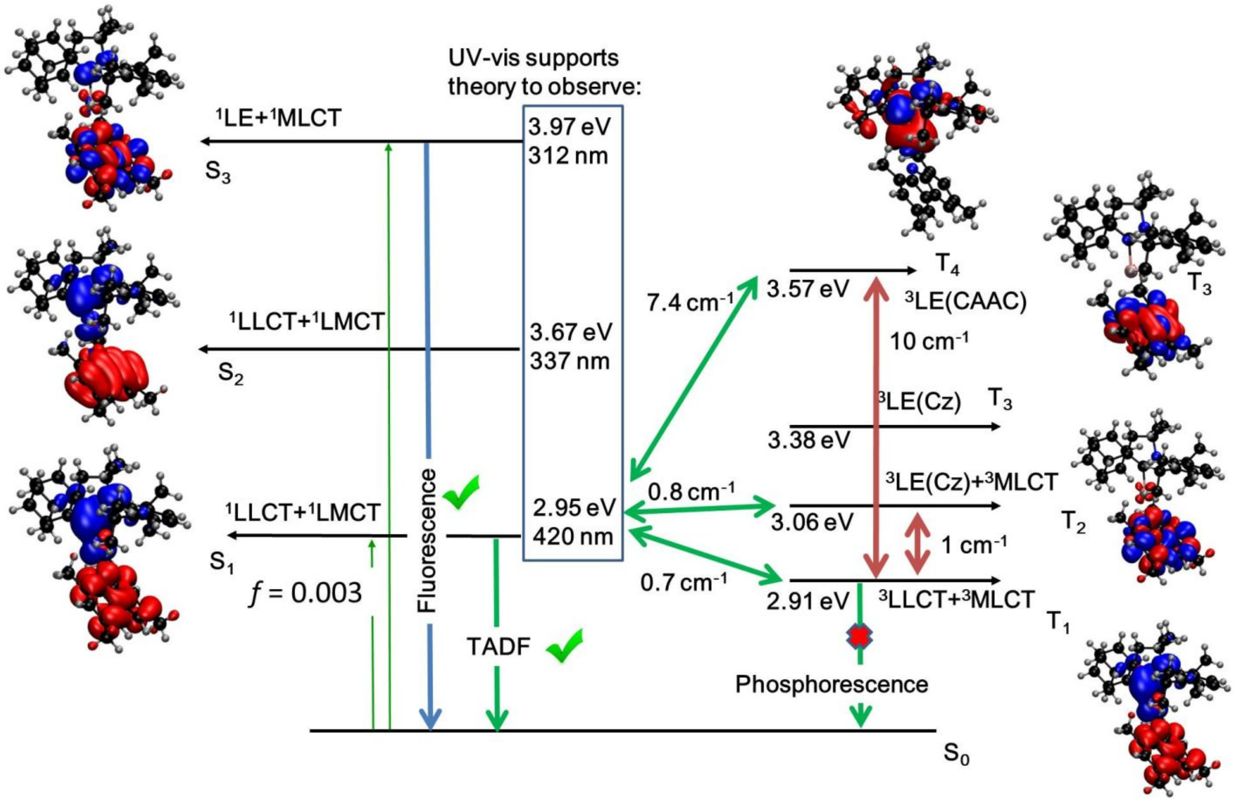

Figure S28. The difference of electronic density associated with the singlet and triplet excited states in the ground state geometry for complex **3**.

Compound 4:

	GS	S_1	T_1
ϕ	172.19	93.78	125.14
τ	-126.70	-91.82	-106.75
r_1	2.01	2.01	2.01
r_2	2.06	2.13	2.13

GS Electronic Structure:

State	Character	Osc. Str.	Energy / eV
GS	-	-	0.00
T_1	$^3CT/^3LE(D)$	-	2.99
T_2	$^3CT/^3LE(D)$	-	3.10
T_3	$^3CT/^3LE(D)$	-	3.16
S_1	^1CT	0.131	3.21
T_4	$^3LE(D)$	-	3.35
S_2	^1CT	-	3.40
T_5	^3CT	-	3.50

SOC	T_1	T_2	T_3	T_4	T_5
S_1	0.460	0.516	0.882	0.578	1.574
S_2	0.581	0.719	0.167	0.300	0.298
S_3	1.093	1.627	0.422	1.424	1.106
S_4	1.073	0.736	1.076	0.491	0.123
S_5	3.739	1.714	4.447	1.769	0.826
T_1	-	0.938	1.358	0.853	0.987
T_2	-	-	0.481	0.770	0.922
T_3	-	-	-	1.287	1.598
T_4	-	-	-	-	1.155

S_1 Electronic Structure:

State	Character	Osc. Str.	Energy / eV
GS	-	-	0.42
T_1	^3CT	-	2.72
S_1	^1CT	0.000	2.73
T_2	^3CT	-	3.19
S_2	^1CT	0.000	3.19
T_4	$^3LE(D)$	-	3.35
T_5	$^3LE(A)$	-	3.37

SOC	T_1	T_2	T_3	T_4	T_5
S_1	0.041	0.826	2.783	5.224	0.351
S_2	0.790	0.030	0.076	2.470	2.425
S_3	5.645	2.376	0.252	1.935	0.155
S_4	2.635	0.728	0.704	0.394	1.663
S_5	0.977	0.849	0.390	3.092	0.454
T_1	-	1.121	3.986	7.676	0.533
T_2	-	-	0.120	3.635	3.430
T_3	-	-	-	0.123	0.572
T_4	-	-	-	-	0.059

T_1 Electronic Structure:

State	Character	Osc. Str.	Energy / eV
GS	-	-	0.39
T_1	^3CT	-	2.71
S_1	^1CT	0.027	2.77
T_2	^3CT	-	3.19
S_2	^1CT	0.000	3.21
T_3	$^3LE(D)$	-	3.31
T_4	$^3LE(A)$	-	3.39

SOC	T_1	T_2	T_3	T_4	T_5
S_1	0.426	1.142	2.016	5.169	0.484
S_2	1.108	0.331	0.129	2.237	2.087
S_3	5.444	2.298	1.069	1.659	0.643
S_4	1.990	0.471	0.925	0.510	1.988
S_5	0.947	0.917	0.527	3.070	0.260
T_1	-	1.461	3.618	7.833	0.713
T_2	-	-	0.366	3.320	2.958
T_3	-	-	-	1.487	1.493
T_4	-	-	-	-	0.781

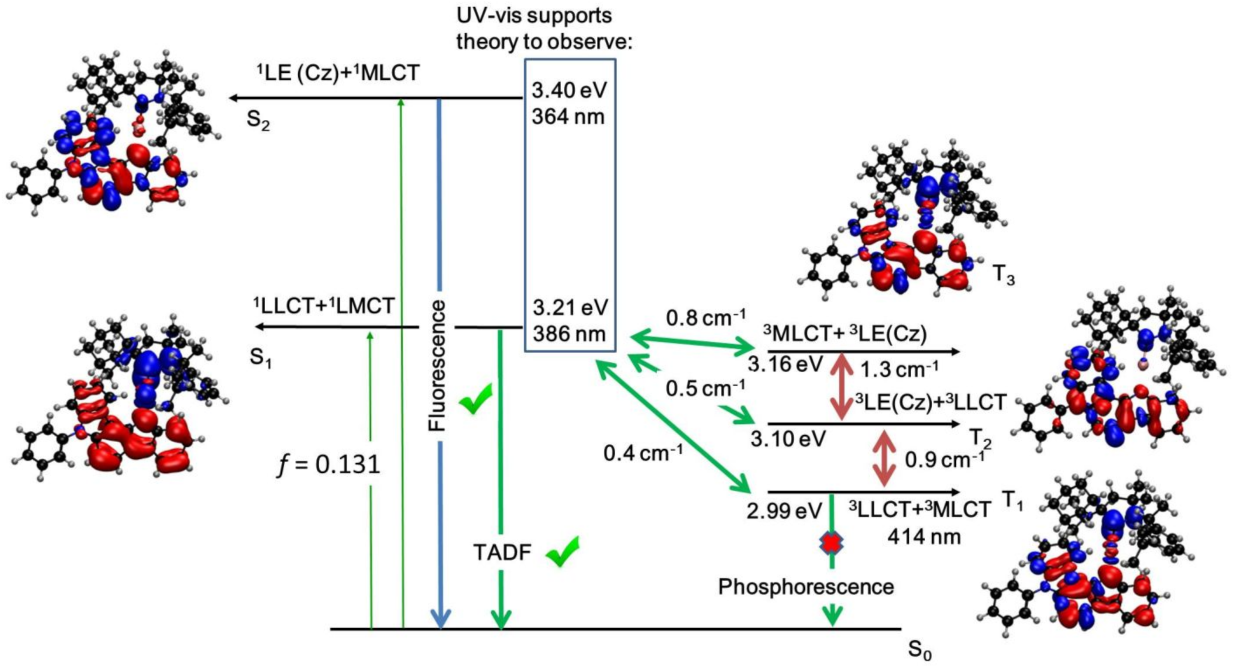

Figure S29. The difference of electronic density associated with the singlet and triplet excited states in the ground state geometry for complex **4**.

Table S7. Energy of the HOMO and LUMO (in eV) and their overlap (in %). Cartesian components of dipole moments for the ground (μ^{GS}) and first excited state (μ^{ES}) with their absolute value (in Debye). Values are provided for the optimised geometry of the ground state (GS), first singlet (S_1) and triplet (T_1) excited states.

	Complex **1**			Complex **2**			Complex **3**		
	GS	S_1	T_1	GS	S_1	T_1	GS	S_1	T_1
HOMO	-5.50	-5.36	-5.39	-5.36	-5.31	-5.36	-5.14	-4.27	-4.93
LUMO	-1.42	-2.01	-1.39	-1.17	-1.66	-1.61	-1.42	-2.42	-2.01
**Overlap**	**27.58**	**19.40**	**26.21**	**13.55**	**15.82**	**14.28**	**23.50**	**16.01**	**20.27**
μ_x^{GS}	-1.21	-0.50	-0.78	1.35	0.56	0.85	14.66	15.88	15.57
μ_y^{GS}	0.11	-0.26	-0.09	0.05	-0.58	-0.49	-2.52	-2.39	-2.42
μ_z^{GS}	11.78	12.32	11.98	11.49	11.22	11.19	0.83	0.85	0.81
$\|\|\mu^{GS}\|\|$	**11.84**	**12.33**	**12.01**	**11.57**	**11.25**	**11.24**	**14.90**	**16.08**	**15.77**
μ_x^{ES}	-1.11	0.82	-1.02	0.32	-1.29	-0.66	-7.02	-8.40	-7.93
μ_y^{ES}	-0.32	-0.72	-0.49	-0.39	-0.86	-0.82	1.38	0.50	1.38
μ_z^{ES}	14.04	-11.31	14.85	5.77	3.61	5.31	-0.05	-1.18	-0.27
$\|\|\mu^{ES}\|\|$	**14.09**	**11.37**	**14.89**	**5.79**	**3.93**	**5.41**	**7.16**	**8.50**	**8.06**

Table S8. Coordinates for ground and excited states (S1 and T1) geometries from TD-DFT calculations for complexes **1–4** and CMA1.

Complex CMA1, Ground state S0.

C	-2.1108263740	-2.1790979610	-4.8949238118
C	-1.2357550093	-1.1577001007	-5.2501914675
C	-0.5378942466	-0.4767366696	-4.2434549388
C	-0.7245353676	-0.8313797228	-2.8745792368
C	-1.6100301425	-1.8647934925	-2.5250323138
C	-2.2920748408	-2.5245052975	-3.5411902919
N	0.0481046686	-0.0442229528	-2.0504531442
C	0.7349376741	0.8279496905	-2.8648827682
C	0.4141509591	0.6057808070	-4.2373887132
C	1.0061791700	1.3899556227	-5.2368963768
C	1.9074651158	2.3848506756	-4.8725219769
C	2.2184696135	2.6026024335	-3.5160253875
C	1.6432639786	1.8378774425	-2.5074839095
Au	0.0881351676	-0.1478161779	-0.0056032747
C	0.0631170614	-0.2784492290	1.9996800712
N	-0.7518707635	-1.1138555121	2.6002180012
C	-0.6941253285	-1.1067623627	4.1092136685
C	0.6080044470	-0.3355933379	4.3156275480
C	0.8611938114	0.4986251482	3.0410407076
C	2.3857306477	0.5927487851	2.7478606390
C	3.0703460176	1.2846242975	3.9379082676
C	2.5426521507	2.7169466880	4.0855852007
C	1.0277521759	2.6856331293	4.3098518337
C	0.3387337340	1.9745698491	3.1360820343
C	2.7018183403	1.3982163831	1.4860509736
C	2.1625566196	2.8236066124	1.6216514372
C	0.6510839803	2.7694940356	1.8600655776
C	2.8452766774	3.5076619204	2.8097679226
C	-1.6482945831	-1.9875553906	1.8731236515
C	-2.9438433284	-1.5383517376	1.5317869743
C	-3.7906879613	-2.4313953738	0.8640909899
C	-3.3748462117	-3.7123389913	0.5241846143
C	-2.0774412110	-4.1128572286	0.8200538657
C	-1.1845008465	-3.2661594527	1.4861119180
C	-3.4483199890	-0.1232318800	1.7724771895
C	-3.5965780399	0.6337139619	0.4482653163
C	0.2467906712	-3.7454089681	1.6785355437
C	0.3234890725	-5.0896459255	2.4057416203
C	-1.9055118681	-0.3942723395	4.7074530222
C	-0.6267200698	-2.5138998523	4.6879940082
C	-4.7671578784	-0.0961551534	2.5484023588
C	0.9735447914	-3.8296630949	0.3319506179
H	-1.7851026072	-0.3706093820	5.8002601449
H	-2.8356039272	-0.9361837515	4.4876547534
H	-2.0030619397	0.6409640799	4.3558797581
H	2.9152446954	0.7172154230	4.8695600224

H	4.1591754994	1.3020109289	3.7617301471
H	1.4335931840	-1.0569542752	4.4270917418
H	0.5726235420	0.2576556971	5.2362851309
H	2.7794594020	-0.4328562622	2.6422863796
H	-0.7518984290	1.9690525165	3.2970005137
H	0.2567604447	-3.0607900850	4.3370058204
H	-1.5302569393	-3.0969436787	4.4542904517
H	-0.5561946495	-2.4327485030	5.7829285840
H	0.1396908970	2.3351948553	0.9852290844
H	0.2533170510	3.7913231557	1.9816312198
H	3.9347655926	3.5595816988	2.6427435482
H	2.4824304527	4.5447174818	2.9130203001
H	-4.7971070671	-2.1028361593	0.5927733936
H	3.7954956112	1.4163420030	1.3403806076
H	2.2743361694	0.9087918141	0.5951916612
H	0.7809350855	-3.0003294500	2.2860111103
H	-2.6440081199	0.6710762666	-0.1030090869
H	-3.9240276736	1.6682974832	0.6377787150
H	-4.3469957884	0.1573799336	-0.2026147660
H	-1.7380468622	-5.1054172753	0.5134381300
H	0.6363955450	3.7151737854	4.3755898988
H	0.7892302110	2.2059312251	5.2717023941
H	3.0310674613	3.2006944988	4.9483424802
H	2.3678846513	3.3884357953	0.6964432657
H	-2.6976863029	0.4140089619	2.3669220660
H	-4.0552462373	-4.3919533273	0.0048938914
H	-4.7040623654	-0.6579338581	3.4920455232
H	-5.5899206961	-0.5277878113	1.9571636501
H	-5.0450491995	0.9424742541	2.7868856182
H	-0.2209791248	-5.0736510158	3.3609647610
H	1.3740389611	-5.3465981624	2.6131448409
H	-0.0969763368	-5.9022682641	1.7928899300
H	0.9618985440	-2.8624262918	-0.1944143457
H	0.5041423809	-4.5784339770	-0.3258968532
H	2.0235707631	-4.1259868465	0.4838988149
H	1.8884117659	2.0184784039	-1.4577841522
H	2.9266545533	3.3923338214	-3.2491540566
H	-1.7599256446	-2.1411916616	-1.4773819017
H	-2.9850925704	-3.3303419216	-3.2822090095
H	0.7610077976	1.2217148884	-6.2896856846
H	2.3771343154	3.0048701407	-5.6405616967
H	-1.0949324262	-0.8879462115	-6.3009981653
H	-2.6635070630	-2.7184270569	-5.6686243803

Complex CMA1, Excited state S1.

C	-1.7927260227	2.6075151888	-4.7387665132
C	-0.9271472128	1.6049078630	-5.2141290935
C	-0.3289331185	0.7609076231	-4.2952528022
C	-0.5971452248	0.9099305347	-2.9037564718
C	-1.4530279307	1.9160638881	-2.4340243902

C	-2.0465699937	2.7609222224	-3.3718276497
N	0.0967880976	-0.0154536440	-2.1615599421
C	0.8416594392	-0.7603210547	-3.0438419761
C	0.6190323939	-0.3417649595	-4.3880060219
C	1.2776120958	-0.9684622026	-5.4311388568
C	2.1697328771	-2.0136340824	-5.1280964218
C	2.3967160245	-2.4163089298	-3.8078989189
C	1.7369405681	-1.7971133352	-2.7463986186
Au	0.0383754449	-0.2122873792	-0.0885965963
C	0.0273030880	-0.3267988108	1.9083111777
N	-0.8442085803	-1.1642183223	2.6141282326
C	-0.7042235506	-1.0800893114	4.0845236202
C	0.6455999376	-0.3654184722	4.1984912622
C	0.8553638710	0.4710707769	2.9172972684
C	2.3763766294	0.5598573492	2.5992265230
C	3.1214497302	1.2345149109	3.7632625706
C	2.6202992766	2.6720406928	3.9457503358
C	1.1148625648	2.6573534033	4.2268211244
C	0.3761134110	1.9573325737	3.0762384153
C	2.6634287107	1.3711104061	1.3330337998
C	2.1467424632	2.8020055740	1.4992797056
C	0.6450979881	2.7582598877	1.7960447789
C	2.8823513886	3.4691966022	2.6649930172
C	-1.7689200091	-2.0333380871	1.9676125015
C	-3.0769577417	-1.5887933063	1.6480341099
C	-3.9786052499	-2.4888714881	1.0652414027
C	-3.6111365255	-3.7969237387	0.7734770286
C	-2.3129888227	-4.2142357251	1.0417519213
C	-1.3747908854	-3.3530074022	1.6239929631
C	-3.5373574596	-0.1558615008	1.8571078940
C	-3.7888309471	0.5356931969	0.5149234123
C	0.0452429830	-3.8654101172	1.8031946423
C	0.1131126052	-5.1586145299	2.6191815661
C	-1.8343505872	-0.2802723230	4.7452378721
C	-0.6554364139	-2.4454698011	4.7683562579
C	-4.7861501647	-0.0568518423	2.7361291231
C	0.7202272026	-4.0703900276	0.4443911676
H	-1.6334484844	-0.1789911324	5.8231765928
H	-2.7961704705	-0.8016114916	4.6363206655
H	-1.9366511164	0.7283489286	4.3241978247
H	2.9966443467	0.6627831659	4.6972955490
H	4.2043418825	1.2423264518	3.5467344624
H	1.4424793190	-1.1271403662	4.2439490439
H	0.7046557682	0.2131935377	5.1289872467
H	2.7538532080	-0.4698093048	2.4683006902
H	-0.7082846599	1.9675691005	3.2748494359
H	0.1792443160	-3.0549208267	4.4005290263
H	-1.5917602242	-3.0083860141	4.6288688275
H	-0.5153334469	-2.3015042675	5.8506834479
H	0.1024622629	2.3084004181	0.9470460429
H	0.2554380641	3.7839576424	1.9203440516

H	3.9658206521	3.5109107059	2.4567962690
H	2.5366219444	4.5102966805	2.7900490920
H	-4.9910867974	-2.1494085234	0.8289664888
H	3.7514834540	1.3798595316	1.1433921830
H	2.1924896672	0.8941075895	0.4555566798
H	0.6093280227	-3.0854536679	2.3349445469
H	-2.8930538933	0.4886692244	-0.1216688479
H	-4.0454447483	1.5963437052	0.6693490582
H	-4.6234376587	0.0649121941	-0.0297688309
H	-2.0127686718	-5.2342195096	0.7863182295
H	0.7434323492	3.6931324185	4.3228925811
H	0.9107496545	2.1705024474	5.1936661614
H	3.1503793348	3.1437417568	4.7914325138
H	2.3247744388	3.3727885256	0.5712082403
H	-2.7169210221	0.3812965341	2.3515383615
H	-4.3314009944	-4.4859555466	0.3240137970
H	-4.6589423535	-0.5723840518	3.6993269327
H	-5.6629308133	-0.4975699426	2.2349948080
H	-5.0251185763	0.9984751493	2.9445171728
H	-0.3953944257	-5.0634935090	3.5894377689
H	1.1627671019	-5.4349006817	2.8091191835
H	-0.3514903881	-5.9981422829	2.0779869497
H	0.6833466657	-3.1470426365	-0.1523958220
H	0.2256453135	-4.8701430560	-0.1312192083
H	1.7767257208	-4.3554007941	0.5756677891
H	1.9104795362	-2.0987379997	-1.7121601489
H	3.1007332956	-3.2260650859	-3.6049370364
H	-1.6338776109	2.0324113952	-1.3640554110
H	-2.7158502959	3.5547833745	-3.0334929130
H	1.1168776613	-0.6619353910	-6.4672215904
H	2.6978435287	-2.5169564784	-5.9411097960
H	-0.7328131831	1.5051331473	-6.2844299481
H	-2.2708200241	3.2822466356	-5.4524375825

Complex CMA1, Excited state T1.

C	-1.9600266251	-2.3525284454	-4.8810646826
C	-1.1770973717	-1.2500273808	-5.2749728258
C	-0.5197852042	-0.5265622169	-4.2957077467
C	-0.6418112799	-0.9031003908	-2.9269139800
C	-1.4224024673	-2.0012378433	-2.5359810978
C	-2.0789183805	-2.7190359463	-3.5363466646
N	0.0788513056	-0.0630510962	-2.1157493931
C	0.6730087876	0.8714355230	-2.9268650880
C	0.3492319883	0.6448111306	-4.2958038978
C	0.8517410055	1.4835836827	-5.2752759072
C	1.6801201475	2.5521228041	-4.8820888807
C	1.9937562749	2.7743707533	-3.5370623415
C	1.4948584784	1.9384161834	-2.5376104008
Au	0.1433439387	-0.1646124230	-0.0480439596
C	0.1067290143	-0.2640190613	1.9473737088

N	-0.7796849209	-1.1352302931	2.5878066995
C	-0.7125935025	-1.0927086150	4.0643308257
C	0.6218392925	-0.3645889946	4.2630530454
C	0.8849109649	0.5045500220	3.0119977629
C	2.4171192042	0.6008071557	2.7571389883
C	3.1153791764	1.2588359960	3.9582738438
C	2.6051013306	2.6926868048	4.1416625748
C	1.0911037770	2.6673591883	4.3693917000
C	0.3952359067	1.9852475053	3.1821997691
C	2.7427530755	1.4291287323	1.5125366216
C	2.2162473331	2.8561204282	1.6812202652
C	0.7053309224	2.8062711044	1.9244078354
C	2.9092211287	3.5081110889	2.8815034057
C	-1.6641053224	-1.9834260261	1.8602060206
C	-2.9510857728	-1.5267714127	1.4773331653
C	-3.8070501762	-2.4025485889	0.7970419085
C	-3.4108057243	-3.6929624303	0.4658491613
C	-2.1264895073	-4.1150264306	0.7898218778
C	-1.2329879328	-3.2779587440	1.4699281807
C	-3.4317824193	-0.1048645173	1.7190927250
C	-3.6018989502	0.6494604702	0.3964537268
C	0.1804898401	-3.7867284324	1.7068887156
C	0.2081083469	-5.1114948320	2.4733507848
C	-1.8848516369	-0.3245787852	4.6880453608
C	-0.6836024580	-2.4807461277	4.7015573667
C	-4.7335911091	-0.0528470650	2.5222388769
C	0.9380904192	-3.9311843658	0.3830896351
H	-1.7419699407	-0.2548094235	5.7775488723
H	-2.8335170352	-0.8516172200	4.5110007046
H	-1.9748204981	0.6955747102	4.2922261977
H	2.9515605880	0.6735827040	4.8778824629
H	4.2060067412	1.2693232340	3.7853877872
H	1.4249314770	-1.1179800936	4.3293908339
H	0.6297704203	0.1929939271	5.2086880371
H	2.8022437442	-0.4260119842	2.6259152056
H	-0.6954163822	1.9869825933	3.3436106135
H	0.1849836608	-3.0627812609	4.3683995057
H	-1.5981852923	-3.0508515054	4.4741669550
H	-0.6204863517	-2.3783291860	5.7958369276
H	0.1930208853	2.3681328474	1.0512395638
H	0.3100881360	3.8295519942	2.0507514471
H	3.9991396036	3.5553950054	2.7116535852
H	2.5559785888	4.5463857317	3.0090282032
H	-4.8049456539	-2.0566646596	0.5127137589
H	3.8358965960	1.4429145992	1.3551286916
H	2.2969009913	0.9587656495	0.6191992020
H	0.7065549254	-3.0268597806	2.3032965727
H	-2.6623606617	0.6583324219	-0.1780065685
H	-3.8967448102	1.6942900517	0.5862866922
H	-4.3837930954	0.1899982551	-0.2300259594
H	-1.8004429425	-5.1177875860	0.4995653345

H	0.7114951596	3.6996616853	4.4705761262
H	0.8564340217	2.1612113407	5.3191826441
H	3.1031741349	3.1545895120	5.0118268513
H	2.4251388979	3.4410489664	0.7680706158
H	-2.6493706120	0.4124392309	2.2903510324
H	-4.0960210619	-4.3631516226	-0.0604209396
H	-4.6603818659	-0.6138531977	3.4655415648
H	-5.5751729745	-0.4733361351	1.9486473360
H	-4.9924700811	0.9901081088	2.7657617297
H	-0.3554372650	-5.0534856316	3.4158345179
H	1.2461186151	-5.3935865280	2.7119167774
H	-0.2233815716	-5.9302102634	1.8754671973
H	0.9608487184	-2.9777959539	-0.1674014698
H	0.4681642065	-4.6885514421	-0.2656480953
H	1.9778859213	-4.2465750943	0.5675374428
H	1.7279713384	2.1047549342	-1.4841213594
H	2.6364764514	3.6144140997	-3.2651762149
H	-1.5116146773	-2.2779050225	-1.4826018121
H	-2.6957070254	-3.5782196485	-3.2637682106
H	0.6145147491	1.3279218122	-6.3301703313
H	2.0837950349	3.2222760528	-5.6444935068
H	-1.0971259472	-0.9780884154	-6.3299360108
H	-2.4863636672	-2.9315627587	-5.6432959820

Complex 1, Ground state S0.

C	0.0008842877	-0.0289037376	1.9897406761
N	-0.8206727273	-0.7652938102	2.7030423246
F	-2.6987241816	-1.2210475650	-1.3008173214
Au	-0.0117985220	-0.1213808109	-0.0187781354
N	-0.0329282923	-0.2408075728	-2.0897035122
F	2.8411368242	0.0359280924	-1.4940723301
C	0.8699056143	0.8106128189	2.9257920806
C	0.7033617163	0.0518588859	4.2574660058
H	1.5187538988	-0.6850591570	4.3407381023
H	0.7569556919	0.6919368474	5.1445253800
C	-0.6295398832	-0.6896776604	4.2013375772
C	-1.8554297088	-1.5960328813	2.1245429880
C	-1.5609355654	-2.9400950951	1.7950771737
C	-2.6062231108	-3.7381499283	1.3178935429
H	-2.4024943101	-4.7795751051	1.0572310134
C	-3.8904301741	-3.2330645242	1.1575530194
H	-4.6920560474	-3.8795111766	0.7909586090
C	-4.1437454799	-1.8964872638	1.4359117290
H	-5.1448681977	-1.4927961888	1.2662366183
C	-3.1399659894	-1.0446643969	1.9097528236
C	-3.4796106225	0.4267191077	2.0958739779
H	-2.6275950340	0.9158672256	2.5844533377
C	-3.6811580100	1.1246596928	0.7465109647
H	-3.8993176722	2.1927511767	0.9053396785
H	-4.5279613926	0.6845902225	0.1968762748

H	-2.7881863716	1.0457080101	0.1097030984
C	-4.7092573960	0.6374805621	2.9823073751
H	-4.8527310076	1.7113537150	3.1796322146
H	-4.6177806551	0.1218913976	3.9494690216
H	-5.6244764730	0.2700699004	2.4920627194
C	-0.1670268034	-3.5461557810	1.8527570226
H	0.5039295674	-2.8166681976	2.3289380879
C	0.3691821448	-3.8015241779	0.4398876396
H	1.3999454655	-4.1868032365	0.4877390034
H	0.3728956731	-2.8797898108	-0.1621741874
H	-0.2456173956	-4.5485694795	-0.0872778502
C	-0.1190995736	-4.8384987799	2.6711342935
H	0.9200119194	-5.1940332752	2.7535257858
H	-0.7011745846	-5.6386111264	2.1874839934
H	-0.5155270600	-4.7020959754	3.6875255557
C	2.3720096736	0.9175932506	2.5456330241
H	2.7791491040	-0.1046340396	2.4594033699
C	3.1069676995	1.6736170240	3.6643477158
H	3.0193196202	1.1468997327	4.6280172078
H	4.1833573131	1.7038726519	3.4248081640
C	2.5602939567	3.1027805932	3.7775397787
H	3.0822643609	3.6312130302	4.5931134024
C	2.7825221481	3.8429347957	2.4561102960
H	3.8608737671	3.9055003882	2.2304278221
H	2.4071475919	4.8777931380	2.5349069305
C	2.0549078419	3.0956524029	1.3361723540
H	2.2051416511	3.6193652337	0.3769781746
C	2.6049736221	1.6731875736	1.2362616340
H	3.6884947296	1.6951097919	1.0284677690
H	2.1393746285	1.1473308555	0.3915524655
C	0.5577172145	3.0210115085	1.6477556204
H	0.1378885153	4.0372278892	1.7372802354
H	0.0206322976	2.5264878294	0.8219894026
C	1.0588498152	3.0604672332	4.0774931078
H	0.6534673616	4.0861140653	4.1117771639
H	0.8730370421	2.6264959567	5.0722125667
C	0.3261024329	2.2837827231	2.9743513438
H	-0.7543286684	2.2756824169	3.1903479103
C	-0.5293323456	-2.0744221014	4.8286212136
H	-1.4672193919	-2.6414350447	4.7340173202
H	-0.3232701649	-1.9461090971	5.9015477455
H	0.2924544057	-2.6596031016	4.3982786387
C	-1.7639350529	0.0672623316	4.8890580834
H	-2.7244514843	-0.4506821310	4.7625186427
H	-1.8660096760	1.1007027513	4.5347553603
H	-1.5461277990	0.1024507551	5.9664624466
C	-1.1601141238	-0.3370696092	-2.8738015585
C	-2.4479703667	-0.7839276865	-2.5441095150
C	-3.4554669895	-0.8099845257	-3.4894299984
H	-4.4430630900	-1.1668690182	-3.1878219263
C	-3.1996499546	-0.3991439633	-4.8122808721

H	-4.0099041387	-0.4281737105	-5.5446153214
C	-1.9305792340	0.0196813405	-5.1874701354
H	-1.7242503974	0.3188589458	-6.2182543348
C	-0.9110256316	0.0436357373	-4.2235606503
C	0.4913471851	0.3685728765	-4.2710883730
C	1.3543494279	0.7783585973	-5.2989772889
H	0.9718692469	0.9427108951	-6.3095807993
C	2.7009238262	0.9591833240	-5.0135995755
H	3.3929882198	1.2770262405	-5.7969446680
C	3.1968080529	0.7149730723	-3.7181355689
H	4.2595628951	0.8227286823	-3.4890281382
C	2.3421438184	0.3127493600	-2.7100414538
C	0.9683884822	0.1568380939	-2.9455131044

Complex 1, Excited state S1.

C	-0.0880485129	0.1135338833	1.9105858429
N	-0.7486832468	-0.8446661733	2.6863233035
F	-2.8495459468	0.1241641442	-1.5890358834
Au	-0.0673183845	-0.0320188951	-0.0855137478
N	0.0094841088	-0.1540360894	-2.1930947570
F	2.7910203815	-0.4221053635	-1.2282588848
C	0.7681557668	0.9724357995	2.8377023848
C	0.9228460001	0.0198549598	4.0448554223
H	1.8047421381	-0.6158594487	3.8584655297
H	1.0904140824	0.5239071192	5.0059910355
C	-0.3211619419	-0.8775364208	4.1048480429
C	-1.8454742245	-1.5939322033	2.1678407315
C	-1.6419984293	-2.9038582144	1.6597548833
C	-2.7442780492	-3.6301439880	1.1899290018
H	-2.5899897866	-4.6435862708	0.8088318787
C	-4.0219370974	-3.0849930774	1.1799638573
H	-4.8690115392	-3.6691233150	0.8099030609
C	-4.2089057786	-1.7815961832	1.6270355225
H	-5.2097898888	-1.3419948934	1.5952980549
C	-3.1434130523	-1.0169045563	2.1155836060
C	-3.4330938958	0.4131794628	2.5404737789
H	-2.4999559702	0.8263890233	2.9427017468
C	-3.8372036330	1.2848486340	1.3481492719
H	-4.0061421027	2.3233479581	1.6760509008
H	-4.7722204897	0.9245218017	0.8886839616
H	-3.0622075911	1.2954259858	0.5690487258
C	-4.5059359675	0.4936310131	3.6289480061
H	-4.6140120644	1.5315458457	3.9826835714
H	-4.2622048106	-0.1359708897	4.4970849640
H	-5.4892620784	0.1737072108	3.2480951414
C	-0.2684059489	-3.5428680423	1.5314793431
H	0.4586126187	-2.8430059533	1.9670592376
C	0.1115246049	-3.7376080645	0.0597445452
H	1.1349394929	-4.1382095293	-0.0247116730
H	0.0748422399	-2.7840409572	-0.4910123699

H	-0.5676091057	-4.4479265298	-0.4395893475
C	-0.1663326824	-4.8773553005	2.2739036660
H	0.8667897172	-5.2585045082	2.2385754902
H	-0.8128969105	-5.6404049592	1.8112234685
H	-0.4580622474	-4.7873997229	3.3299878232
C	2.1655828248	1.3602882873	2.2795534310
H	2.6844999223	0.4313443534	1.9840453133
C	2.9850718957	2.0804099572	3.3620744536
H	3.1246145871	1.4349317077	4.2443674451
H	3.9943575901	2.2971262613	2.9701922110
C	2.2918683763	3.3878338003	3.7649699630
H	2.8824424007	3.8947255267	4.5478587808
C	2.1656024199	4.3012417593	2.5425556818
H	3.1657156439	4.5626539711	2.1546493841
H	1.6730668685	5.2471320427	2.8286459749
C	1.3522699815	3.5865179698	1.4594015816
H	1.2558012604	4.2404624244	0.5751532671
C	2.0704878898	2.2938704103	1.0693348115
H	3.0875952135	2.5207617705	0.7027753148
H	1.5403900922	1.7926536978	0.2413921316
C	-0.0388344541	3.2396624951	1.9983528686
H	-0.5740842320	4.1634056336	2.2808977615
H	-0.6388249015	2.7423882719	1.2181874274
C	0.8905059209	3.0755461510	4.2961389623
H	0.3700160647	4.0147697765	4.5541189642
H	0.9547521364	2.4929930375	5.2290636248
C	0.0824526864	2.3283602565	3.2254180982
H	-0.9289057865	2.1253361155	3.6118956784
C	0.0468234775	-2.2798774529	4.5898284762
H	-0.8268532664	-2.9494053663	4.5834506005
H	0.4120324379	-2.2201896518	5.6266608778
H	0.8414979330	-2.7284631776	3.9789958919
C	-1.3835765728	-0.3349836923	5.0699709057
H	-2.2971176988	-0.9469669607	5.0288036285
H	-1.6564852266	0.7070734365	4.8615291835
H	-1.0001159503	-0.3820151732	6.1008071020
C	-1.0225487510	-0.0625231030	-3.0828288556
C	-2.3993229787	0.0685158891	-2.8306788910
C	-3.2991161290	0.1278395852	-3.8887166990
H	-4.3624673859	0.2325278664	-3.6660942311
C	-2.8356069566	0.0492668782	-5.2025104157
H	-3.5560194634	0.0966356976	-6.0216732244
C	-1.4665912274	-0.0965783405	-5.4907336451
H	-1.1235518972	-0.1674595344	-6.5247755756
C	-0.5741915991	-0.1524752802	-4.4345529284
C	0.8676270192	-0.3097987721	-4.3390917654
C	1.8782617805	-0.4461065925	-5.2741823153
H	1.6608459652	-0.4504195855	-6.3440351930
C	3.2007985109	-0.5774912881	-4.8149963290
H	4.0136234378	-0.6870714180	-5.5357866564
C	3.5004245768	-0.5681003467	-3.4526920724

H	4.5273234239	-0.6656766306	-3.0958295030
C	2.4823199392	-0.4318732449	-2.5146216384
C	1.1465324256	-0.3037127110	-2.9387289959

Complex 1, Excited state T1.

C	-0.0532922913	0.0009834105	1.9899124346
N	-0.8271081480	-0.7669863954	2.7188054710
F	-2.8966034088	-0.7659755587	-1.3629414366
Au	-0.1110534278	-0.0793386638	-0.0220570792
N	-0.1120119495	-0.1741959007	-2.1023290364
F	2.7268529082	-0.2658011963	-1.3200874809
C	0.8275004790	0.8524961060	2.8998896668
C	0.7255784400	0.0805461653	4.2315877098
H	1.5683747277	-0.6271367669	4.2873030999
H	0.7819724134	0.7188995533	5.1198142015
C	-0.5819921836	-0.7083191290	4.2110576406
C	-1.8518221133	-1.6194341427	2.1526805190
C	-1.5211544483	-2.9370806201	1.7600469729
C	-2.5506238918	-3.7463862960	1.2666055344
H	-2.3198578203	-4.7686067625	0.9574283242
C	-3.8522108150	-3.2749009075	1.1495889465
H	-4.6394993790	-3.9283705955	0.7650006557
C	-4.1437030131	-1.9622314223	1.4987551697
H	-5.1606954437	-1.5849578093	1.3681634072
C	-3.1590494757	-1.1030783362	1.9984838915
C	-3.5334389740	0.3452675069	2.2710342055
H	-2.7008516339	0.8170487309	2.8048745284
C	-3.7124984026	1.1229012717	0.9646803947
H	-3.9388434632	2.1790212193	1.1815190802
H	-4.5401578641	0.7107000044	0.3670651508
H	-2.8014814652	1.0778167783	0.3500009203
C	-4.7782078776	0.4770997236	3.1498241676
H	-4.9419362977	1.5330264683	3.4161657713
H	-4.6896294231	-0.1003484311	4.0823622789
H	-5.6813498855	0.1292560672	2.6244730748
C	-0.1093531074	-3.5035080192	1.7717728341
H	0.5534752472	-2.7617569397	2.2411194788
C	0.3983690781	-3.7254352713	0.3426154044
H	1.4445081909	-4.0698132448	0.3581199801
H	0.3514530469	-2.7995959479	-0.2519553391
H	-0.2000179682	-4.4910222367	-0.1765267980
C	-0.0071890080	-4.8049024941	2.5705926981
H	1.0422961038	-5.1347226347	2.6215342721
H	-0.5818638896	-5.6124002301	2.0905981542
H	-0.3801149239	-4.6919546801	3.5985214407
C	2.3141959198	0.9919431313	2.4692204951
H	2.7406132606	-0.0205408689	2.3648382117
C	3.0680278821	1.7591602036	3.5677978627
H	3.0182446215	1.2280439108	4.5316323118
H	4.1356087100	1.8114117310	3.2954412677

C	2.4964974724	3.1762522233	3.7045720775
H	3.0337234218	3.7124038778	4.5049608840
C	2.6582155667	3.9264294285	2.3801936379
H	3.7268202803	4.0143255649	2.1193268238
H	2.2620906404	4.9520690482	2.4756574800
C	1.9118161393	3.1668025440	1.2811294517
H	2.0191973241	3.6975543532	0.3201428680
C	2.4903197610	1.7583498821	1.1567684518
H	3.5665644981	1.8056906716	0.9180259995
H	2.0140298180	1.2248994619	0.3220583113
C	0.4268837562	3.0579189142	1.6404393889
H	-0.0123604227	4.0642869817	1.7457383063
H	-0.1260714159	2.5535881749	0.8308675289
C	1.0068405588	3.1019159940	4.0530364752
H	0.5814876332	4.1187238394	4.1029795844
H	0.8604577726	2.6624126956	5.0518974558
C	0.2544307194	2.3136086309	2.9720359420
H	-0.8171934760	2.2779425876	3.2262062712
C	-0.4138668161	-2.1025273413	4.8014350226
H	-1.3331357177	-2.6995672717	4.7112027162
H	-0.1882412792	-1.9932322142	5.8723937115
H	0.4187743189	-2.6467756143	4.3389812531
C	-1.7100167600	-0.0013795034	4.9605221290
H	-2.6650910847	-0.5342714640	4.8540497570
H	-1.8434632681	1.0416610080	4.6453463542
H	-1.4518772131	0.0045056487	6.0295341202
C	-1.2116474471	-0.1705105674	-2.9249964274
C	-2.5462453240	-0.4242050075	-2.6064286811
C	-3.5609097221	-0.3702703790	-3.6082351448
H	-4.5965909296	-0.5630022744	-3.3261633937
C	-3.1836283381	-0.0797220455	-4.9086548781
H	-3.9575853589	-0.0366441618	-5.6810930595
C	-1.8372636106	0.1573117930	-5.2829401415
H	-1.5866168274	0.3749504469	-6.3226546036
C	-0.8345397754	0.0997601278	-4.2834210970
C	0.5790978434	0.2246081090	-4.2744353987
C	1.5688551543	0.4573633190	-5.2592355289
H	1.2962569743	0.6285667412	-6.3023882291
C	2.9212859262	0.4594269123	-4.8690557571
H	3.6872218940	0.6381420917	-5.6299960943
C	3.3312187749	0.2361207082	-3.5557447761
H	4.3822361562	0.2270579272	-3.2652849745
C	2.3380754256	0.0071411025	-2.5734029742
C	0.9837479206	0.0234265547	-2.9062513013

Complex 2, Ground state S0.

Cu	-0.0030453697	-0.0119787852	-0.0202258960
F	-1.4723022664	2.4785124766	-0.7439338829
N	1.0220614171	0.3485150560	2.6586121866
F	0.7898055907	-2.5977281909	-1.5923783184

C	-1.1854379151	-0.3146673283	2.7798106053
N	-0.2032619805	0.1281051215	-1.9300861487
C	0.0097497570	0.0129382320	1.8976890922
C	-0.6450077219	1.2438909000	-2.5906042449
C	-0.7788835399	0.2823945746	4.1432021970
H	-1.2275503315	1.2842456661	4.2360903182
H	-1.1216622153	-0.3008081275	5.0063240930
C	0.7457475358	0.4167339746	4.1459809853
C	-1.3716270296	-1.8697752931	2.7975863114
H	-0.4249720366	-2.3450231351	3.1036004025
C	2.6583255312	1.9647981306	1.7570415349
C	1.6407636514	3.0879104425	1.6488669760
H	0.6845983402	2.7299750280	2.0582250459
C	2.3400763845	0.6432876362	2.1426493518
C	-1.7730972883	-2.3916122415	1.4097520689
H	-0.9919017181	-2.1704463203	0.6623227690
H	-1.8664147398	-3.4901535109	1.4549193821
C	2.9456527319	-1.8641095072	2.2518340191
H	1.9797059216	-1.9213089671	2.7671407081
C	0.1252055310	-0.1423705800	-4.2182554300
C	-2.9578756998	-0.2504832328	0.9213950078
H	-2.2365997250	0.0401157550	0.1394119045
H	-3.9175926369	0.2147586753	0.6380386679
C	3.9717534180	2.2319665959	1.3527283594
H	4.2361259053	3.2481619108	1.0499531114
C	-0.8575802426	2.2043031551	-4.8487843222
H	-0.7113283693	2.1300344988	-5.9295741800
C	3.2912510595	-0.3986833731	2.0500594459
C	-0.4628236114	1.1552862200	-4.0029215596
C	0.5319890350	-0.8560862598	-5.3573767891
H	0.4448375106	-0.4097764934	-6.3515044560
C	1.4022665940	3.4210981390	0.1730926637
H	0.6149959228	4.1839037138	0.0723188643
H	1.0825833104	2.5302495107	-0.3871239282
H	2.3187081264	3.8119096582	-0.2969090530
C	0.2409648650	-0.7120107130	-2.9150182492
C	-3.1052257863	-1.7716811489	0.9764686153
H	-3.3799069899	-2.1529102062	-0.0215740789
C	1.4313454887	-0.7101617399	4.9156346642
H	2.5251458036	-0.6590110092	4.8198407296
H	1.0875969823	-1.7041276918	4.6004311270
H	1.1860299791	-0.5929781534	5.9814859894
C	4.9364454786	1.2338386187	1.3112819890
H	5.9573652454	1.4682908338	0.9988671810
C	-2.5324432593	0.2855879492	2.2912740327
H	-2.4220972828	1.3825519274	2.2401653041
C	-1.2555225722	2.3952303667	-2.0704024033
C	0.7299455620	-2.0220005187	-2.8071345325
C	-3.6279715672	-0.0787911584	3.3048562254
H	-3.3902772316	0.3215385254	4.3035490580
H	-4.5753597201	0.3931361710	2.9941369752

C	-2.4778138323	-2.2503928639	3.7889428240
H	-2.5827349805	-3.3487014466	3.7956426894
H	-2.2118763273	-1.9609526463	4.8176108339
C	4.5879433963	-0.0709153475	1.6384897898
H	5.3365560955	-0.8625181875	1.5577069023
C	1.0376944265	-2.1399079015	-5.2023436708
H	1.3596707100	-2.7139236558	-6.0744917556
C	2.7585239133	-2.5285855183	0.8873804552
H	2.4540171682	-3.5810431452	1.0043123605
H	3.6942482655	-2.5068506345	0.3057686274
H	1.9829511634	-2.0125713150	0.3042551322
C	-4.1959592611	-2.1331267891	1.9876396824
H	-4.3291525581	-3.2280080847	2.0293777597
H	-5.1610253419	-1.6996994472	1.6730247082
C	2.0490467971	4.3440177143	2.4198792786
H	1.2461483175	5.0962259642	2.3696868473
H	2.9536230838	4.8033972582	1.9906239414
H	2.2514007928	4.1335808621	3.4801753332
C	1.1307071085	-2.7279591294	-3.9235455015
H	1.5068249903	-3.7457681836	-3.7951084228
C	-3.7998212343	-1.6016789798	3.3681676444
H	-4.5835183434	-1.8528816860	4.1026129853
C	-1.6473480037	3.4277054017	-2.8988900291
H	-2.1214071020	4.3070787261	-2.4563544632
C	1.1954980498	1.7493372334	4.7321406358
H	2.2875914317	1.8743207660	4.6833325503
H	0.9019981704	1.7758811215	5.7921421638
H	0.7134565101	2.5979329786	4.2299526069
C	-1.4424898445	3.3336196510	-4.2915391394
H	-1.7589052717	4.1614383740	-4.9306266594
C	3.9651857627	-2.6216517965	3.1006586818
H	4.1374981060	-2.1321657301	4.0712991980
H	4.9369467051	-2.7093835581	2.5900326797
H	3.6063973048	-3.6445657826	3.2940928396

Complex 2, Excited state S1.

Cu	-0.2517821910	0.0583903517	-0.0334966167
F	-1.5144277672	2.6273430626	-0.7937254081
N	1.0402985059	0.3598558469	2.6433325744
F	0.7071284541	-2.4505686786	-1.5267924062
C	-1.2175887245	-0.3318878184	2.7667658442
N	-0.2175469585	0.2803569236	-1.9285082267
C	-0.0670188486	0.0767811535	1.8571009502
C	-0.6611898156	1.3861946277	-2.6127361061
C	-0.7875821262	0.3136891382	4.1018284455
H	-1.2349260339	1.3197351153	4.1542879921
H	-1.1289813641	-0.2332538864	4.9917561039
C	0.7452318256	0.4500522523	4.0963428657
C	-1.3672409131	-1.8863467037	2.8437829446
H	-0.4012171447	-2.3195267338	3.1516034479

C	2.6850636768	1.9196711181	1.6719143523
C	1.6909521754	3.0657515588	1.5921124400
H	0.7372004418	2.7074531682	2.0071677084
C	2.3323528998	0.6130987838	2.0983887963
C	-1.7497150684	-2.4428022227	1.4666617297
H	-0.9661368867	-2.1876505147	0.7274793642
H	-1.7996280281	-3.5446294847	1.5048429537
C	2.9258251775	-1.8980782137	2.2446425312
H	1.9065206108	-1.9209536234	2.6528523351
C	0.1213741624	-0.0422268316	-4.2046285734
C	-2.9991092007	-0.3468357715	0.9539944354
H	-2.2819755389	-0.0331287067	0.1589449095
H	-3.9614718726	0.0886292072	0.6331137195
C	3.9922819853	2.1597321180	1.2299390161
H	4.2705395521	3.1687651893	0.9133452519
C	-0.8236365152	2.3093946755	-4.8774281723
H	-0.6585905788	2.2260616272	-5.9540834433
C	3.2738551409	-0.4430856229	1.9774372919
C	-0.4584764214	1.2728825074	-4.0161921070
C	0.5384244939	-0.7719633269	-5.3198558995
H	0.4789900706	-0.3429380911	-6.3227682416
C	1.4382918945	3.4357114967	0.1274795055
H	0.6561164753	4.2072280461	0.0505477979
H	1.1070962258	2.5607013419	-0.4508002055
H	2.3510472806	3.8307732149	-0.3471436966
C	0.2125162638	-0.5936531969	-2.8962479856
C	-3.0950875416	-1.8703549159	1.0184695192
H	-3.3595154908	-2.2700797393	0.0247763219
C	1.4113173582	-0.6586853535	4.9152635471
H	2.5070782843	-0.5957727629	4.8502410053
H	1.0961789767	-1.6602236521	4.5929245592
H	1.1352997491	-0.5422687649	5.9747747342
C	4.9363951484	1.1422124060	1.1735687178
H	5.9543676351	1.3519528971	0.8338269105
C	-2.5941811761	0.2159650987	2.3164697123
H	-2.5188523382	1.3151624098	2.2515903955
C	-1.2713991558	2.5398941113	-2.1066530520
C	0.6790975795	-1.9040141005	-2.7478640457
C	-3.6807264323	-0.1745568169	3.3307036375
H	-3.4444199383	0.2420448581	4.3232486609
H	-4.6455944875	0.2654731517	3.0246567898
C	-2.4589263492	-2.2939579813	3.8425945140
H	-2.5287823258	-3.3950619514	3.8828692123
H	-2.2054101604	-1.9628342437	4.8624766279
C	4.5672692353	-0.1500206864	1.5275457797
H	5.2988634049	-0.9581733800	1.4437580210
C	1.0265459478	-2.0649053955	-5.1298595585
H	1.3564450544	-2.6544657429	-5.9882166805
C	2.9100979264	-2.6881509864	0.9337494855
H	2.5808421702	-3.7248928893	1.1115717529
H	3.9153167978	-2.7297661824	0.4830783660

H	2.2283701272	-2.2366910139	0.2011165599
C	-4.1782434294	-2.2545704478	2.0332252529
H	-4.2749873486	-3.3530842426	2.0795291454
H	-5.1566777763	-1.8567094630	1.7121750690
C	2.1335799135	4.3021079326	2.3761015143
H	1.3464479012	5.0727368606	2.3499241895
H	3.0423564148	4.7498449774	1.9421841731
H	2.3453663508	4.0712063323	3.4300721946
C	1.0921002616	-2.6352056245	-3.8481809753
H	1.4543994459	-3.6546226606	-3.6987235600
C	-3.8019340726	-1.7002080845	3.4101485703
H	-4.5812144728	-1.9711144656	4.1430053479
C	-1.6400315902	3.5697366500	-2.9556013874
H	-2.1151200383	4.4578938223	-2.5334084528
C	1.1707027951	1.7911785685	4.6934069351
H	2.2617785959	1.9303815560	4.6409490247
H	0.8798781177	1.8287501215	5.7547305129
H	0.6842065402	2.6312739607	4.1800281372
C	-1.4099188827	3.4538480162	-4.3360729240
H	-1.7062298850	4.2756726981	-4.9918247235
C	3.8672560678	-2.5690587077	3.2461707891
H	3.9103449517	-2.0305906147	4.2039472587
H	4.8940921432	-2.6313908212	2.8515626153
H	3.5335426825	-3.5987578345	3.4515915813

Complex 2, Excited state T1.

Cu	-0.1595678670	0.1045486931	0.0242839298
F	-1.5449827827	2.6207933330	-0.7300248778
N	1.0629672363	0.3626136833	2.6730357522
F	0.7746378178	-2.3934215431	-1.5188174816
C	-1.2129814342	-0.3343224150	2.7703307514
N	-0.2648934880	0.2910989439	-1.9055379741
C	-0.0433092863	0.0842699719	1.8992479664
C	-0.7262612392	1.3933599232	-2.5766422122
C	-0.7938257073	0.3211292589	4.1072405634
H	-1.2614089862	1.3173492708	4.1591513366
H	-1.1335738697	-0.2390500505	4.9894912218
C	0.7400243454	0.4856132257	4.1198034695
C	-1.3469803798	-1.8860129604	2.8364211990
H	-0.3876306838	-2.3105368926	3.1751534505
C	2.7122198073	1.8832407898	1.6501864381
C	1.7300358992	3.0386261434	1.5622326726
H	0.7762841385	2.6993125809	1.9923031253
C	2.3557683561	0.5904141655	2.1135184954
C	-1.6793846718	-2.4302388110	1.4417817375
H	-0.8740244492	-2.1614996903	0.7288737336
H	-1.7183412806	-3.5325116236	1.4640502985
C	2.9198715779	-1.9217110817	2.3209390958
H	1.9057238813	-1.9199221908	2.7415339100
C	0.0614297613	-0.0032631837	-4.1879834632

C	-2.9248885826	-0.3390851151	0.9069593866
H	-2.1801791807	-0.0171444014	0.1390678397
H	-3.8744311215	0.0953281879	0.5500958412
C	4.0153675440	2.0970889663	1.1844375688
H	4.3007055658	3.0943559032	0.8387980275
C	-0.9555540266	2.3283527471	-4.8372148073
H	-0.8127357755	2.2491376719	-5.9179073451
C	3.2824523040	-0.4802360738	2.0084171717
C	-0.5456001046	1.2904772399	-3.9857721472
C	0.4816382364	-0.7266817179	-5.3152880874
H	0.3889910813	-0.2977638951	-6.3163362183
C	1.4670383927	3.3919351865	0.0957619125
H	0.6863281528	4.1641083488	0.0159612341
H	1.1298228260	2.5125607999	-0.4734275835
H	2.3768607485	3.7777486418	-0.3915674236
C	0.1879258128	-0.5563305751	-2.8811615311
C	-3.0132965903	-1.8629519161	0.9562542226
H	-3.2420941885	-2.2557326117	-0.0485062492
C	1.4134634391	-0.5979772757	4.9642976694
H	2.5089754002	-0.5277527311	4.9009094331
H	1.1062308625	-1.6092218570	4.6650238416
H	1.1315685917	-0.4580556529	6.0192285925
C	4.9465750104	1.0671809735	1.1399410181
H	5.9608863808	1.2571281365	0.7786220237
C	-2.5735195604	0.2155621019	2.2866217285
H	-2.5009274279	1.3151300880	2.2346347928
C	-1.3427868326	2.5397844182	-2.0592355553
C	0.7027049024	-1.8513642011	-2.7493245606
C	-3.6862070533	-0.1906704747	3.2644883429
H	-3.4809214782	0.2175557474	4.2675294986
H	-4.6433807743	0.2482540431	2.9347552598
C	-2.4669258289	-2.3043215672	3.7982514219
H	-2.5291209289	-3.4056234882	3.8322081895
H	-2.2475197047	-1.9739854816	4.8263640127
C	4.5708576471	-0.2124285689	1.5314637669
H	5.2924950133	-1.0300118593	1.4550171059
C	1.0102502120	-1.9995214551	-5.1395408310
H	1.3424804922	-2.5798152043	-6.0035499432
C	2.8795096798	-2.7503907574	1.0344863050
H	2.5353194716	-3.7755993767	1.2467611083
H	3.8803053667	-2.8224127574	0.5782772754
H	2.2010689059	-2.3105178938	0.2912774717
C	-4.1262132196	-2.2625234655	1.9331540196
H	-4.2163992666	-3.3618470639	1.9667349408
H	-5.0959592890	-1.8685435352	1.5831987512
C	2.1938522218	4.2789699002	2.3271949114
H	1.4154264460	5.0580253433	2.2966851170
H	3.1047906463	4.7100656959	1.8813246723
H	2.4101889726	4.0585518136	3.3823920983
C	1.1172818034	-2.5681548414	-3.8535950316
H	1.5154257794	-3.5755691165	-3.7114304247

C	-3.7988431175	-1.7173358593	3.3262359735
H	-4.5992912279	-1.9998795079	4.0309666233
C	-1.7500020820	3.5623403396	-2.8926184062
H	-2.2294519858	4.4417262690	-2.4562647143
C	1.1373024291	1.8465071399	4.6870153571
H	2.2265889340	1.9989889259	4.6387151191
H	0.8377346776	1.9036461022	5.7448310219
H	0.6420547391	2.6666627541	4.1500422080
C	-1.5507873251	3.4556023414	-4.2846476605
H	-1.8790379118	4.2741006000	-4.9296227955
C	3.8631994748	-2.5712049090	3.3345438104
H	3.9310288982	-1.9954521180	4.2690948802
H	4.8822930691	-2.6699168956	2.9275815139
H	3.5124377582	-3.5848617468	3.5856950959

Complex 3, Ground state S0.

Au	-0.0085593454	-0.0542653031	0.0557072463
N	-2.0633988127	-0.0284450439	-0.0716508196
C	-4.8377816302	1.5653453686	1.6492068467
H	-5.6487977238	2.2454798095	1.3709717901
N	2.7251325691	-0.9252312723	0.6872185595
C	1.9939642810	-0.0471955162	0.0331015418
C	3.1281417400	2.2072129081	0.0534699075
H	3.4237223205	1.9664806524	1.0874422910
C	4.1975772424	-0.9226474971	0.3597082861
C	-4.7267516890	1.1122039746	2.9588189464
C	-3.7966933872	1.3586897053	-0.7309362424
C	-3.9167757163	1.1344353471	0.6861979458
C	1.4345149227	3.4546192965	-1.3257084755
H	0.5118261394	4.0554339963	-1.2820620891
C	2.9193580333	0.8792609227	-0.7565147916
C	3.8225158794	3.4048847310	-2.0412578647
H	4.6365488588	3.9714479645	-2.5238783950
C	1.8482528275	3.0556784921	0.0930807116
H	2.0404583131	3.9558399028	0.7015800655
H	1.0268885015	2.5108246350	0.5845904830
C	-3.1426329973	1.2403091033	-3.4064509396
H	-2.9228014722	1.1635865609	-4.4771755564
C	-2.8563959604	0.2574161429	1.0325096101
C	4.1975353135	0.0185172066	-0.8465793352
H	5.1272927519	0.5973534939	-0.8956486099
H	4.1432320741	-0.5789460149	-1.7712920047
C	5.0590180546	-0.4185312559	1.5179408436
H	4.9439647033	-1.0543400674	2.4072054833
H	4.8423627053	0.6201698736	1.7948597663
H	6.1147176881	-0.4666711778	1.2128421019
C	1.7106135687	-3.1011943879	1.2995232032
C	-4.5572032436	2.0879343873	-1.6529341209
H	-5.4183315898	2.6730708969	-1.3159955129
C	2.4315056174	1.3086944822	-2.1677964583

H	2.2311166888	0.3972764585	-2.7582968382
C	-2.7647501035	-0.2603987243	2.3425351053
C	3.5509575870	2.1234290314	-2.8394899510
H	4.4734923324	1.5293568029	-2.9398902340
H	3.2375757032	2.3827014388	-3.8643227552
C	3.3575049963	0.1023759109	4.6688829751
H	3.6674438790	1.1411069678	4.8624966876
H	4.2660772407	-0.5035065743	4.5328891441
H	2.8530088532	-0.2555417857	5.5797771336
C	-4.2230681711	2.0535431539	-3.0020452331
C	-1.3205710830	-0.4522604198	-3.0446234456
H	-1.3098580971	-0.4566577811	-4.1441558991
H	-1.5214290301	-1.4801275264	-2.6998211654
H	-0.3039989687	-0.2034911824	-2.7006903836
C	2.1112012147	-1.3791424376	3.0214792838
C	-5.6828001329	1.5580474081	4.0303094562
H	-6.4970998132	2.1687532253	3.6134055838
H	-5.1765865026	2.1641267124	4.8009053077
H	-6.1411260285	0.7000626208	4.5491001167
C	-2.6714427346	0.6012821664	-1.1499958703
C	2.5531966331	4.2600203056	-1.9911901395
H	2.2506075512	4.5571043697	-3.0096509302
H	2.7452118566	5.1906863420	-1.4298427152
C	-4.9981768686	2.8359905731	-4.0257198121
H	-5.3693631040	2.1892286238	-4.8378549845
H	-4.3753964124	3.6151218629	-4.4969910664
H	-5.8673600427	3.3368483576	-3.5748447842
C	2.4318532947	0.0441535829	3.4527954761
H	2.9468644553	0.5398277785	2.6210996738
C	4.2309445777	3.0443441614	-0.6098162988
H	4.3790780473	3.9644255286	-0.0191662917
H	5.1995853150	2.5208119725	-0.6037089082
C	4.6840810689	-2.3185286687	-0.0130211070
H	4.6023144243	-3.0164947898	0.8329863617
H	5.7452562987	-2.2539376159	-0.2956800447
H	4.1351321144	-2.7309839315	-0.8687564398
C	1.1743630729	2.1862196601	-2.1352797946
H	0.8969643022	2.4441354247	-3.1711865662
H	0.3143890783	1.6431598919	-1.7141717128
C	-3.6992151950	0.1952199575	3.2689394853
H	-3.6475796021	-0.2028508572	4.2885523068
C	-2.3617059027	0.4959975416	-2.5251254095
C	2.1764299927	-1.8227392267	1.6801562551
C	1.5754674413	-3.5623971180	-0.1429561354
H	2.0056285913	-2.7820068315	-0.7890587429
C	1.6515466371	-2.2813023145	3.9858117701
H	1.5932555260	-1.9607666646	5.0281862564
C	2.3095608773	-4.8773221804	-0.4148742375
H	2.2526933488	-5.1318091211	-1.4846709766
H	1.8508066450	-5.7106734664	0.1396090589
H	3.3704803311	-4.8358514330	-0.1298226804

C	-1.7692174414	-1.3197317078	2.7034173381
H	-1.8711720620	-1.6203921994	3.7560654092
H	-0.7299065730	-0.9910364257	2.5462141686
H	-1.9044555655	-2.2159814175	2.0756653208
C	1.2495268177	-3.5653585812	3.6428350493
H	0.8973501388	-4.2545233257	4.4140984773
C	1.2617510180	-3.9569871807	2.3110429240
H	0.8969096082	-4.9502389828	2.0405322280
C	0.0983066678	-3.6986954657	-0.5300757777
H	0.0083612210	-3.9753489896	-1.5924217863
H	-0.4563546729	-2.7605428865	-0.3762715669
H	-0.3974198183	-4.4846246811	0.0612863791
C	1.1526898639	0.8454427422	3.7140052233
H	0.5205139569	0.9058968307	2.8149808895
H	1.4039912387	1.8730500550	4.0210748673
H	0.5526228810	0.3912827176	4.5175005581

Complex 3, Excited state S1.

Au	-0.0177198012	0.1300728145	0.0776107012
N	-2.1776490870	0.1802248448	-0.1028272440
C	-5.3147421236	1.3261236449	1.2577074056
H	-6.2641214054	1.6966898636	0.8614639591
N	2.6869957459	-0.9522219864	0.5978663084
C	1.9958194490	0.1956518936	0.1912627861
C	3.6073866189	2.2216858374	0.3248345730
H	4.0355165519	1.7752133692	1.2357314050
C	4.0495111356	-1.0766925463	0.0313689894
C	-5.1319941539	1.2037475762	2.6501862182
C	-4.1648608872	0.9035109899	-1.0306823618
C	-4.2878912399	0.9585077579	0.4122645332
C	1.9519024061	3.9242083084	-0.4916868782
H	1.1809151259	4.6505068559	-0.1818629775
C	2.9772789108	1.0908559718	-0.5633137433
C	4.1256336170	3.6288457590	-1.6849020253
H	4.9259010830	4.1429187204	-2.2450068361
C	2.5352374579	3.2329619977	0.7435112467
H	2.9820237901	3.9830474389	1.4196778754
H	1.7394114607	2.7200783865	1.3067821733
C	-3.3418609966	0.4877316516	-3.6266464824
H	-3.0359322716	0.3110307879	-4.6613833054
C	-3.0391140697	0.4783219866	0.9153785660
C	4.0263731652	0.0496112390	-1.0109341415
H	5.0362276838	0.4519697279	-1.1681041591
H	3.6950042189	-0.3681269005	-1.9764116410
C	5.1628940994	-0.8854397236	1.0712651161
H	5.0760514540	-1.6260262854	1.8807229930
H	5.1619812884	0.1162463671	1.5178456892
H	6.1435867524	-1.0324774426	0.5930930951
C	1.4960515961	-3.0066472851	1.2976022281
C	-5.0274723553	1.2241877442	-2.0586288661

H	-6.0218699331	1.6289285250	-1.8505313634
C	2.3804943833	1.8231866334	-1.7951115663
H	1.9133470966	1.0610860863	-2.4427184995
C	-2.8575073715	0.2971869774	2.3126891313
C	3.4853912570	2.5530286333	-2.5713834212
H	4.2526620966	1.8496801178	-2.9362687357
H	3.0496216558	3.0233709294	-3.4703222936
C	4.0444347996	-0.3001965793	4.4399613202
H	4.5607082985	0.6545689674	4.6302025600
H	4.7947112674	-1.0276641268	4.0965558720
H	3.6576214837	-0.6558966364	5.4082925656
C	-4.6125741834	1.0246375797	-3.3921586611
C	-1.1361711523	-0.4928845998	-2.9708660217
H	-0.9615490567	-0.4490244248	-4.0543619369
H	-1.1221649331	-1.5499988885	-2.6632139064
H	-0.2939857777	-0.0160871495	-2.4445488075
C	2.2919589141	-1.4172751671	2.9847084489
C	-6.2131808582	1.6293965636	3.5949399263
H	-7.2126085493	1.4214155112	3.1845491344
H	-6.1609126409	2.7161730970	3.7807937357
H	-6.1250830164	1.1264594176	4.5680359953
C	-2.8560588731	0.3878934103	-1.2809457468
C	3.0629272444	4.6447892408	-1.2590702636
H	2.6433225364	5.1535700480	-2.1443766917
H	3.5202121543	5.4271352989	-0.6282165908
C	-5.5239283763	1.3832296796	-4.5259988665
H	-5.1085786710	1.0751928734	-5.4950931428
H	-5.6953669106	2.4719346835	-4.5638101256
H	-6.5116305740	0.9092567737	-4.4098299311
C	2.9244070018	-0.1051977169	3.4159672660
H	3.3575348418	0.3517769145	2.5195081147
C	4.7111248284	2.9734953146	-0.4321748677
H	5.1452917221	3.7454637315	0.2276250367
H	5.5421914507	2.3028333719	-0.7027117119
C	4.3007037136	-2.4256748008	-0.6411456289
H	4.2886376555	-3.2478574678	0.0898594700
H	5.2952239275	-2.4194468521	-1.1138111894
H	3.5595287135	-2.6359555804	-1.4234151263
C	1.3268677540	2.8617341403	-1.3960811243
H	0.9133357453	3.3302676353	-2.3066708488
H	0.4850321304	2.3693303099	-0.8809034938
C	-3.9213950391	0.6822501900	3.1323156827
H	-3.8072061021	0.5503324478	4.2116779421
C	-2.4373452748	0.1445554716	-2.6133504465
C	2.1709544477	-1.8018837492	1.6228012423
C	1.1817432887	-3.4276385330	-0.1286266903
H	1.5929612156	-2.6503011228	-0.7887411376
C	1.7903849683	-2.2621839174	3.9794914162
H	1.8895309153	-1.9661562450	5.0273004387
C	1.7993902111	-4.7793816814	-0.4957048034
H	1.6264489504	-5.0073682151	-1.5599292591

H	1.3442427865	-5.5951270700	0.0889679898
H	2.8822487332	-4.8093532123	-0.3147957668
C	-1.6395815091	-0.3297969604	2.9074839744
H	-1.7453203348	-0.4362984974	3.9951758353
H	-0.7350679750	0.2583540645	2.7017095782
H	-1.4523170685	-1.3257210575	2.4786068952
C	1.1761300181	-3.4683793954	3.6658063311
H	0.8038358602	-4.1224076414	4.4585283017
C	1.0286246304	-3.8255606705	2.3331382575
H	0.5214504152	-4.7606236707	2.0805496306
C	-0.3298124971	-3.4779304938	-0.3718757459
H	-0.5396606515	-3.7259022272	-1.4252306771
H	-0.8048544113	-2.5119413948	-0.1437474205
H	-0.8142157284	-4.2492674583	0.2480869812
C	1.8789162951	0.8774664636	3.9450735655
H	1.1465385968	1.1203672096	3.1625416291
H	2.3588466280	1.8183683536	4.2587910596
H	1.3411416172	0.4675471727	4.8154619821

Complex 3, Excited state T1.

Au	-0.0185654113	-0.0550227281	-0.0213968146
N	-2.1724365020	0.0827430891	-0.1228343796
C	-5.0634498806	1.4140177081	1.5736244448
H	-5.9335293547	2.0195798565	1.3055824496
N	2.7284705521	-0.9814540783	0.6756211496
C	1.9888365625	0.0290866834	0.0515213050
C	3.3471403546	2.2286185025	0.0865484578
H	3.6750240447	1.9485105337	1.1002922201
C	4.1585900091	-1.0148138186	0.2997157976
C	-4.8919382089	0.9760585320	2.9036284762
C	-4.0249984058	1.2801804070	-0.8053276728
C	-4.1258771781	1.0588269507	0.6250940265
C	1.6796146245	3.6142179276	-1.1830723312
H	0.8069177672	4.2826407444	-1.0863144253
C	2.9653022872	0.9274974054	-0.7058953905
C	4.0222098472	3.4138004111	-2.0195823030
H	4.8466391877	3.9341406258	-2.5371406117
C	2.1343868214	3.1586774777	0.2069759060
H	2.4035523285	4.0341288429	0.8237607489
H	1.3144575729	2.6338387129	0.7224075521
C	-3.3203888010	1.2158380445	-3.4713249936
H	-3.0740416189	1.1732878105	-4.5357876927
C	-2.9757263135	0.2854575279	0.9683873393
C	4.1671646288	-0.0302166897	-0.8758600968
H	5.1409554027	0.4707297667	-0.9575841846
H	4.0186724554	-0.5964396804	-1.8103121449
C	5.0912135831	-0.5707839592	1.4349850169
H	4.9886112706	-1.2317741054	2.3085735500
H	4.9017693825	0.4609290910	1.7568027746
H	6.1373082067	-0.6282191882	1.0968623517

C	1.6767817604	-3.0997821643	1.3765953154
C	-4.8244829658	1.9381424646	-1.7178349120
H	-5.7290887229	2.4624038272	-1.3975751974
C	2.4837761597	1.4210406589	-2.0976070307
H	2.1913452023	0.5351027800	-2.6880139143
C	-2.8185719191	-0.2168253707	2.2840595062
C	3.6202622702	2.1655824156	-2.8161111641
H	4.4921407881	1.5078764866	-2.9619129719
H	3.2819143998	2.4608762679	-3.8246981761
C	3.4990849940	0.1143729899	4.5851814286
H	3.8445715394	1.1463396275	4.7564410122
H	4.3842318762	-0.5105193534	4.3951380521
H	3.0425729991	-0.2332248200	5.5259412947
C	-4.4627427737	1.9269802940	-3.0808664337
C	-1.3426609731	-0.2804307282	-3.0941484047
H	-1.2698255971	-0.2051922996	-4.1874832663
H	-1.4721447027	-1.3409274950	-2.8271624700
H	-0.3847573665	0.0307983057	-2.6507752953
C	2.1134824051	-1.3421156123	3.0321357706
C	-5.8761929371	1.3742436008	3.9604260895
H	-6.9110841879	1.3117368890	3.5906327312
H	-5.7103274914	2.4190718538	4.2741229815
H	-5.7901129621	0.7426392811	4.8552528449
C	-2.8268733134	0.6149594061	-1.2071324999
C	2.8182044156	4.3510132228	-1.8933877167
H	2.4880274446	4.6867868892	-2.8919836384
H	3.1005970082	5.2559990275	-1.3275766000
C	-5.2923145502	2.6680739855	-4.0845840534
H	-5.0348235018	2.3872848520	-5.1149846034
H	-5.1397581888	3.7564933714	-3.9882337169
H	-6.3668814197	2.4818042963	-3.9334708787
C	2.5040597025	0.0725757932	3.4247083422
H	2.9757771615	0.5306559652	2.5477760131
C	4.4728628104	3.0004296457	-0.6161838858
H	4.7221137435	3.8987240283	-0.0239405494
H	5.3997504509	2.4069983922	-0.6689572909
C	4.6274001235	-2.3984779255	-0.1527786588
H	4.5710784466	-3.1311360147	0.6667127799
H	5.6781484434	-2.3416246695	-0.4767832549
H	4.0350582836	-2.7677130370	-1.0002126377
C	1.2934592802	2.3789413298	-1.9962773595
H	0.9747694845	2.6757419046	-3.0113696251
H	0.4329976465	1.8784870009	-1.5229762362
C	-3.7908269620	0.1656290324	3.2142486646
H	-3.6939733592	-0.2083911262	4.2371795676
C	-2.4823461010	0.5367586934	-2.5794032782
C	2.1784781087	-1.8112893673	1.6945121665
C	1.5410968344	-3.6068534750	-0.0503427573
H	1.9866880296	-2.8463659401	-0.7068932884
C	1.6338234094	-2.1990720975	4.0281654594
H	1.5990019879	-1.8473512318	5.0625497711

C	2.2535817542	-4.9410992569	-0.2812124266
H	2.2067061567	-5.2230892231	-1.3452412811
H	1.7774101543	-5.7540606202	0.2899526698
H	3.3118572551	-4.9057577890	0.0129393898
C	-1.7413287559	-1.1769906541	2.6641775265
H	-1.8130543563	-1.4493637157	3.7249808543
H	-0.7371242484	-0.7704877416	2.4775272547
H	-1.8148121167	-2.0982448261	2.0659083994
C	1.2041409594	-3.4869254584	3.7312693408
H	0.8469328097	-4.1469017292	4.5261672325
C	1.2123347965	-3.9195669749	2.4122498576
H	0.8385536522	-4.9185367446	2.1727049028
C	0.0668792111	-3.7335811002	-0.4478460581
H	-0.0209681052	-4.0610463448	-1.4966934908
H	-0.4579186834	-2.7703358146	-0.3510691554
H	-0.4575668578	-4.4751570507	0.1766528659
C	1.2721168087	0.9205899557	3.7504051884
H	0.6064808730	0.9978134120	2.8778883501
H	1.5734913686	1.9423715513	4.0324455433
H	0.7017148955	0.4952158696	4.5919644854

Complex 3b, Ground state S0.

Au	0.0162917614	-0.0398872099	0.0189820767
N	2.7406304713	-0.2261521045	1.0992587765
C	4.3100344589	0.4718445514	-0.4707018722
H	5.1831692391	0.1137854062	-1.0275977098
H	4.4349538235	1.5593389805	-0.3443555375
N	-2.0496811218	-0.0063203894	0.0669418631
C	2.1988265045	-0.5091013142	2.4130124844
C	1.6228730193	0.2522690753	4.6244509334
H	1.4505157855	1.0658316700	5.3324736801
C	1.9818378728	0.5624328864	3.3096946492
C	2.0281858693	-0.0283509514	0.0124997651
C	4.2372235764	-0.1775299686	0.9085378791
C	-2.7919952166	-1.1536372771	0.3033448746
C	2.9626872472	0.2122642333	-1.1754822797
C	1.9954690291	2.0277472978	2.9056358917
H	2.3661632727	2.0967522612	1.8706763467
C	1.9583970221	-1.8478131812	2.7973761157
C	-3.8927254600	-0.8836714084	1.1593285896
C	-2.7205074118	1.0217073643	0.7141068402
C	2.8858267514	2.8984274712	3.7951086267
H	3.9228730454	2.5371599753	3.8451662928
H	2.5014523672	2.9396148376	4.8259141966
H	2.9019428154	3.9325068869	3.4172938044
C	-2.4621015283	2.4106505597	0.6743315349
C	-4.5958883970	-3.1893228895	1.0517624931
C	1.6032312274	-2.0925277686	4.1281222319
H	1.4131040610	-3.1206095241	4.4440719737
C	-2.6290681499	-2.4436406985	-0.2497374245

C	0.5625602158	2.5734302204	2.9128975833
H	-0.1201225504	1.9499625292	2.3191062638
H	0.5340806794	3.5961405496	2.5057621469
H	0.1608645266	2.6126068280	3.9376678662
C	-3.8464983097	0.5295832258	1.4255021280
C	2.9859913980	-1.0312874093	-2.1304008345
H	3.2029351306	-1.9399428602	-1.5442439522
C	-1.3933427763	2.9992365782	-0.1972921383
H	-1.3834045304	4.0962364106	-0.1220866047
H	-1.5553480220	2.7297947616	-1.2543721401
H	-0.3871796322	2.6328341465	0.0679940878
C	4.9332936251	0.6686539354	1.9658733013
H	4.5658380214	1.7021931368	1.9704827542
H	6.0081474475	0.6982254616	1.7319382478
H	4.8201939485	0.2445774714	2.9742150281
C	1.9307465288	-3.0222304173	1.8325579848
H	2.2790684139	-2.6695573297	0.8508340819
C	-4.6695031468	1.3935043328	2.1582381611
H	-5.5308911219	0.9998989528	2.7065117486
C	-1.5960557036	-2.7361786175	-1.2956817722
H	-1.7059955243	-3.7595622961	-1.6828738021
H	-0.5678503274	-2.6324381785	-0.9112966744
H	-1.6853464261	-2.0363427825	-2.1433457240
C	-3.3026357620	3.2325302036	1.4212758597
H	-3.1196731038	4.3125734023	1.3964173921
C	1.4602397854	-1.0606683663	5.0441603669
H	1.1860633443	-1.2771480679	6.0793012124
C	-3.5315899276	-3.4243273308	0.1546175648
H	-3.4251856471	-4.4280002668	-0.2720928128
C	4.8362446250	-1.5822630547	0.9399246741
H	5.9118622531	-1.5140056654	0.7202145356
H	4.3815683967	-2.2533523661	0.1999878476
H	4.7308652167	-2.0349809683	1.9352930537
C	1.6441263284	-1.2114698578	-2.8529918937
H	0.8316387625	-1.3757951832	-2.1301995295
H	1.6985164587	-2.1158170847	-3.4825523148
C	0.4843190755	-3.4984871009	1.6582649911
H	-0.1879304117	-2.6725920736	1.3840672588
H	0.0995553714	-3.9404656893	2.5905860609
H	0.4204028419	-4.2683963026	0.8736940633
C	-4.7792080794	-1.8994910821	1.5369960985
H	-5.6190911905	-1.6768377196	2.2021019581
C	1.2572713993	1.2481702581	-2.8155545823
H	0.4109846432	1.1592204048	-2.1172406556
H	1.0685321713	2.1503224391	-3.4220992026
C	2.5785193708	1.4329206157	-2.0642615801
H	2.5138821696	2.3250586039	-1.4168955523
C	3.6851982204	1.6351092303	-3.1144097696
H	3.4486107166	2.5310523924	-3.7125746092
H	4.6608660517	1.8303993920	-2.6408028046
C	4.0734895011	-0.8393746911	-3.1989728126

H	4.0900644019	-1.7299505873	-3.8498980478
H	5.0785582390	-0.7737143301	-2.7536478398
C	3.7708808064	0.4095978747	-4.0317293180
H	4.5766487573	0.5612383329	-4.7692973351
C	-5.2457757118	3.7195660924	2.9584632012
H	-6.0843073307	3.2059597452	3.4509772866
H	-5.6696497355	4.5049250107	2.3107311274
H	-4.6649573372	4.2326536753	3.7434936084
C	1.3315524346	0.0142796240	-3.7149607239
H	0.3623849117	-0.1319660758	-4.2195721346
C	2.8159614263	-4.1919736218	2.2656738029
H	3.8625389260	-3.8982553015	2.4301419290
H	2.8034599951	-4.9819386795	1.4985705812
H	2.4480907311	-4.6455182691	3.1990598826
C	-4.3950026735	2.7560090520	2.1779673544
C	2.4364387372	0.2134895407	-4.7552687272
H	2.4957565459	-0.6589701627	-5.4284484069
H	2.2128820490	1.0903988000	-5.3866231307
C	-5.5135877583	-4.3155618167	1.4395868206
H	-6.3567194346	-3.9551703742	2.0470350239
H	-4.9870829078	-5.0855350531	2.0288363017
H	-5.9322214533	-4.8219449676	0.5542990889

Complex 3b, Excited state S1.

Au	0.0067018784	-0.0343843987	0.1142259628
N	2.8258771082	-0.2443860945	1.1599424659
C	4.2566114535	0.5365553404	-0.4870295271
H	5.1451581949	0.2753213037	-1.0760574166
H	4.2946579694	1.6253991295	-0.3171786205
N	-2.1372114144	-0.0099618426	0.2579953910
C	2.3526921344	-0.5519298715	2.4686032668
C	1.8208992789	0.1660246159	4.7270843195
H	1.6654234450	0.9693575386	5.4520707737
C	2.1299686301	0.4921476178	3.4022566218
C	2.0126648427	-0.0294785253	0.0364092471
C	4.2730891802	-0.1656373511	0.8721683069
C	-2.9281914037	-1.1247059452	0.3800868874
C	2.9140608536	0.2102388451	-1.1776597053
C	2.1240825427	1.9596993771	3.0107386231
H	2.4611089420	2.0227813235	1.9655783827
C	2.1462757830	-1.8961369408	2.8671260927
C	-4.1247803777	-0.8253514096	1.0988928833
C	-2.8498980242	1.0471616729	0.7645678908
C	3.0358097994	2.8293469063	3.8786148796
H	4.0718440331	2.4626716687	3.8948704413
H	2.6829401958	2.8678976827	4.9215543388
H	3.0475071854	3.8652036634	3.5033674099
C	-2.5494768294	2.4328895493	0.7080938922
C	-4.8349766497	-3.1173642144	0.9254749126
C	1.8400745352	-2.1709972627	4.2048059619

H	1.7006925334	-3.2093506291	4.5163913839
C	-2.7208033504	-2.4227851581	-0.1478093796
C	0.6916481798	2.5006641831	3.0584810829
H	0.0142556729	1.8712864392	2.4635706473
H	0.6482479729	3.5288827506	2.6638892991
H	0.3074440255	2.5230898076	4.0913977516
C	-4.0725846854	0.6022629548	1.3531657791
C	3.0177137189	-1.0182263941	-2.1489632946
H	3.2828612390	-1.9150741057	-1.5664780484
C	-1.3998771599	2.9956810821	-0.0634368104
H	-1.3489604087	4.0866012067	0.0524515668
H	-1.5093106363	2.7683321031	-1.1358546009
H	-0.4406990420	2.5572811427	0.2463172869
C	5.0448172519	0.6588347718	1.9009549530
H	4.6742201712	1.6905752145	1.9526528163
H	6.1074044113	0.6981455888	1.6154284408
H	4.9846749900	0.2124436042	2.9053324842
C	2.1467064140	-3.0549672405	1.8856901765
H	2.4537469411	-2.6558714769	0.9095061739
C	-4.9370207690	1.4822078715	1.9724892257
H	-5.8604683634	1.1292201087	2.4394828855
C	-1.5955025867	-2.7585417139	-1.0696302247
H	-1.6084842800	-3.8243896098	-1.3334945988
H	-0.6206479231	-2.5107873775	-0.6265400534
H	-1.6709080893	-2.1706173517	-1.9980757390
C	-3.4544025022	3.2898690406	1.3414923481
H	-3.2467815442	4.3629534861	1.3117269306
C	1.6999135932	-1.1541820134	5.1391485810
H	1.4720456288	-1.3886843246	6.1821860030
C	-3.6901620509	-3.3831524616	0.1627728231
H	-3.5521516451	-4.3936487891	-0.2314269888
C	4.9414132208	-1.5454123383	0.7979871966
H	5.9909155183	-1.4365152612	0.4823355573
H	4.4422994931	-2.2128016012	0.0837478562
H	4.9432166403	-2.0329214629	1.7835316736
C	1.6764699919	-1.2699291531	-2.8481331566
H	0.8967431070	-1.4645884702	-2.0963343068
H	1.7527604565	-2.1728419236	-3.4793826126
C	0.7201229277	-3.5881577289	1.7288204270
H	0.0260260523	-2.7694924792	1.4917625879
H	0.3685217516	-4.0694346537	2.6558677092
H	0.6663120360	-4.3368166345	0.9219039109
C	-5.0567208970	-1.8016646885	1.3850309533
H	-5.9604862286	-1.5665780418	1.9537891972
C	1.1726306310	1.1672292247	-2.8025342587
H	0.3608336231	1.0248982324	-2.0696939237
H	0.9101022811	2.0569528849	-3.4021527656
C	2.4987970144	1.4118006881	-2.0788165742
H	2.4029425813	2.3001095036	-1.4304118671
C	3.5720081999	1.6651837306	-3.1520326750
H	3.2928680761	2.5520919962	-3.7480887777

H	4.5462642953	1.8960618166	-2.6916208811
C	4.0751527220	-0.7829240154	-3.2395813031
H	4.1342219598	-1.6720353435	-3.8921313027
H	5.0805574555	-0.6563408038	-2.8074578502
C	3.6973192494	0.4455419874	-4.0711325758
H	4.4778729921	0.6339878370	-4.8282842321
C	-5.5130083191	3.8309055671	2.6930862776
H	-6.5681677633	3.5247382860	2.6404566897
H	-5.4225875832	4.8423489433	2.2725204153
H	-5.2478448853	3.8965721589	3.7624485473
C	1.2846840614	-0.0626287880	-3.7042212455
H	0.3144064780	-0.2543727493	-4.1945556518
C	3.0877244622	-4.1940686449	2.2801740798
H	4.1233224787	-3.8532427199	2.4194777442
H	3.0926238794	-4.9761264181	1.5038970111
H	2.7660930056	-4.6716091767	3.2193701788
C	-4.6201915170	2.8556229763	1.9894042608
C	2.3587663859	0.1861370582	-4.7666849213
H	2.4445793436	-0.6862797238	-5.4380857716
H	2.0826696787	1.0502576395	-5.3964421080
C	-5.8149386972	-4.2026195939	1.2501410271
H	-6.8521020583	-3.8632138708	1.1049662078
H	-5.7231273084	-4.5081061525	2.3064101789
H	-5.6533477868	-5.0955231817	0.6310764907

Complex 3b, Excited state T1.

Au	0.0038701715	-0.0334131018	0.0944233126
N	2.8179380017	-0.2538257918	1.1549144854
C	4.2563423816	0.5360392265	-0.4817357308
H	5.1469755351	0.2767152594	-1.0684086035
H	4.2948287383	1.6239728030	-0.3062987518
N	-2.1384472903	-0.0069425518	0.2403548223
C	2.3379515245	-0.5605655701	2.4612495265
C	1.7917353243	0.1575701076	4.7160404396
H	1.6316353302	0.9609756120	5.4399359494
C	2.1091943793	0.4837045320	3.3932482855
C	2.0100083112	-0.0314854979	0.0295037179
C	4.2665484092	-0.1733836232	0.8739804785
C	-2.9216909444	-1.1240472154	0.3850247156
C	2.9166968589	0.2145929914	-1.1795209290
C	2.1071022037	1.9514462648	3.0022859263
H	2.4508079265	2.0147331011	1.9592773110
C	2.1303213895	-1.9047690941	2.8588904179
C	-4.1075330883	-0.8253884720	1.1221994113
C	-2.8467470607	1.0504121254	0.7549305998
C	3.0141202636	2.8194658430	3.8767385104
H	4.0496478889	2.4516835977	3.8996326053
H	2.6544404233	2.8575640692	4.9173603470
H	3.0293483949	3.8555870384	3.5023694659
C	-2.5505924447	2.4358582347	0.6873035619

C	-4.8101228813	-3.1213977681	0.9725732983
C	1.8156164025	-2.1796224149	4.1946072360
H	1.6747730734	-3.2179825318	4.5055057292
C	-2.7173750752	-2.4237342382	-0.1397436614
C	0.6751808645	2.4943810225	3.0408579921
H	0.0012511101	1.8670683953	2.4399470833
H	0.6362031530	3.5232571711	2.6475060995
H	0.2835866550	2.5159219951	4.0710306508
C	-4.0571007008	0.6043525254	1.3665085062
C	3.0229814584	-1.0097748759	-2.1556365476
H	3.2849157859	-1.9092830823	-1.5758640801
C	-1.4085465592	2.9945869702	-0.0974537531
H	-1.3586953342	4.0865903204	0.0082262783
H	-1.5228798457	2.7568251253	-1.1670031955
H	-0.4467728869	2.5601193464	0.2118521186
C	5.0336704284	0.6456665323	1.9104964892
H	4.6633939983	1.6773417236	1.9653326159
H	6.0976329604	0.6857995433	1.6303082567
H	4.9683576446	0.1945623330	2.9124291352
C	2.1378952079	-3.0635083465	1.8774141315
H	2.4508265686	-2.6638617996	0.9033386236
C	-4.9158234748	1.4856047703	1.9921069807
H	-5.8312531483	1.1327526247	2.4747345813
C	-1.6054425728	-2.7586564833	-1.0778486595
H	-1.6246070367	-3.8234269025	-1.3455786807
H	-0.6240129313	-2.5152969790	-0.6466583915
H	-1.6913063394	-2.1663459589	-2.0025673935
C	-3.4488577133	3.2943193456	1.3286337117
H	-3.2440858227	4.3677118100	1.2906380121
C	1.6686036933	-1.1626197534	5.1276399238
H	1.4340075681	-1.3968928503	6.1692333898
C	-3.6767928004	-3.3867993077	0.1930728164
H	-3.5403703004	-4.3994143918	-0.1961976426
C	4.9350006966	-1.5529085750	0.7955907975
H	5.9856737757	-1.4427059896	0.4842680707
H	4.4384303105	-2.2170124192	0.0764605641
H	4.9331759020	-2.0452237549	1.7787426010
C	1.6845611807	-1.2574418831	-2.8616272822
H	0.9013856797	-1.4551242800	-2.1140230558
H	1.7631064649	-2.1575715524	-3.4965232598
C	0.7128010274	-3.5978965104	1.7113227092
H	0.0193160247	-2.7795744467	1.4715990659
H	0.3562729038	-4.0810236572	2.6355343526
H	0.6643108399	-4.3453363650	0.9029280291
C	-5.0304825333	-1.8037473227	1.4284709619
H	-5.9258727202	-1.5692404055	2.0106394043
C	1.1827678015	1.1799792910	-2.8072642673
H	0.3674601429	1.0348182669	-2.0788138328
H	0.9234832794	2.0725469553	-3.4040385289
C	2.5060459303	1.4204127587	-2.0768239834
H	2.4082238657	2.3059178407	-1.4249029026

C	3.5837398848	1.6775948141	-3.1446420657
H	3.3073505175	2.5671451945	-3.7380204050
H	4.5563160184	1.9062208373	-2.6795354091
C	4.0852746619	-0.7706937214	-3.2407643301
H	4.1461176394	-1.6569661912	-3.8970043331
H	5.0890862366	-0.6471980704	-2.8040347543
C	3.7120990351	0.4618070646	-4.0684605806
H	4.4961099805	0.6530033171	-4.8213268810
C	-5.4917005973	3.8368585409	2.7036664653
H	-6.5463298865	3.5266289805	2.6654373424
H	-5.4104005862	4.8460106935	2.2757789580
H	-5.2141680635	3.9099396744	3.7693537378
C	1.2975474173	-0.0459636503	-3.7139675662
H	0.3291703791	-0.2345201027	-4.2092472387
C	3.0774108997	-4.2017429114	2.2777528685
H	4.1119024318	-3.8601150972	2.4233720094
H	3.0876003038	-4.9839193155	1.5016624325
H	2.7503550493	-4.6793175434	3.2150565181
C	-4.6041260768	2.8603058217	1.9947990102
C	2.3763464998	0.2064503165	-4.7708296013
H	2.4643028848	-0.6631333853	-5.4456037297
H	2.1036194219	1.0734807362	-5.3980481144
C	-5.7878700412	-4.2050741623	1.3090489088
H	-6.8124545149	-3.9217965508	1.0195901967
H	-5.8046845370	-4.3950868343	2.3950696908
H	-5.5383693072	-5.1489201645	0.8060658976

Complex 4, Ground state S0.

Au	0.0526217872	0.0291699945	-0.0187305601
N	-1.9831919171	-0.2792127015	0.0038900791
C	2.0589772137	0.0787980067	0.0763039755
N	2.6558826950	-0.0120696233	1.2474966039
C	2.8410967526	-0.8853385947	-2.1924629814
H	2.7276169227	-1.8954969009	-1.7604707057
C	1.9406475820	-0.1427176700	2.4968817809
C	3.1188618846	0.1116260623	-1.0266638489
C	0.6147989721	-3.2845334058	1.6036721220
H	0.1319590892	-2.5789022524	0.9107373881
H	-0.1126682734	-3.5079107758	2.3987719538
H	0.8196177587	-4.2169196308	1.0541788786
C	-2.5806736697	0.9943639147	-4.3994051360
C	1.5918481930	-0.5259349549	-3.0014505213
H	1.4619382009	-1.2724559978	-3.8031781950
H	0.6846255863	-0.5834246438	-2.3798247466
C	-1.4950800586	2.1623908625	-2.5650290665
H	-1.2370106783	2.2349600860	-1.5064816731
N	-3.3687544386	-0.1292542291	-4.5984752142
C	-3.6076604381	-1.7294500543	-0.7825541732
C	-4.3709733902	-2.3558993318	-1.7804741726
H	-5.0139018728	-3.2019894922	-1.5231740557

C	4.1616574189	0.0164157659	1.2139006088
C	-3.9888908303	-0.4805623795	-5.8160432205
C	-3.4695334624	-1.9443172805	0.6360604233
C	-5.9826480450	-0.9516514861	-7.1010670902
H	-7.0701421712	-1.0469158749	-7.1528794521
C	-4.3050711760	-1.9233199080	-3.0975007313
H	-4.8752185661	-2.4248363741	-3.8803951946
C	1.6525446441	-1.4422352175	2.9734168280
C	-2.7191356971	-0.6640966044	-1.0969981009
C	-2.1793358178	1.0365507052	-3.0377003184
C	1.5453669241	1.0128127733	3.2067559282
C	4.4178931445	1.5619017132	-2.6532969741
H	4.4823822414	2.5723701314	-3.0909106929
H	5.3778820132	1.3928782019	-2.1401555206
C	-2.2563097196	2.0184314727	-5.2923810589
H	-2.5750243581	1.9794876622	-6.3356980328
C	0.6940611343	-0.4336073136	4.9669143651
H	0.2148372583	-0.5472234057	5.9422963411
C	1.0322836607	-1.5550515871	4.2209353610
H	0.7941213953	-2.5484482132	4.6061604555
C	-5.2078357209	-1.1613768336	-8.2413842874
H	-5.6841792155	-1.4280820008	-9.1878055225
C	-2.7340886920	-0.1345325548	-2.4046501027
C	0.9321422956	0.8334282404	4.4515591910
H	0.6201539930	1.7113966834	5.0224499071
C	1.9637867805	1.8778280461	-2.4630933977
H	1.0882583493	1.8992641180	-1.7982548627
H	2.0672374823	2.8947006427	-2.8782257715
C	-4.0815498763	-2.7996477882	1.5593734692
H	-4.8454539221	-3.5113750133	1.2339618393
C	3.2324140631	1.5290972800	-1.6791674126
H	3.3857274185	2.2854285102	-0.8916945400
C	-1.5456826369	3.1107240451	-4.8004655561
H	-1.2910728863	3.9295622835	-5.4781848714
C	4.3921807972	-0.3105160469	-0.2577611899
H	4.5245980311	-1.3996030764	-0.3646278535
H	5.3103100343	0.1590305457	-0.6291212426
C	1.6684952398	2.4257820695	2.6598224524
H	2.2374947350	2.3758403059	1.7214816640
C	-2.4755600457	-1.0244109253	1.0679408731
C	4.0461038121	-0.8655150224	-3.1469232435
H	4.9732009570	-1.1670959817	-2.6324229815
H	3.8804505224	-1.6098625239	-3.9436985388
C	2.9410179224	0.8946479306	-4.5443973471
H	2.7854961825	0.1827319984	-5.3730953488
H	3.0531695834	1.8941441634	-4.9985185011
C	-1.1812285936	3.1912003792	-3.4482731494
H	-0.6532240577	4.0757061937	-3.0837495270
C	-3.5107988844	-0.7985684761	-3.3819447055
C	1.9151396298	-2.7142806384	2.1797406338
H	2.5565756523	-2.4555934215	1.3246121551

C	-5.3809887396	-0.6031138490	-5.8943463203
H	-5.9835214752	-0.4152005962	-5.0033930535
C	-3.7137075101	-2.7257649473	2.8988524717
H	-4.1919256362	-3.3806039459	3.6316971946
C	4.7967385667	-1.0079307067	2.1457494379
H	4.5172830099	-2.0364440487	1.8879331406
H	4.5335582480	-0.8212280398	3.1972137165
H	5.8902940550	-0.9275475062	2.0549984666
C	1.7397365894	0.8738621764	-3.5971258518
H	0.8210664882	1.1441129797	-4.1417035094
C	-2.1119068118	-0.9526348167	2.4179098064
H	-1.3575173085	-0.2360268081	2.7535845312
C	-2.7397773287	-1.8044332806	3.3206706776
H	-2.4754552303	-1.7489371262	4.3803598736
C	4.6876730547	1.4001848870	1.5979239679
H	4.2907121312	2.1957027663	0.9540641466
H	5.7832187296	1.4023898436	1.4988270742
H	4.4503143758	1.6421451359	2.6428338497
C	4.2065367061	0.5287405751	-3.7642411421
H	5.0778407235	0.5323985834	-4.4408752849
C	-3.2114421815	-0.6999278203	-6.9588800326
H	-2.1246234796	-0.6184297921	-6.8874758733
C	-3.8217903927	-1.0290011683	-8.1668110778
H	-3.2063351234	-1.1975397703	-9.0541008351
C	2.4070479099	3.3674269787	3.6128370402
H	3.3867465628	2.9732587634	3.9218021663
H	1.8231169657	3.5509473708	4.5282985706
H	2.5703871681	4.3450737071	3.1330833688
C	0.2895341933	2.9981939971	2.3161528454
H	-0.2456887234	2.3612266161	1.5960799108
H	0.3922510654	4.0021513746	1.8747984784
H	-0.3401889153	3.0911729526	3.2148634726
C	2.6399892807	-3.7786821767	3.0074794548
H	3.5449736987	-3.3875164698	3.4963197871
H	2.9345438726	-4.6239597498	2.3662928333
H	1.9876386185	-4.1861306704	3.7951328104

Complex 4, Excited state S1.

Au	0.3236229354	-0.9469901920	-0.4432436056
N	-1.6479858000	-1.6245156896	-0.8687957214
C	2.1905113423	-0.3352153798	-0.0223191111
N	2.4230382457	0.4866432355	1.0859130762
C	3.9599128905	-2.0185791673	-0.8920354394
H	3.9816669250	-2.4405268412	0.1272669247
C	1.6201242394	0.4391816567	2.2611488036
C	3.5059388023	-0.5258618976	-0.7854929573
C	2.1834546619	-3.0040323630	2.5446589822
H	1.6315886649	-2.8434152879	1.6066151525
H	1.4825611121	-3.3742225281	3.3110294792
H	2.9381144159	-3.7877229687	2.3685893763

C	-2.6998664115	1.8312804414	-3.8442154435
C	3.0047149629	-2.8422564701	-1.7579716307
H	3.3345715959	-3.8962156870	-1.7704229772
H	1.9923993191	-2.8288369054	-1.3205475484
C	-0.6642235262	1.5461122345	-2.5504289283
H	-0.0546447264	0.9929490098	-1.8322676249
N	-3.9138923215	1.2163553212	-4.0438287921
C	-3.7670902086	-2.1213350617	-1.6137697038
C	-4.9166641022	-1.9448175152	-2.3603640923
H	-5.7361269016	-2.6639964439	-2.2977254328
C	3.6962914187	1.2214284000	0.9796573516
C	-4.9526926862	1.6914312009	-4.8834217218
C	-3.3847177463	-3.1505261149	-0.6729667585
C	-7.1832034854	2.5746248726	-5.1548921561
H	-8.1294752019	2.9008360682	-4.7168576135
C	-5.0245906062	-0.8348663872	-3.2048531489
H	-5.9177870566	-0.6781767825	-3.8113177758
C	1.8208698639	-0.6044782068	3.2005134258
C	-2.6399790168	-1.2043456102	-1.6755088378
C	-1.9386550140	1.0724790263	-2.9092442441
C	0.6200851097	1.4109680663	2.4967165865
C	4.8853400292	-0.0899572017	-2.9009714672
H	4.8367217457	0.3070848294	-3.9300695874
H	5.6211981416	0.5329092280	-2.3679143516
C	-2.2308171652	3.0263390873	-4.4037189888
H	-2.8425877006	3.5957474671	-5.1053694028
C	0.0753015911	0.3337154845	4.6071879745
H	-0.5205782986	0.2970411020	5.5228269641
C	1.0388577139	-0.6373411207	4.3577900092
H	1.1950909425	-1.4395090544	5.0833280513
C	-6.9911672320	2.6382663169	-6.5344543800
H	-7.7893778136	3.0095810858	-7.1813562335
C	-2.7485664729	-0.0586379223	-2.5353209996
C	-0.1282835306	1.3444309229	3.6784417104
H	-0.8979388498	2.0986196419	3.8638398152
C	2.5338325313	-0.8180711327	-3.1324047167
H	1.5053973558	-0.7486174178	-2.7424863653
H	2.5133695767	-0.3929660545	-4.1514184498
C	-4.0099023104	-4.2834144238	-0.1710311309
H	-5.0156968023	-4.5630643092	-0.4944662241
C	3.4920333304	0.0034313915	-2.2595363534
H	3.1606604157	1.0555097358	-2.2606525156
C	-0.9704135071	3.4565507961	-4.0263635011
H	-0.5741347477	4.3855125979	-4.4436232579
C	4.5052073005	0.2436568556	0.1193901964
H	4.9978950919	-0.4776721486	0.7913653641
H	5.3010640408	0.7581971957	-0.4347751750
C	0.2913342039	2.4967062094	1.4912085808
H	0.9547871079	2.3395757957	0.6296375959
C	-2.0851430853	-2.7952942965	-0.2473785530
C	5.3648235818	-2.1085747265	-1.5076656819

H	6.1020560923	-1.5678820750	-0.8916242816
H	5.6902222850	-3.1640400533	-1.5247133366
C	4.3822109195	-2.3679030931	-3.7892602488
H	4.7141081292	-3.4198921791	-3.8386040208
H	4.3754509265	-1.9858570103	-4.8253594123
C	-0.1935140952	2.7203087038	-3.1092680335
H	0.7963492776	3.0859857780	-2.8286039667
C	-3.9623653141	0.0741787624	-3.2611922992
C	2.8497099536	-1.7001761332	2.9849599898
H	3.4903684262	-1.3767635207	2.1536813481
C	-6.1643402390	2.1102990354	-4.3261217723
H	-6.2955938280	2.0779472450	-3.2421275537
C	-3.3239273200	-5.0672338542	0.7694357354
H	-3.7999960078	-5.9620785638	1.1761819410
C	4.3498671509	1.4833024394	2.3318044266
H	4.5755429930	0.5528707972	2.8709238685
H	3.7032982432	2.1003308671	2.9742266072
H	5.2966424447	2.0257120443	2.1858770452
C	2.9793319296	-2.2814918236	-3.1821813778
H	2.2712937664	-2.8620905014	-3.7980587453
C	-1.4057772998	-3.5693933782	0.6895016689
H	-0.4091764492	-3.2853749612	1.0266090024
C	-2.0436321493	-4.7109579015	1.1909284206
H	-1.5257131794	-5.3287178601	1.9276278741
C	3.5195137164	2.5650930201	0.2552922443
H	2.9356561537	2.4407913657	-0.6677422110
H	4.5006119973	2.9866166240	-0.0141867090
H	3.0059657400	3.2999497133	0.8905783480
C	5.3513286000	-1.5483760009	-2.9329792000
H	6.3662293596	-1.6042655272	-3.3635801486
C	-4.7600598911	1.7483477600	-6.2664743270
H	-3.8114282148	1.4100616367	-6.6892576191
C	-5.7779819749	2.2290028023	-7.0870908869
H	-5.6246559695	2.2740831994	-8.1678723196
C	0.5485351960	3.8969423563	2.0497067458
H	1.5789689511	4.0074915529	2.4206524962
H	-0.1263744820	4.1244966366	2.8909020080
H	0.3811815466	4.6633328453	1.2757446348
C	-1.1477475561	2.3692997733	0.9887780871
H	-1.3318571266	1.3620492658	0.5851432323
H	-1.3464203332	3.0995104258	0.1883447193
H	-1.8778249185	2.5495407543	1.7945889756
C	3.7410558379	-1.9298176005	4.2060392522
H	4.2162603898	-0.9974518175	4.5473747072
H	4.5372994310	-2.6522810542	3.9663693506
H	3.1750580128	-2.3422424637	5.0566289213

Complex 4, Excited state T1.

Au	0.1513781114	-0.4979729407	-0.1728199789
N	-1.8997638345	-1.0328451714	-0.3269268626

C	2.1117049037	-0.1322333714	0.0451258665
N	2.5659081354	0.3905823683	1.2584525640
C	3.5560534912	-1.6760527813	-1.4522420205
H	3.6583624792	-2.3385382948	-0.5751753704
C	1.8698960578	0.1693002464	2.4804351261
C	3.2896018608	-0.2280562483	-0.9281365341
C	2.0217503307	-3.3395129894	2.0593115374
H	1.3546096491	-2.9810570974	1.2611333248
H	1.4036925819	-3.7880207860	2.8548060949
H	2.6572331345	-4.1371007870	1.6413821914
C	-2.7066324494	1.6082941910	-4.0582469775
C	2.4089179020	-2.1784211829	-2.3307390694
H	2.6106441303	-3.2183864234	-2.6428533277
H	1.4691411700	-2.1888929349	-1.7543212064
C	-1.1229459397	1.9839116605	-2.2451313881
H	-0.6719719274	1.6952968869	-1.2935517077
N	-3.7245103079	0.7441019176	-4.3977480797
C	-3.8314592334	-1.9108234041	-1.2192887999
C	-4.7882820618	-2.0800526336	-2.2036264305
H	-5.5375902959	-2.8702162181	-2.1201538232
C	3.8961524360	1.0143230450	1.1474683207
C	-4.5184079116	0.8220803304	-5.5691914648
C	-3.5584426478	-2.6148779813	0.0177875535
C	-6.6635349232	1.1369858728	-6.6316893537
H	-7.7355823094	1.3332093387	-6.5543600125
C	-4.7842627328	-1.2419184685	-3.3286733261
H	-5.5221511762	-1.3734402731	-4.1213232131
C	2.0161724463	-1.0733432211	3.1502697379
C	-2.7875072143	-0.9119619649	-1.3295129798
C	-2.1311358362	1.1916156153	-2.8235206996
C	1.0127453365	1.1593277761	3.0156806778
C	4.4231871890	0.6198795686	-3.0615487149
H	4.2944245934	1.2721502843	-3.9431466399
H	5.2870011124	1.0218441188	-2.5080035624
C	-2.2794691382	2.7602164611	-4.7288232237
H	-2.7371242270	3.0640250895	-5.6716195216
C	0.5117267318	-0.2965507283	4.8963692140
H	-0.0089536762	-0.4744993374	5.8409301947
C	1.3288801466	-1.2807426920	4.3497821656
H	1.4418229344	-2.2334718245	4.8747409690
C	-6.0716473981	0.9638881330	-7.8821592264
H	-6.6799750199	1.0183615744	-8.7879123066
C	-2.8314705704	0.0034653729	-2.4255585895
C	0.3571623204	0.9090151503	4.2272038382
H	-0.3010729269	1.6752283304	4.6453743026
C	1.9939252241	0.1535438464	-3.0955527022
H	1.0426174142	0.1923354312	-2.5431638782
H	1.8812698073	0.8231483400	-3.9661636062
C	-4.1889883575	-3.6381818135	0.7104471785
H	-5.0980285163	-4.1062545322	0.3243066769
C	3.1433049144	0.6545201591	-2.2112027109

H	2.9315037629	1.6930838075	-1.9077533160
C	-1.2703115304	3.5043623078	-4.1389987036
H	-0.9170082832	4.4118255106	-4.6346742830
C	4.4739391181	0.2159962025	-0.0270609736
H	4.9722560675	-0.6834351096	0.3721198166
H	5.2438444196	0.7896105105	-0.5595551981
C	0.7322019454	2.4704076953	2.3043583165
H	1.2924289570	2.4506526113	1.3596898530
C	-2.3745784860	-2.0282019284	0.5248069144
C	4.8500652564	-1.7133853792	-2.2816790648
H	5.7155533433	-1.3994188858	-1.6751746943
H	5.0525618682	-2.7519217692	-2.5981807997
C	3.5559655275	-1.3179056262	-4.3801328850
H	3.7601498126	-2.3468434667	-4.7258265161
H	3.4553798900	-0.6903396749	-5.2834671477
C	-0.7015510028	3.1234657125	-2.9067389191
H	0.0856710724	3.7387995182	-2.4665823746
C	-3.8344484071	-0.2184574931	-3.4047818418
C	2.8797790684	-2.1955309212	2.6010807177
H	3.4360566146	-1.7819454559	1.7492918250
C	-5.8895951588	1.0761238389	-5.4746718812
H	-6.3398708086	1.2322996504	-4.4917536522
C	-3.6370097150	-4.0621997269	1.9293929505
H	-4.1220433229	-4.8643171191	2.4900981346
C	4.7273149460	0.8652548681	2.4164087772
H	4.8733744193	-0.1887246941	2.6888455501
H	4.2494036181	1.3751301318	3.2668864217
H	5.7192490222	1.3181102272	2.2654866183
C	2.2638436780	-1.2790569850	-3.5604163180
H	1.4203282516	-1.6303280221	-4.1794581553
C	-1.8281817220	-2.4420245321	1.7381357093
H	-0.9247531229	-1.9730255715	2.1338703851
C	-2.4799441359	-3.4680587442	2.4334931581
H	-2.0705053302	-3.8055547169	3.3880016012
C	3.8086116899	2.5077108085	0.7952090393
H	3.1261893680	2.6786508905	-0.0493982046
H	4.8027762401	2.8896946861	0.5140860917
H	3.4550189645	3.1020050338	1.6490007264
C	4.7123358140	-0.8143409817	-3.5137464915
H	5.6503397311	-0.8376616527	-4.0950350097
C	-3.9236554069	0.6443758186	-6.8216259879
H	-2.8527059999	0.4379366771	-6.8808505660
C	-4.7011127463	0.7230703441	-7.9744450638
H	-4.2328970164	0.5837008371	-8.9517251883
C	1.2014431195	3.6766655203	3.1193099709
H	2.2676847294	3.6038517687	3.3825620096
H	0.6395088445	3.7681758360	4.0631005192
H	1.0532544197	4.6118210550	2.5554824042
C	-0.7508093213	2.6001559189	1.9501894581
H	-1.0875115880	1.7328626630	1.3621483118
H	-0.9318871353	3.5110098144	1.3569977828

H	-1.3806718124	2.6610220454	2.8522398839
C	3.8939215712	-2.7142882543	3.6221590733
H	4.5043724089	-1.9015333004	4.0446754104
H	4.5732681820	-3.4409255382	3.1487338800
H	3.4049903981	-3.2292617685	4.4642116988

References

1 A. L. Spek, *Acta Cryst.* 2009, **D65**, 148.

2 P. van der Sluis, A. L. Spek, *Acta Cryst.* 1990, **A46**, 194.

3 *Programs CrysAlisPro*, Oxford Diffraction Ltd., Abingdon, UK (2010).

4 G. M. Sheldrick, SHELX-97 and SHELX-2018/3 – Programs for crystal structure determination (SHELXS) and refinement (SHELXL), *Acta Cryst.* 2008, **A64**, 112; *Acta Cryst.* 2015, **C71**, 3.

5 W. A. Dollase, *J. Appl. Cryst.*, 1986, **19**, 267–272.

6 R. B. Von Dreele, *J. Appl. Cryst.*, 1997, **30**, 517–525.

7 D. Di, A. S. Romanov, L. Yang, J. M. Richter, J. P. H. Rivett, S. Jones, T. H. Thomas, M. Abdi Jalebi, R. H. Friend, M. Linnolahti, M. Bochmann and D. Credgington, *Science,* 2017, **356**, 159–163.
